# Gut-brain axis: beneficial impact of *Shouchella clausii* spores on fructose induced dysfunction is associated with modulation of the deoxycholic acid – TGR5 pathway

**DOI:** 10.1186/s10020-026-01479-4

**Published:** 2026-05-09

**Authors:** Maria Stefania Spagnuolo, Natasha Petecca, Francesca De Palma, Antonio Dario Troise, Angela Di Porzio, Valentina Barrella, Anella Saggese, Sabrina De Pascale, Marina De Stefano, Andrea Scaloni, Loredana Baccigalupi, Ezio Ricca, Susanna Iossa, Arianna Mazzoli, Luisa Cigliano

**Affiliations:** 1 Institute for the Animal Production System in the Mediterranean Environment, National Research Council, P.Le Enrico Fermi 1, Portici, 80055 Italy; 2 Department of Biology, University of Naples Federico II, Complesso Universitario Monte S. Angelo, Edificio 7, Via Cintia, Naples, 80126 Italy; 3 Department of Molecular Medicine and Medical Biotechnology, University of Naples Federico II, Via Pansini 5, Naples, 80100 Italy; 4 NBFC, National Biodiversity Future Center, Palermo, 90133 Italy; 5 Task Force On Microbiome Studies, University of Naples Federico II, Naples, 80126 Italy

**Keywords:** Sugar, Probiotic, Inflammation, Brain, Bile acids, Synaptic function, Memory, Microbiota

## Abstract

**Objective:**

The increased intake of added sweeteners, such as high-fructose corn syrup, has been associated with a rise in metabolic dysfunctions in gut and brain. While different evidence showed that dietary fructose induces gut microbiota reshaping, the sugar impact on specific bacteria-derived metabolites remains an understudied topic. In this study, we identified secondary bile acids (sBAs) as molecules differentially represented in plasma of rats fed a fructose-rich diet compared to control animals, and hypothesized that these metabolites might be a target for probiotic-based strategies to counteract sugar-induced metabolic disorders. To this aim, we investigated whether probiotic spores of *Shouchella clausii* SF174 ameliorate fructose-induced cognitive and metabolic dysfunctions and prevent molecular alterations in hippocampus and frontal cortex.

**Methods:**

Wistar rats were fed a fructose-rich diet, alone or in combination with the daily administration of *Shouchella clausii* spores, for six weeks. At the end of treatment, behavioral, metabolomic and molecular analyses were performed.

**Results:**

The probiotic spores exerted a protective effect on the memory function of fructose fed rats and prevented the decrease of markers of synaptic plasticity. This was associated with the maintenance of plasma and brain levels of the sBA deoxycholic acid and of its specific receptor Takeda G protein-coupled receptor 5. Further, spores beneficial modulation of fructose-induced peripheral and central inflammation was observed. Also, probiotic spores produced reshaping of the gut microbiota towards a composition exerting neuroprotective and anti-inflammatory effects.

**Conclusion:**

These results suggest that sBAs might act as a communication bridge along the microbiota gut-brain axis and suggest that their modulation, through probiotic administration, represents an effective strategy to counteract fructose-induced neuroinflammation and gut-brain dysfunction.

**Graphical Abstract:**

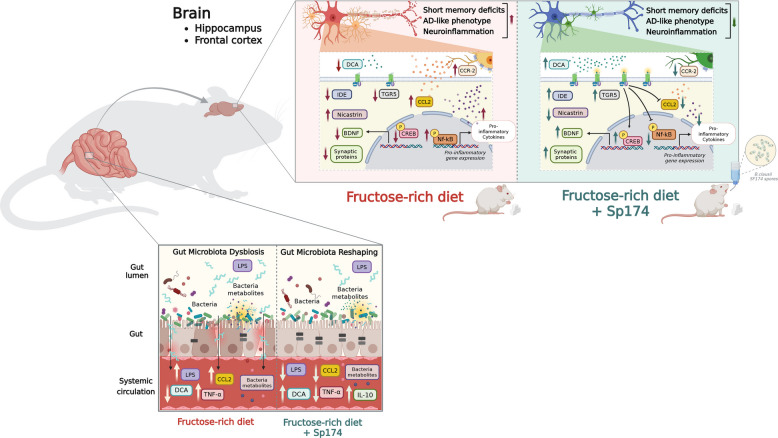

**Supplementary Information:**

The online version contains supplementary material available at 10.1186/s10020-026-01479-4.

## Background

In the current society, the dietary patterns shifted toward an increased consumption of convenient, ultra-processed foods, often linked to unhealthy diet (Lane et al. 2024). By 2020, the intake of added sweeteners (including high-fructose corn syrup, HFCS) has increased by approximately 200% compared to the 1960 s (Cato et al. 2023) with the proportion of HFCS as a major component of sweeteners surging from 0 to 42% between 1966 and 2000 (Bray et al. 2004). This change in dietary habits, especially evident in adolescents and young adults, paralleled the increase of overweight, obesity and metabolic dysfunctions in various organs, including the brain (Spagnuolo et al. 2020a; Sindhunata et al. 2022).

Human and animal studies highlighted the marked effects of fructose on brain structure and functions, also showing cognitive deficits resulting from its overconsumption (Spagnuolo et al. 2020a; Payant and Chee 2021). In this regard, our group previously reported that a short-term fructose feeding induces hippocampal inflammation and affects redox homeostasis in young and adult rats (Cigliano et al. 2018; Mazzoli et al. 2021a). The fructose-driven alteration of key players regulating redox balance, autophagy and synaptic function was also evidenced in the frontal cortex of adults and young rats (Spagnuolo et al. 2020b). Interestingly, alteration of cortical levels of the neurotrophin brain-derived neurotrophic factor (BDNF) and of neurotransmitters, as deriving from fructose feeding during adolescence, persisted even after switching to the control diet (Spagnuolo et al. 2023), pointing out to the adolescence as a critical phase, in which an excessive consumption of sweet foods affects brain physiology also in the long term.

A possible mechanism linking fructose dietary intake to brain dysfunctions can be via the modulation of the ‘microbiota-gut brain’ axis, a bidirectional signalling pathway connecting the gut and the brain, modulated by the gut microbiome (Sindhunata et al. 2022). Several studies have described the effect of the administration of dietary fructose in rodents on the composition of gut microbiota, showing in most cases an alteration of Firmicutes/Bacteroides ratio (Sindhunata et al. 2022). The reshaping of the gut microbiota might subsequently alter the biosynthesis of several metabolites involved in neuroendocrine and neuroimmune pathways, which can both directly and indirectly influence brain function (Schneider et al. 2024). Indeed, a plethora of different molecules can be exclusively produced by the intestinal bacterial fermentation and act as a communication bridge between the gut and the brain. However, despite the evidence that fructose enriched dietary patterns can modulate the composition of gut microbiota (Sindhunata et al. 2022), their impact on specific bacteria-derived metabolites remains an understudied topic.

In this study, we explored the consequences of a fructose-rich diet on the gut microbiota-brain axis of rats focusing on microbiota-derived metabolites. We firstly used untargeted metabolomics to identify molecular classes differentially represented in the plasma of rats fed a fructose-rich diet, compared to control animals. The results of this analysis prompted us to focus on bile acids (BAs) and on pathways activated by secondary bile acids (sBAs), as resulting from the bacterial metabolism. Then, we investigated whether a strategy based on probiotic spore supplementation can ameliorate cognitive dysfunction and molecular alterations induced by fructose feeding in hippocampus and frontal cortex, two critical anatomical districts for learning and memory.

*Shouchella clausii* SF174 spores have been selected as recent data evidenced their anti-inflammatory activity on the gut of a rat model of fructose-induced metabolic syndrome (Saggese et al. 2024) and in a murine model of experimental colitis (Vittoria et al. 2024). The obtained results were indicative of a role for BAs signalling in regulating brain health, supporting the hypothesis that sBAs might act as a crucial communication bridge along the microbiota gut-brain axis in the fructose-induced model of brain dysfunction.

## Materials and methods

### Materials

Bovine serum albumin fraction V (AppliChem, cat A65880), non-fat milk (AppliChem, cat A0830), salts and buffers were purchased from Euroclone (Milan, Italy). Fuji Super RX film (cat 47410), FujiFilm Man-X Developer (cat 949 966), and FujiFilm Man-X Fixer (cat 949 974) were from Laboratorio Elettronico di Precisione (Naples, Italy). Acetylcholine and glutamic acid analytical standards were obtained from Merck (Darmstadt, Germany); *d4*-labelled bile acids internal standard mix (Bile acids SPLASH) was purchased from Avanti-Research (Birmingham, AL). Water, methanol, acetonitrile, formic acid, isopropanol were of mass spectrometry-grade and were obtained from Merck. Canonical unlabeled bile acids (including primary, secondary, conjugated and unconjugated bile acids) were obtained from IROA Technologies (BACMLS, lot 205–36, Ann Arbor, MI).

### Spore preparation and purification

*Shouchella clausii* SF174 (*S. clausii* SF174) cells, isolated from ileal biopsies of healthy human volunteers as previously reported (Saggese et al. 2022) were induced to sporulate at 37 °C in Difco Sporulation Medium (DSM) (Maia et al. 2020) with vigorous shaking, for 30 h. *S. clausii* SF174 spores were harvested by centrifugation (10,000 × *g* for 10 min), washed three times with distilled water and purified as described before (Saggese et al. 2016). Cleaned spores were stored at −20 °C in water. Spores count was determined by serial dilution and plate counting.

### Animals and treatments

The first experiment was performed to carry out an untargeted metabolomic analysis of rat plasma molecules. To this purpose, male Wistar rats (Charles River, Calco, Lecco, Italy) aged 30 days were randomly assigned to two groups of 8 animals each; the control group was fed a control chow diet (C), while the other group was fed a fructose-rich diet (F). The diet treatments were carried out in parallel on the two groups for 6 weeks. The composition of the two diets is shown in Supplementary Table 1.

In the second experiment, to test the potential role of *S. clausii* SF174 in contrasting fructose-induced alterations, male Wistar rats aged 30 days were divided into 3 groups of 8 animals each. The first group was fed a control diet (C), while the other two groups were fed a fructose-rich diet alone (F) or in combination with the daily administration of 0.5 mL of a 10% w/v sucrose solution containing 5 × 10^9^ colony forming units (CFU) of *S. clausii* spores (Sp174). C and F rats received the same amount of sucrose solution without probiotics. Sucrose solution with or without probiotics was presented by an operator each day, at the same hour (15 min before lights out), through a syringe without needle, and voluntarily consumed by rats. The amount of sucrose administered daily is negligible in terms of amount and energy content and was given to all animal groups. In fact, the daily amount of sucrose used for probiotic administration corresponds to 50 mg and 0.84 kJ. This amount represents 0.25% of the daily energy intake of rats and 0.42% of the daily intake of carbohydrates. The diet treatments (Supplementary Table 1) and probiotic administration were carried out in parallel on the three groups for 6 weeks.

All animal experiments were authorized by Italian Health Ministry (137/2022-PR, first experiment; 619/2024-PR, second experiment) and approved by “Comitato Etico-Scientifico per la Sperimentazione Animale” of the University of Naples “Federico II”. The procedures used in this work observe the animal ethics principles and regulations of the Italian Health Ministry. All animals were caged in a temperature-controlled room (23 ± 1 °C) with a 12 h light/dark cycle (06:30–18:30 h). At the end of the diet treatments described above, rats were anesthetized with sodium thiopental (40 mg kg^−1^ intraperitoneal, i.p.) and euthanized by decapitation. The authors ensured that all experimental steps were taken to minimize the pain and suffering of the animals.

Hippocampus and frontal cortex were harvested and dissected as previously described (Mazzoli et al. 2024a,b). Freshly processed aliquots were immediately snap frozen in liquid nitrogen and stored at −80 °C for further analyses. Blood samples were also collected, and plasma was isolated as previously reported (Mazzoli et al. 2024a).

### DNA extraction, high-throughput sequencing and bioinformatic analysis

The cecal content was squeezed out, collected separately and immediately placed onto dry ice.

Total DNA was extracted using the QIAamp DNA Stool Mini Kit (QIAGEN) according to the manufacturer’s instructions. Partial 16S rRNA gene sequences were amplified using the primer pair Probio_Uni (5′-CCTACGGGRSGCAGCAG-3′) and Probio_Rev (5′-ATTACCGCGGCTGCT-3′), targeting the V3 region of the 16S rRNA gene (Milani et al. 2013). The integrity of the PCR amplicons was analyzed by electrophoresis on an Experion workstation (BioRad, UK) (Caporaso et al. 2010). Sequencing of the 16S rRNA gene was performed using a MiSeq (Illumina) at the DNA sequencing facility of GenProbio S.r.l. (http://www.genprobio.com/companyprofile.html) following the protocol described previously (Caporaso et al. 2010). After sequencing and demultiplexing, the reads obtained from each sample were filtered to remove low-quality and polyclonal sequences. All quality-approved, trimmed and filtered data were exported as.fastq files. The latter files were processed using a script based on the QIIME software suite (Caporaso et al. 2010). Raw paired-end.fastq reads were processed using the DADA2 package (v. 1.24) in R. Reads were quality-filtered and trimmed using the parameters truncLen = c(130, 110), maxEE = c(2, 5), truncQ = 2, and maxN = 0, as implemented in the DADA2 pipeline in R. The truncLen parameter truncates forward and reverse reads at 130 and 110 bases, respectively, to remove the low-quality tails typically found at the ends of Illumina reads. The maxEE parameter sets the maximum number of expected errors per read (2 for forward reads and 5 for reverse reads), providing a more robust quality-based filtering threshold than average quality filtering. The truncQ parameter truncates reads at the first position where the quality score drops to ≤ 2, preventing the inclusion of extremely low-quality bases. The maxN parameter discards any reads containing ambiguous nucleotides (N). These filtering parameters were chosen to retain high-quality reads while ensuring sufficient overlap between forward and reverse reads for accurate merging during the denoising step. Error rates were estimated independently for forward and reverse reads, followed by denoising, merging of read pairs, and chimera removal via a consensus-based method. Amplicon Sequence Variants (ASVs) were inferred and used to construct an abundance table. Taxonomic classification was performed using the SILVA reference database (v. 138.1) and the DADA2 native classifier, assigning taxonomy down to the genus level. To normalize sequencing depth across samples, rarefaction was applied at 90,000 reads per sample using the vegan package. Rarefaction curves and read count distributions were assessed to confirm even sequencing effort across groups.

Alpha-diversity (Shannon and Simpson indices) and beta-diversity (Bray–Curtis distances) were calculated on rarefied ASV tables using the *phyloseq* and *vegan* R packages. Differences in alpha-diversity between groups were tested using the Kruskal–Wallis’s test followed by Dunn’s test. Boxplots were used to visualize diversity distributions and inter-individual variability within groups was assessed. Beta-diversity was visualized using Principal Coordinates Analysis (PCoA), and group separation was assessed by PERMANOVA (adonis2, 999 permutations).

Differential abundance analysis was performed at the family genus level using the ALDEx2 R package (Gloor et al. 2017). Count data were transformed using centered log-ratio (CLR) normalization, and differences among dietary groups were tested using the Kruskal–Wallis’s test. For significantly different genera, pairwise comparisons were conducted using Dunn’s test. Results were displayed in bar plots with significance indicated by asterisks.

The 16S rRNA gene sequencing dataset analyzed in this study is publicly available in the NCBI Sequence Read Archive (SRA) database under the BioProject number PRJNA1291627 (https://www.ncbi.nlm.nih.gov/bioproject/PRJNA1291627). BioSample accession number for each sequence is included in Supplementary Table 2.

### Behavioral test

Behavioral analyses were carried out 3 days before euthanasia. The tests were performed between 9:00 A.M. and 13:00 P.M. during the light phase of the 12/12 h light/dark cycle in dedicated testing sound-attenuated rooms. The apparatus used for the novel object recognition test consisted of a sound-proof square arena (40 cm × 40 cm × 10 cm; l × w × h). Light source was arranged to avoid direct illumination of arena, by using multiple light sources to obtain diffuse and low lightening (about 20 lx). The rat behavior was recorded with a video camera. The procedure included three phases: habituation, training, and testing. Animals were brought to the testing room 30 min before the experiment to familiarize with the environment. During the habituation phase, the animal was individually placed in the middle of the empty arena for 5 min; subsequently, each rat took a training trial followed by a testing trial. During the training trial, each rat was individually placed into an open-field arena, containing two identical objects equidistant from each other, and allowed to explore the objects for 5 min. Thirty minutes later, the test session took place, during which one copy of the familiar object and a new object were placed in the same location as during the training trial. Each rat was placed in the apparatus for 5 min, and the time spent exploring each object was recorded. The objects used in this study were different in shapes and colors but identical in size. The objects were fixed on the floor of the box to avoid their movement. At the end of each test, the arena was sanitized by cleaning with ethanol (70% v/v) to eliminate any odor that could interfere with the response of the following rat.

The location preference in the training phase and recognition index (RI) in the testing phase were calculated using the following formulas:

$$\begin{aligned} & \mathrm{Recognition}\;\mathrm{index}\left(\mathrm{RI,}\ \%\right)=\mathrm{Time}\;\mathrm{exploring}\;\mathrm{novel}\;\mathrm{object}/\\&(\mathrm{Time}\;\mathrm{exploring}\;\mathrm{novel}\;\mathrm{object}+\mathrm{Time}\;\mathrm{exploring}\;\mathrm{familiar}\;\mathrm{object})\times100\\&\end{aligned}$$

$$\begin{aligned} & \mathrm{Location}\;\mathrm{preference}\;(\%)=\mathrm{Time}\;\mathrm{exploring}\;\mathrm{one}\;\mathrm{of}\;\mathrm{the}\;\mathrm{identical}\;\mathrm{objects}/\\&(\mathrm{Time}\;\mathrm{exploring}\;\mathrm{the}\;\mathrm{identical}\;\mathrm{objects}\;\mathrm{pairs}\times100\\&\end{aligned}$$

Location preference was used as an environmental control, which should be 50% to rule out the influence of the location of the object.

All recordings were then scored by independent raters that were blind to the experimental conditions.

### Preparation of hippocampus and frontal cortex protein extracts

Aliquots (20 mg) of frozen hippocampus or frontal cortex were homogenized in 7 vol of RIPA buffer (150 mM NaCl, 50 mM Tris–HCl pH 8.0, 0.5% w/v sodium deoxycholate, 0.5% w/v NP-40, 0.1% w/v SDS) containing 1% w/v Protease Inhibitor Cocktail and 1% w/v Phosphatase Inhibitor Cocktail (cat 5871 and cat 5870, respectively; Cell Signalling distributed by Euroclone, Milan, Italy). Homogenates were incubated (30 min) at 4 °C, centrifuged (14,000 × *g*, for 45 min, at 4 °C), and finally protein concentration of supernatants was measured as previously reported (Spagnuolo et al. 2014).

### Inflammatory markers

Plasma, hippocampus and frontal cortex concentrations of tumor necrosis factor-alpha (TNF-α), interleukin-6 (IL-6), C–C motif chemokine ligand 2 (CCL2) and interleukin-10 (IL-10) were assessed using a sandwich enzyme-linked immunosorbent assay (R&D Systems, Minneapolis, MN; TNF-α: cat DY510-05; IL-6: cat DY506-05; CCL2: cat DY3144-05; IL-10: cat DY522), specific for rats, in agreement with the manufacturer’s instructions, as previously reported (Mazzoli et al. 2024a). In detail, for titration of TNF-α, IL-6 and IL-10, plasma samples were diluted 1:10, whereas hippocampus and frontal cortex homogenates were diluted 1:20. For CCL2 evaluation, plasma was diluted 1:40, whereas tissue homogenates were diluted 1:90.

Lipopolysaccharide (LPS) in plasma was determined as previously reported (Mazzoli et al. 2021b), using a protocol based on a Limulus amaebocyte lysate extract (ThermoFisher Scientific, Rockford, IL; cat A39552), in accordance with the kit instructions. In brief, samples were mixed with the LAL reagent, at 37 °C. After 20 min of incubation, the chromogenic substrate solution was added; the resulting mixture was left for 6 min, at 37 °C, and absorbance readings were finally recorded with a plate reader at 405 nm.

### Western blotting

Denaturing and reducing electrophoresis of hippocampal or frontal cortex extracts (Spagnuolo et al. 2018) was carried out on 12.5% T polyacrylamide gels to titrate post-synaptic density protein 95 (PSD-95), synaptosome-associated protein (SNAP25), synaptotagmin, brain derived neurotrophic factor (BDNF), cAMP response element binding protein (CREB), or on 10% T polyacrylamide gels to assay toll-like receptor-4 (TLR4), myeloid differentiation factor 88 (MyD88), nuclear factor kappa-light-chain-enhancer of activated B cells (NFkB), glial fibrillary acidic protein (GFAP), Takeda G protein-coupled receptor 5 (TGR5/Gpbar1), protein kinase B (Akt), extracellular signal-regulated kinase 1/2 (Erk1/2), N-methyl-D-aspartic acid receptor (NMDAR), C–C chemokine receptor type 2 (CCR2), insulin-degrading enzyme (IDE) and nicastrin. Protein blotting onto nitrocellulose membranes (cat RPN3032D, GE Healthcare; Milan, Italy), washing and blocking steps were carried out according to previously published procedures (Cigliano et al. 2018; Spagnuolo et al. 2020b). After blocking, the membranes were incubated with primary antibodies (overnight, at 4 °C), washed and then treated (1 h, at 37 °C) with the appropriate peroxidase-conjugated secondary antibodies. The specific dilution and catalogue number of each antibody is shown in Supplementary Table 3. The amount of phosphorylated NFkB, CREB, Erk1/2, or Akt was expressed as relative to total form of the protein (NFkB, CREB, Erk1/2, or Akt); accordingly, after revelation of the immunocomplexes, the membranes were submerged in stripping buffer (1% w/v SDS, 25 mM glycine, pH 2; for 30 min, at 37 °C) (Mazzoli et al. 2021b), extensively washed, and then incubated with the specific antibody for the total form of the protein (Supplementary Table 3). For loading control, after detection of each antigen, the membranes were stripped and incubated (overnight, at 4 °C) with mouse anti-β-actin IgG (1:1,000 in 0.25% v/v non-fat milk) followed by goat anti mouse (GAM)-HRP IgG (1:350,000 in 0.25% v/v non-fat milk; 1 h, at 37 °C). Signal detection was carried out using the Excellent Chemiluminescent Kit (Elabscience; Houston, TX; cat E-IR-R307). Densitometric analysis of ChemiDoc or digital images of X-ray films exposed to immunostained membranes was performed with Un-Scan-It gel software (Silk Scientific, UT).

### Untargeted metabolomics

Extraction of polar and non-polar plasma metabolites was achieved through the precipitation of 50 µL of each sample in a methanol:water (4:1 v/v) solution followed by incubation at −20 °C, for 1 h. Samples were centrifuged (18,000 × *g*, for 10 min at 4 °C) and supernatants dried in a centrifugal evaporator (SpeedVac, Thermo Fisher Scientific, Bremen, Germany). Aliquots were reconstituted in methanol:water 50:50 (v/v) and analyzed by liquid chromatography-high resolution tandem mass spectrometry (LC–MS/MS) through an Exploris 120 quadrupole Orbitrap high-resolution mass spectrometer and a Vanquish liquid chromatographic system (Thermo Fisher Scientific, Bremen, Germany). The chromatographic flow rate was 0.2 mL/min and the mobile phases consisted of 0.1% (v/v) formic acid (solvent A) and 0.1% (v/v) formic acid in acetonitrile (solvent B). Compounds were separated at 35 °C through a C18 column (Kinetex PS, 100 × 2.1, 2.6 µm, Phenomenex, Torrance, CA) with the following gradient of solvent B (minutes/%B): (0/5), (0.5/5), (9/95), (12/95). Heated electrospray interface (H-ESI) static spray voltage was −3.2 kV for negative ions and 3.5 kV for positive ions, both in the *m/z* range 70–800. Ion transfer tube and vaporizer temperature were 300 °C and 280 °C, respectively, while sheath gas flow and auxiliary gas flow were 45 and 10 arbitrary units, respectively. The analyzer resolution was set at 60,000 (FWHM at *m/z* 200) and profile data were acquired with a normalized automatic-gain control (AGC) target of 100%. Full scan acquisition was combined with differential *m/z* ranges working exclusively and alternatively in positive or negative data dependent scanning mode (ddMS2, *m/z* 70–250, *m/z* 240–550, *m/z* 540–800, resolution 60,000, FWHM at *m/z* 200). For ddMS2 top 4 ion scan, an isolation window of *m/z* 1.0 was used, while normalized collision energy was fixed at 30%, 55% and 80%. Along with procedural blank background mass exclusion list, dynamic exclusion of redundant ions was customized by considering as time window 3.5 s and a mass tolerance of 5 ppm. Mass accuracy was maximized through run start internal calibrant spiking with fluoranthene in positive ion mode (*m/z* 202.0777 [M]^+^) and negative ion mode (*m/z* 202.0788 [M]^−^) in both full scan and ddMS2 experiments (EASY-IC, Thermo Fisher Scientific). Data were collected using Xcalibur 4.5 and Free Style software (v. 1.8, Thermo Fisher Scientific).

An untargeted metabolomic workflow based on molecular classes annotation, metabolites identification and differential accumulation uncovered differences in plasma profiles between fructose fed rats and control rats. Isotopic pattern, elemental composition, exact masses, chemical formulas and fragmentation spectra were matched with an internal library of standards and with information reported in publicly available database, as mzCloud (www.mzcloud.org), ChemSpider (www.chemspider.com), human metabolome database (www.hmdb.ca) and KEGG (https://www.genome.jp/kegg/compound/). After correction for quality control samples and retention time alignment, post-processing nodes performed descriptive statistics and differential analysis. For volcano plots and ratio among sample groups, p-values were adjusted by Benjamini-Hochberg’s algorithm, while hypothesis test was performed by one-way ANOVA model with Tukey as *post-hoc* test. Neural Molecular Network function associated annotated compounds by using mass spectra as reference and similarities based on biotransformation such as glycation, glucuronidation, sulfation, methylation, oxidation, conjugation, isomer interconversion, esterification, deamination, deamidation and secondary conjugation with amino acids, such as glycine, cysteine, tryptophan, serine, glutamine and pyroglutamic acid. Metabolomics workflow, Molecular Network function and multivariate data analysis including principal component analysis (PCA), log fold changes and volcano plots were obtained in Compound Discoverer 3.3 (Thermo Fisher Scientific, San José, CA).

### Bile acids in plasma samples

Bile acids in plasma samples were quantified through the standard addition technique and internal standard correction by using canonical unlabeled, primary, secondary and conjugated bile acids listed in Supplementary Table 4. Calibration curves were prepared in quality control samples by spiking known concentration of canonical and labeled standard mix by using the same chromatographic profile described above for untargeted metabolomic analysis.

### Brain tissue sample preparation for targeted bile acid analysis

For the quantification of bile acids in brain tissues, we referred to Reiter et al. 2021, with some modifications (Reiter et al. 2021). Briefly, 30 mg of brain tissues were mixed with 60 mg of glass beads and 120 µL of cold methanol spiked with *d4*-bile acids internal standard mix. Methanolic suspensions were homogenized twice in a high-frequency bead-beater (Mixer Mill MM400, Retsch, Haan, Germany) for 10 s at 30 Hz for two times. Upon centrifugation (18,000 × *g*, for 10 min, at 4 °C) the supernatants were dried, then reconstituted in acetonitrile and 2 µL injected without any further dilution. Bile acids separation and quantification was achieved in targeted MS2 mode through a C18 column (Kinetex PS, 100 × 2.1, 2.6 µm, set at 40 °C) by using an optimized gradient consisting of 0.1% (v/v) formic acid in acetonitrile (solvent B), in 0.1% (v/v) formic acid (solvent A), (minutes/%B): (0/20), (0.5/20), (3/50), (10/70), (18/97), (22/97) and equilibration step of 7.5 min. Exploris 120 HESI source parameters included a static spray voltage in negative ion mode of −3.2 kV, sheath gas and auxiliary gas were 50 and 20, arbitrary units, while ion transfer tube and vaporizer temperature were set at 300 °C and 320 °C, respectively. Targeted MS2 method included two paired experiments: (i) a full scan negative ion acquisition mode (*m/z* range 100–700) by using an Orbitrap resolution of 60,000, RF lens 70% and profile data type; (ii) a targeted MS2 experiment with a HCD normalized collision energy of 30 for the fragmentation of the bile acid and their respective internal standard listed in the Supplementary Table 4. Quantification was achieved through the internal standard technique by monitoring retention time and isotopic pattern in full scan experiments by using TraceFinder (v. 5.2, Thermo Fisher Scientific).

### Quantification of acetylcholine and glutamic acid in brain tissues

Acetylcholine and glutamic acid were quantified by LC–MS/MS as previously reported (Spagnuolo et al. 2023), with some modifications. Dried aliquots of methanol supernatants (30 µL*, *vide supra) were resuspended in water:methanol 85:15 (v/v), centrifuged at 18,000 × *g*, for 10 min, at 4 °C, and 2 µL analyzed by using a pentafluorophenyl column in reversed phase mode (Kinetex F5, 100 × 2.1, 1.7 µm, set at 35 °C). The chromatographic gradient consisted of 0.1% (v/v) formic acid in acetonitrile:methanol 50:50 (v/v) (solvent B) and 0.1% (v/v) formic acid (solvent A), (minutes/%B): (0/0), (2/0), (9/40), (10.5/98), (14/98). Chromatographic stream was interfaced to an Exploris 120 and the ion source parameters were the following: sheath gas and auxiliary gas 40 and 15, respectively; static spray voltage 3.2 kV; ion transfer tube and vaporizer temperature 320 °C and 310 °C, respectively. Orbitrap resolution was set at 60,000 (FWHM at *m/z* 200); acetylcholine (C_7_H_16_NO_2_, [M]^+^: *m/z* 146.1176) and glutamic acid (C_5_H_9_NO_4_, [M + H]^+^: *m/z* 148.0604) were quantified by screening the precursor ions in Trace Finder software. Calibration curves were built by using pure reference standards through the standard addition to quality control samples with 5 calibration points in the range 1–1,000 ng/mL. Analytical performances were monitored by using accurate masses and an error within ± 2 ppm for precursors ions with at least 5 isotopic pattern signal matching.

### Determination of counts of SF174

Fecal samples (approx. 1 g) from each animal of each group were suspended in sterile PBS and vigorously vortexed thus allowing adequate suspension of solid matter. In order to count SF174 cells and spores, each suspension was divided into two sterile tubes that were respectively treated and untreated with ethanol as previously reported (Hong et al. 2009). The ethanol treatment consisted in mixing 1 volume of homogenized sample (approx. 100 µL) with an equal volume of absolute alcohol (or PBS for not-treated samples). After 1 h of incubation at room temperature, treated and not-treated samples were serially diluted and plated out on solid Difco Sporulation-inducing medium (DSM) supplemented with streptomycin (8 μg/mL), clindamycin (4 μg/mL), erythromycin (4 μg/mL) and chloramphenicol (8 μg/mL) to allow the growth of SF174 cells, resistant to all four antibiotics (Saggese et al. 2022).

Plates were incubated for 2–4 days at 37 °C and about 20 randomly selected colonies for each plate were observed under the light microscope to evaluate the morphology of cells, sporangia and spores.

### Statistical analysis

Data were expressed as mean values ± SEM. GraphPad Prism 9.3.1 (GraphPad Software, San Diego, CA, USA) was used to verify normal distribution of data and to compare groups with one-way ANOVA followed by Bonferroni post-test. A non-parametric Spearman's rank-order correlation analysis was carried out, without multiple comparison correction, to evaluate correlations between parameters, pooling data from all animals across treatments groups. *P* < 0.05 was considered significant throughout the study.

## Results

### Untargeted Metabolomic analysis on systemic plasma

To evaluate the effects of a fructose-rich diet on plasma metabolites, two groups of rats were fed either a standard or a fructose-rich diet (C and F groups, respectively). After 6 weeks of treatment, plasma samples were collected and analyzed by untargeted metabolomics in negative (Supplementary Fig. 1 A,C) and positive (Supplementary Fig. 1 B,D) ion mode to identify metabolites influenced by the fructose-rich diet. The corresponding 2D PCA distributions (Supplementary Fig. 1 A,B) summarize the existence of strong similarities between the circulating compounds in the plasma from C and F rats. Considering the lack of distinct clusters, we moved to the differential analysis through the corresponding volcano plots that highlighted a total of 46 over-represented and 18 down-represented compounds in fructose fed rats, compared to control ones (Supplementary Fig. 1 C,D), by using a Log10 P-value higher than 1.3 (p-value higher than 0.05), and a Log2 fold change higher than 1. Next, to outline the existence of a key molecular class influenced by the dietary intervention, we further screened putative biomarkers through an integrated Molecular Network procedure pinpointing the mass spectrometry similarities and specific biotransformation (Supplementary Fig. 1 E). In line with discriminant analysis, we spotted BAs, including secondary bile acids (sBAs) generated by microbial biotransformation, as a family of compounds modified by the fructose-rich diet.

### Behavioral analysis

**Fig. 1:**
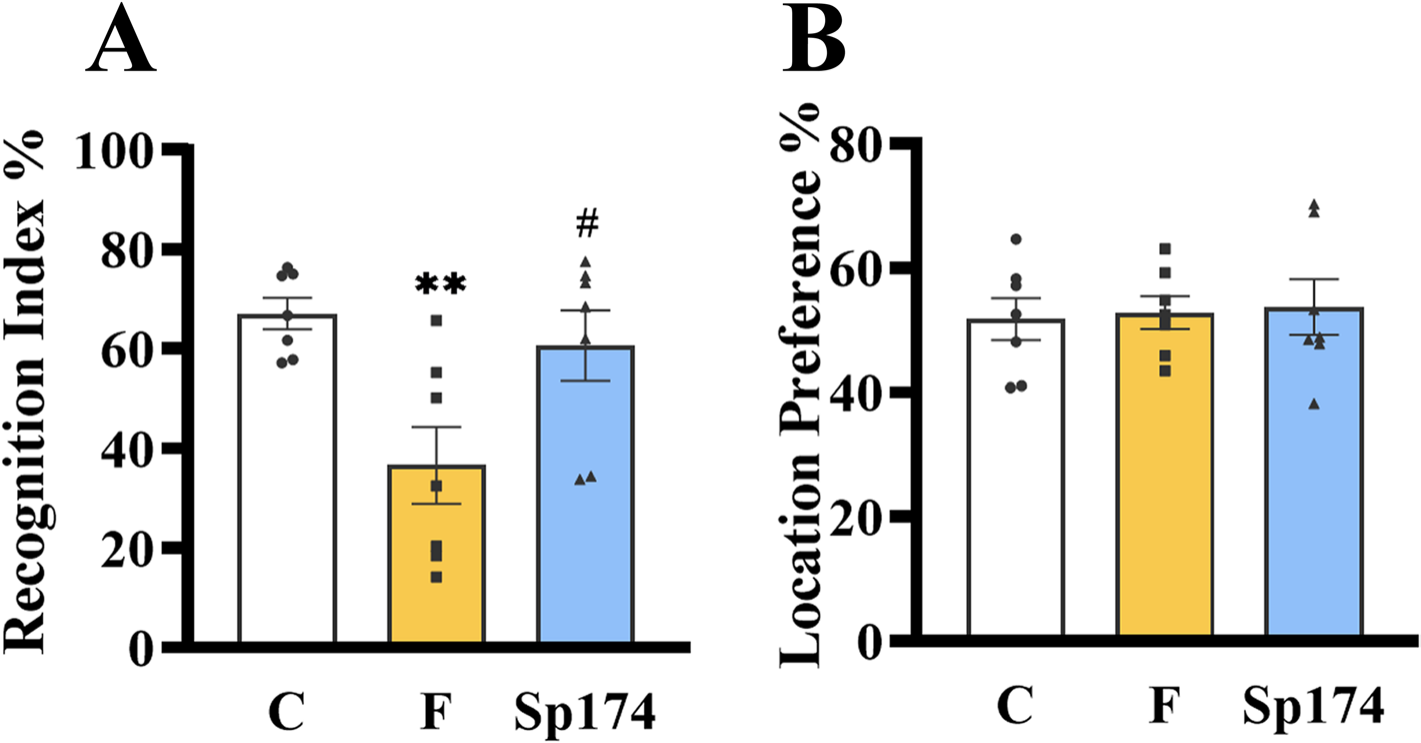
Behavioral analysis. Recognition index (**A**) and location preference (**B**) during Novel Object Recognition test of rats fed control diet (**C**), fructose-rich diet (**F**) or fructose-rich diet and *S. clausii* spores administration (Sp174). The Recognition index was calculated as the percentage of time spent exploring the novel object in the testing session. Location preference was calculated as the percentage of time spent exploring one of the two identical objects within the training session. Reported are the mean values ± SEM of 7 different rats. ** *p* < 0.01 compared to C rats; # *p* < 0.05 compared to F rats (one-way ANOVA followed by Bonferroni’s post-test)

A fructose-rich diet is known to cause cognitive deficits (Spagnuolo et al. 2020a; Payant and Chee 2021). Since sBAs are known to play a neuroprotective role in the brain, based on the previous untargeted analysis, we hypothesized that sBAs may mediate communication between the gut and the brain in our experimental paradigm. Accordingly, we performed a further experiment to test whether the fructose rich diet induces memory defects and to investigate whether the administration of probiotic spores could be a successful strategy to prevent the sugar impact on brain. To this end, rats were fed either a standard diet (C group), a fructose-rich diet (F group) or a fructose-rich diet in combination with the daily administration of 5 × 10^9^ colony forming units (CFU) of *S. clausii* SF174 spores (Sp174 group) for 6 weeks. At the end of the dietary treatment, before the rat euthanasia, the memory function was evaluated by the novel object recognition (NOR) task performed on all rat groups. The NOR index value significantly decreased in the fructose-fed rat group compared with the control one (Fig. 1A), while the administration of the spores of *S. clausii* SF174 prevented the NOR index decrease (Sp174 group). Location preference was used as environmental control, and no difference was observed between the 3 groups of rats (Fig. 1B). The total exploration time of objects during the testing phase was comparable among the different animal groups (data not shown).

### Plasma levels of bile acids and markers of inflammation

**Fig. 2:**
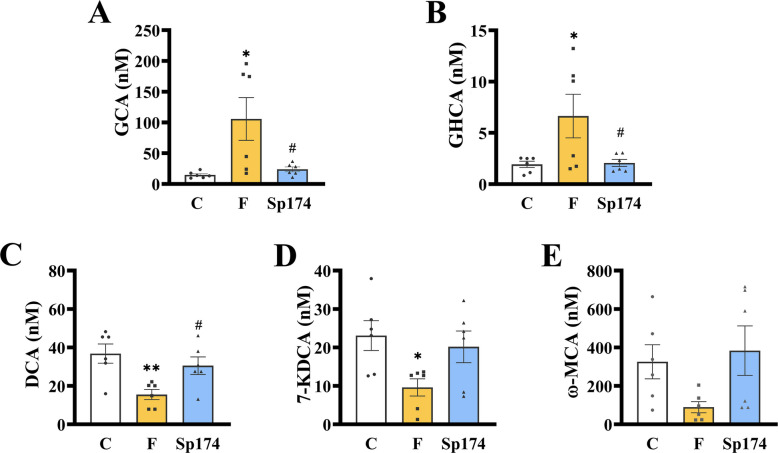
Plasma concentration of Bile Acids (BAs). Concentration of glycocholic acid (GCA; **A**), glycohyocholic acid (GHCA; **B**), deoxycholic acid (DCA; **C**), 7-ketodeoxycholic acid (7-KDCA; **D**) and ω-muricholic acid (ω-MCA; **E**) in plasma samples from rats fed control diet (**C**), fructose-rich diet (**F**) or fructose-rich diet and *S. clausii* spores administration (Sp174). Reported are the mean values ± SEM of 6 different rats. * *p* < 0.05, ** *p* < 0.01 compared to C rats; # *p* < 0.05 compared to F rats (One way ANOVA followed by Bonferroni’s post-test)

BAs include primary and secondary bile acids, with the former ones that can be substrates of microbial metabolic activities and the latter that are directly generated by microorganism biotransformation. Therefore, the fructose effect on plasma sBAs might be in part mediated by the metabolic activities of the intestinal microbiota. To verify this possibility and evaluate whether a probiotic spore treatment can modulate such effects, a targeted quantification of BAs in plasma samples of the three experimental rat groups was performed. As shown in Fig. 2, the levels of some BAs, modified in animals of the F group compared to the C group, were further affected by the probiotic spore treatment. In particular, the concentrations of the conjugated BAs glycocholic acid (GCA) and glycohyocolic acid (GHCA) were higher in the plasma of fructose fed rats than in control animals; this increase was not observed in Sp174 rats (Fig. 2A,B). Interestingly, the decrease of sBAs deoxycholic acid (DCA) and 7-ketodeoxycholic acid (7-KDCA) in fructose fed rats, compared to the controls, was prevented by spore treatment in Sp174 animals (Fig. 2C,D). A similar trend was observed for ω-muricholic acid (ω-MCA), although not statistically significant (Fig. 2E). The concentration of other BAs, such as cholic acid (CA) and β-muricholic acid (β-MCA) (Supplementary Fig. 2 A,B), as well as those of conjugated bile acids, namely taurodeoxycholic acid (TDCA), taurochenodeoxycholic acid (TCDCA) and tauroursodeoxycholic acid (TUDCA) were not affected by the dietary treatment (Supplementary Fig. 2C-E).

**Fig. 3:**
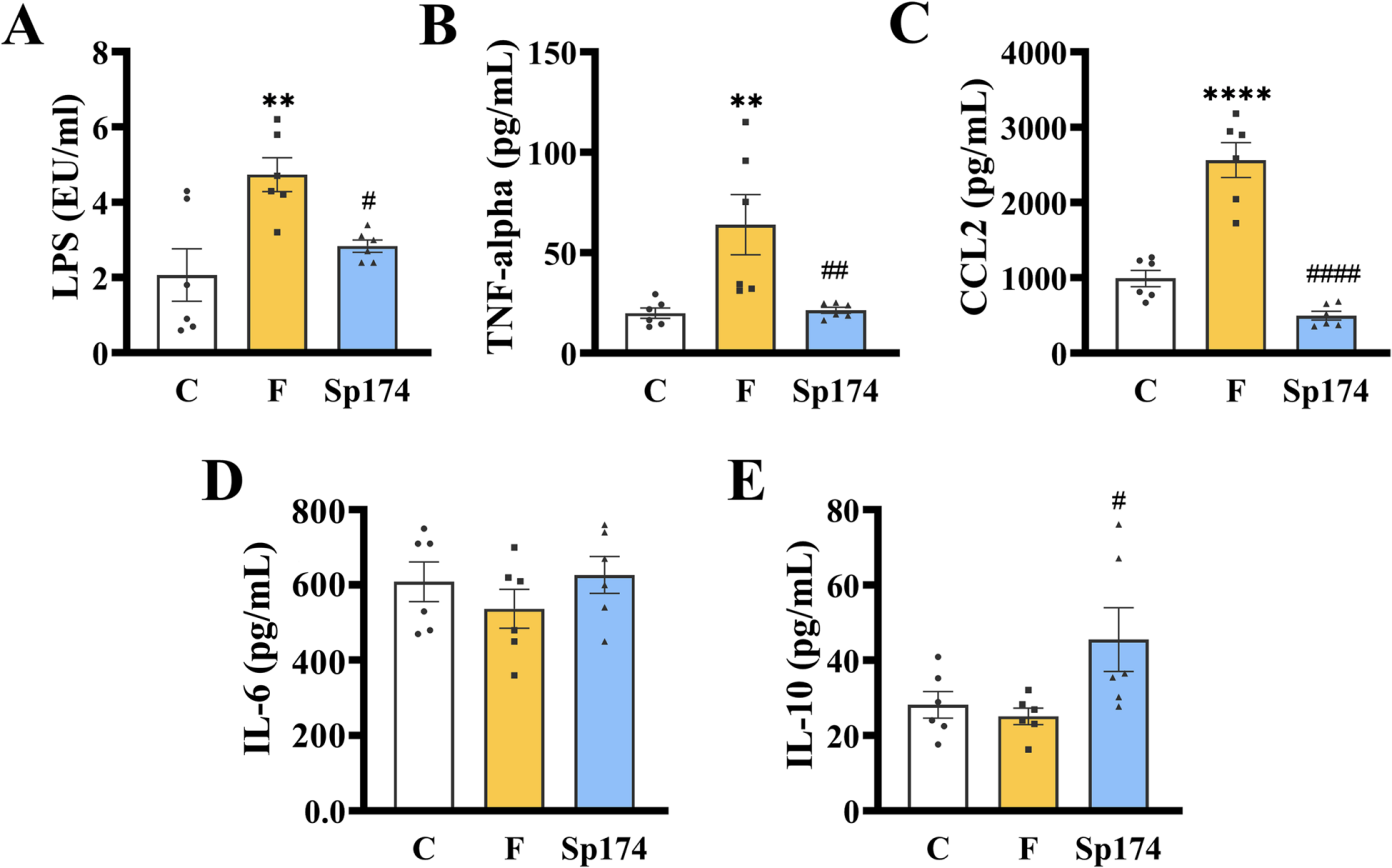
Markers of systemic inflammation. Lipopolysaccharide (LPS; **A**), tumor necrosis factor alpha (TNF-alpha; **B**), C–C motif chemokine ligand 2 (CCL2; **C**), interleukin-6 (IL-6; **D**), and interleukin-10 (IL-10; **E**) concentration in plasma samples from rats fed control diet (**C**), fructose-rich diet (**F**) or fructose-rich diet and *S. clausii *spores administration (Sp174). Reported are the mean values ± SEM of 6 different rats. ** *p* < 0.01, **** *p* < 0.0001 compared to C rats; # *p* < 0.05, ## *p* < 0.01, #### *p* < 0.0001 compared to F rats (One way ANOVA followed by Bonferroni’s post-test)

The analysis of the plasma levels of other molecules in the three experimental groups was further extended to some inflammatory markers. As shown in Fig. 3, the levels of plasma LPS, TNF-α, and CCL2 were significantly increased in fructose-fed rats (Fig. 3A-C), and this effect was prevented by the supplementation of *S. clausii* spores in Sp174 rats. No difference in IL-6 levels was found between the three experimental groups (Fig. 3D). In agreement with the previously observed in vivo anti-inflammatory action of *S. clausii* SF174 spores (Saggese et al. 2024; Vittoria et al. 2024), a higher level of IL-10 was observed in the plasma of Sp174 rats compared to fructose-fed rats (Fig. 3E). These results fit with the evidence that DCA suppresses pro-inflammatory cytokine production from human peripheral blood-derived macrophages (Yoneno et al. 2013).

### Hippocampal and frontal cortex concentrations of bile acids

**Fig. 4:**
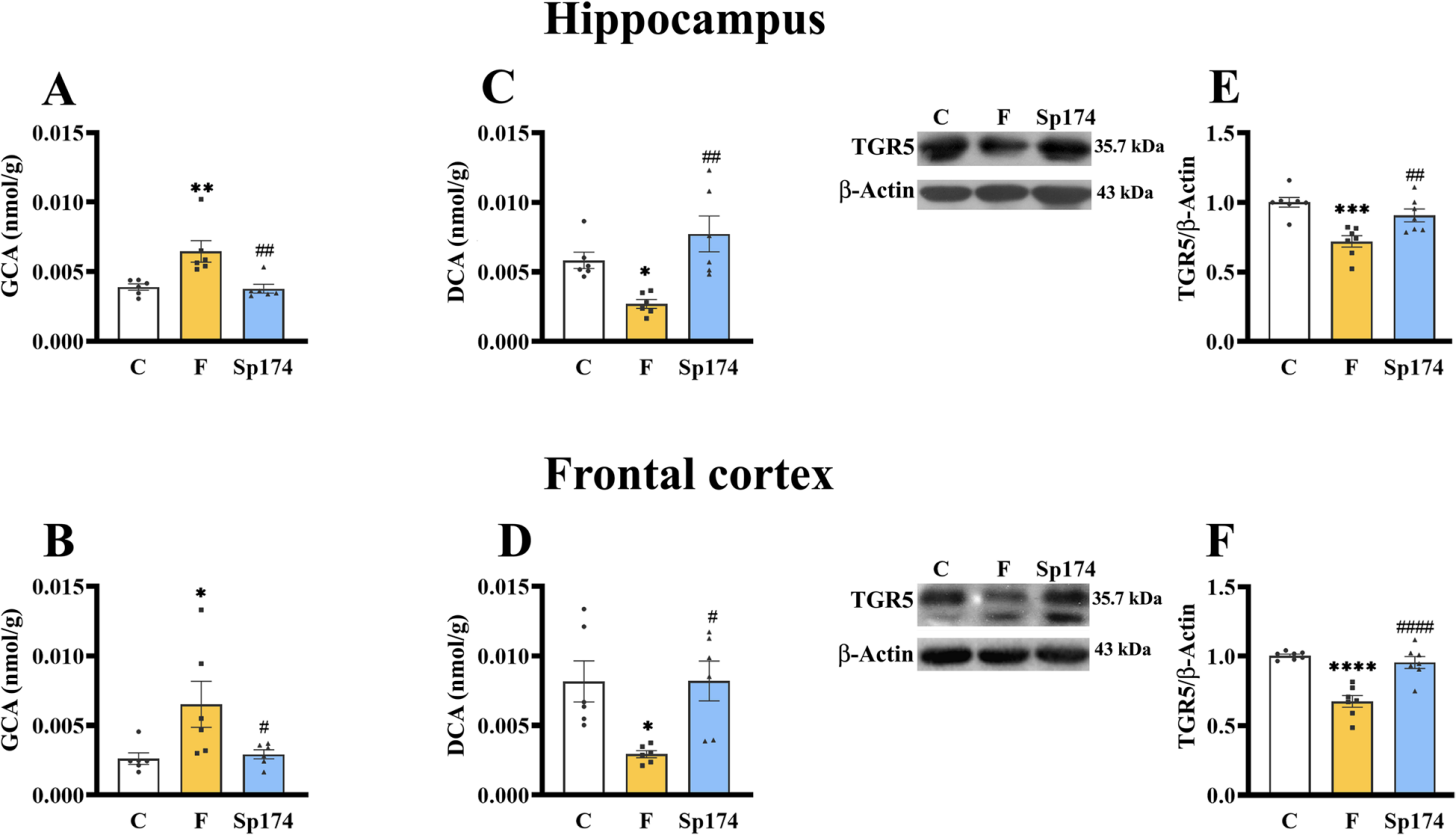
BAs and TGR5 in the hippocampus and frontal cortex. Glycocholic acid (GCA; **A**-**B**), deoxycholic acid (DCA; **C**,**D**), and Takeda G protein-coupled receptor 5 (TGR5) protein content (with representative blots, normalized to controls; **E**,**F**) in samples of hippocampus (**A**, **C**, **E**) and frontal cortex (**B**, **D**, **F**) from rats fed control diet (**C**), fructose-rich diet (**F**) or fructose-rich diet and *S. clausii *spores administration (Sp174). Reported are the mean values ± SEM of 6 different rats. * *p* < 0.05, ** *p* < 0.01, *** *p* < 0.001, **** *p* < 0.0001 compared to C rats; # *p* < 0.05, ## *p* < 0.01, #### *p* < 0.0001 compared to F rats (One way ANOVA followed by Bonferroni’s post-test)

The protection of memory functions by spore supplementation prompted us to explore the concentration of BAs in the hippocampus and frontal cortex of the experimental rat groups. In line with the plasma, a higher concentration of GCA was detected in both hippocampus and cortex of fructose fed rats, and this increase was prevented by probiotic *S. clausii* spore treatment (Fig. 4A,B). On the other hand, no differences between control and fructose fed rats were observed in the levels of taurocholic acid (TCA) (Supplementary Fig. 3 A,B) and tauromuricholic acid (TMCA) (Supplementary Fig. 3 C,D) both in the hippocampus and frontal cortex; likewise, similar amounts of cholic acid (Supplementary Fig. 3E,F) were measured in these tissues from C, F and Sp174 groups.

DCA levels were significantly reduced in both hippocampus and frontal cortex of F rats compared to controls, but this decrease was prevented when fructose fed rats were simultaneously treated with *S. clausii* spores (Fig. 4C,D). BAs modulation of neuroprotective mechanisms, as well as neuroinflammation (Darmanto et al. 2025) is mediated by specific receptors, such as the Takeda G protein-coupled receptor 5 (TGR5/GPBAR1). Accordingly, we investigated the quantitative trend of this protein in C, F and Sp174 groups. The amount of this receptor in the hippocampus and frontal cortex was reduced in fructose fed rats, compared to control ones, and reverted to that of the control animals when rats were also administered *S. clausii* spores (Fig. 4E,F); this finding suggested that the level of TGR5 is modulated by both the fructose diet and the *S. clausii* spore treatment.

### Neurotrophins, synaptic proteins and neurotransmitters

**Fig. 5:**
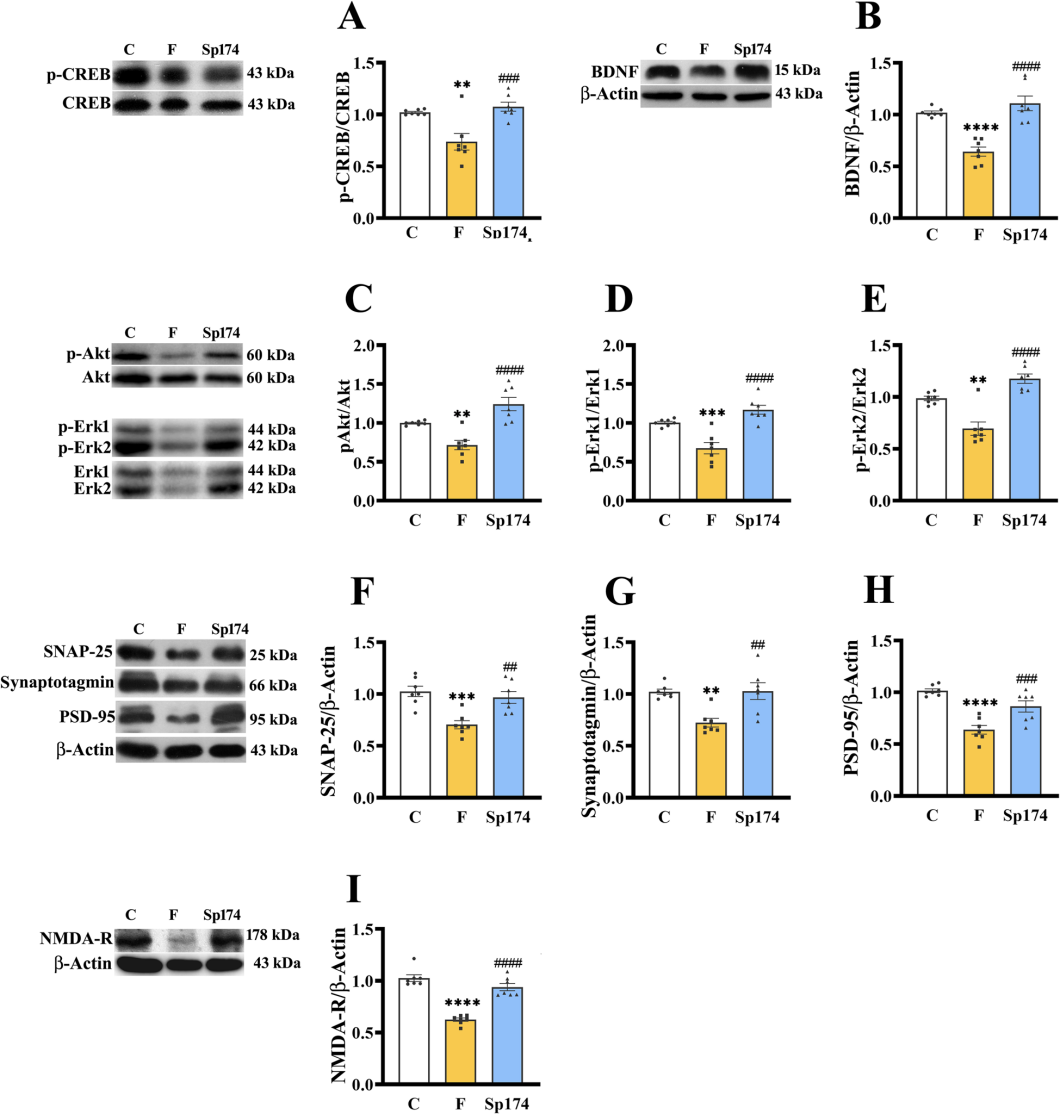
BDNF signalling and synaptic proteins in the hippocampus Phosphorylated cAMP response element binding protein (CREB)/CREB ratio (**A**), brain derived neurotrophic factor (BDNF) (**B**), phosphorylated protein kinase B (Akt)/Akt ratio (**C**), phosphorylated extracellular signal-regulated kinase 1/2 (Erk1/2) (**D**, **E**), synaptosome-associated protein (SNAP25) (**F**), synaptotagmin (**G**), postsynaptic density protein 95 (PSD-95) (**H**), N-methyl-D-aspartic acid receptor (NMDAR) (**I**) (with representative blots, normalized to controls) in the hippocampus from rats fed control diet (**C**), fructose-rich diet (**F**) or fructose-rich diet and S. clausii spores administration (Sp174). Reported are the mean values ± SEM of 7 different rats. ** p<0.01, ***p<0.001, **** *p*<0.0001 compared to C rats; ## *p*<0.01, ### *p*<0.001, #### *p*<0.0001 compared to F rats (One way ANOVA followed by Bonferroni’s post-test)

**Fig. 6:**
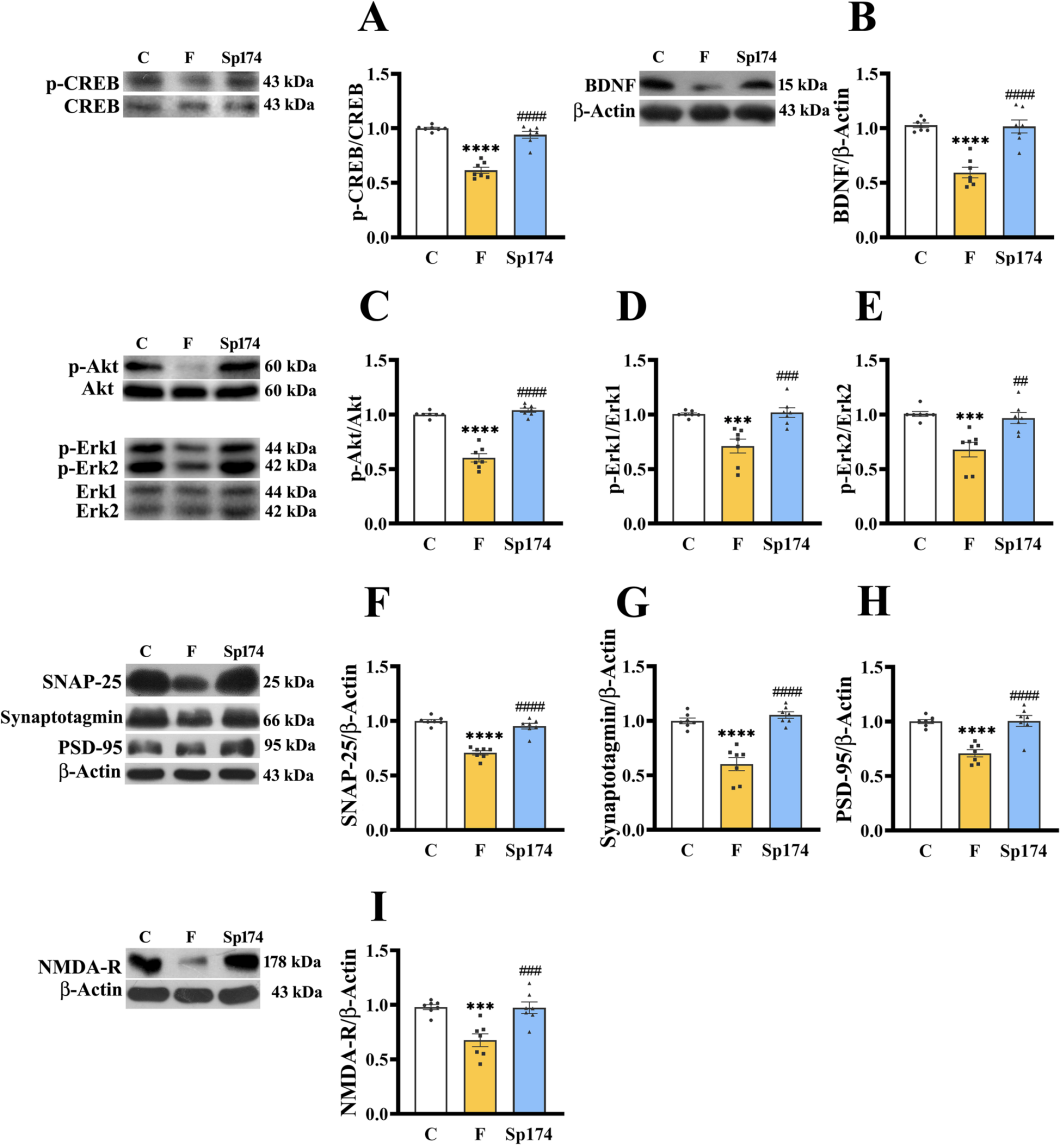
BDNF signalling and synaptic proteins in the frontal cortex. Phosphorylated cAMP response element binding protein (CREB)/CREB ratio (**A**), brain derived neurotrophic factor (BDNF) (**B**), phosphorylated protein kinase B (Akt)/Akt ratio (**C**), phosphorylated extracellular signal-regulated kinase 1/2 (Erk1/2) (**D**, **E**), synaptosome-associated protein (SNAP25) (**F**), synaptotagmin (**G**), postsynaptic density protein 95 (PSD-95) (**H**), and *N*-methyl-D-aspartic acid receptor (NMDAR) (**I**) (with representative blots, normalized to controls) in the frontal cortex from rats fed control diet (**C**), fructose-rich diet (**F**) or fructose-rich diet and *S. clausii *spores administration (Sp174). Reported are the mean values ± SEM of 7 different rats. ***p < 0.001, **** *p* < 0.0001 compared to C rats; ## *p* < 0.01, ### *p* < 0.001, #### *p* < 0.0001 compared to F rats (One way ANOVA followed by Bonferroni’s post-test)

The spore-induced increase of DCA and its receptor TGR5 in fructose fed rats could drive critical signalling cascade that governs synaptic plasticity, gene expression, and memory formation. Indeed, TGR5 activation has been reported to initiate CREB signalling pathway, which in turn modulates synthesis and signalling of the neurotrophin BDNF (Darmanto et al. 2025) involved in a wide range of neurophysiological processes (Kowiański et al. 2018). Accordingly, a significant diet-dependent decrease of the degree of CREB phosphorylation, together with a parallel concentration decline of BDNF, was observed in both hippocampus (Fig. 5A,B) and frontal cortex (Fig. 6A,B) of fructose fed rats. Further, the extent of Akt and Erk1/2 phosphorylation, two downstream players activated by BDNF signalling, was reduced in both brain districts (Fig. 5C-E, Fig. 6C-E, respectively) from F rats, compared to control ones. These modifications were fully prevented by the concomitant administration of *S. clausii* spores in Sp174 rats.

In line with the role played by BDNF in synaptic transmission, we evaluated in both brain regions the amounts of the pre-synaptic proteins SNAP25 and synaptotagmin I (Fig. 5F,G; Fig. 6 F,G), and the post-synaptic protein PSD-95 (Fig. 5H; Fig. 6H), a key player in synaptic plasticity (Won et al. 2017). The level of these proteins was lower in fructose fed rats, compared to control ones, while the concomitant treatment with *S. clausii* spores prevented these alterations (Fig. 5F-H; Fig. 6F-H). To gain further insights into the mechanism underlying the beneficial effect of the *S. clausii* spore treatment, we also evaluated the glutamate receptor NMDAR, which is fundamental for modulation of working memory and synaptic function (Reiner and Levitz 2018). NMDAR was found reduced in fructose fed rats compared to control, with probiotic spores being able to prevent this alteration (Fig. 5I; Fig. 6I).

**Fig. 7:**
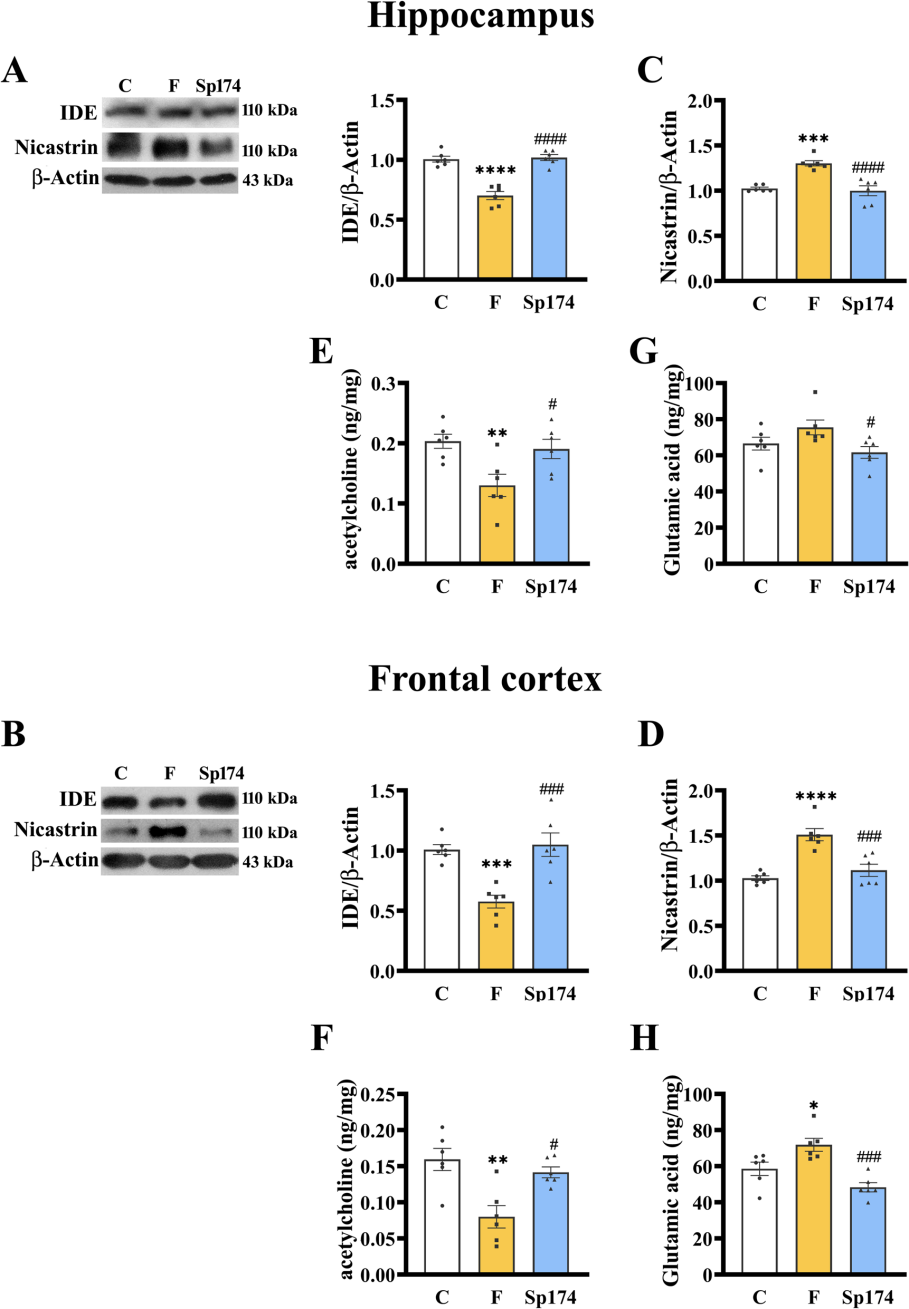
Insulin-degrading enzyme, nicastrin, acetylcholine, glutamic acid in the hippocampus and frontal cortex. Insulin-degrading enzyme (IDE; **A**-**B**), and nicastrin (**C**-**D**) (with representative blots, normalized to controls), as well as acetylcholine (**E**–**F**) and glutamic acid (**G**-**H**) concentrations in hippocampus (**A**, **C**, **E**, **G**) and cortex (**B**, **D**, **F**, **H**) tissues from rats fed control diet (**C**), fructose-rich diet (**F**) or fructose-rich diet and *S. clausii* spores administration. Reported are the mean values ± SEM of 6 different rats. * *p* < 0.05, ** *p* < 0.01, *** *p* < 0.001, **** *p* < 0.0001 compared to C rats; # *p* < 0.05, ### *p* < 0.001, #### *p* < 0.0001 compared to F rats (One way ANOVA followed by Bonferroni’s post-test)

Since excessive fructose intake and over activation of its metabolism has been recently suggested as a driver of Alzheimer’s disease (AD) (Johnson et al. 2020; Yan et al. 2023), we evaluated in the brain tissues of the experimental groups the levels of key markers of early AD disease, namely insulin-degrading enzyme (IDE), one of the key enzymes involved in Aβ degradation (Farris et al. 2003), and nicastrin, a component of γ-secretase complex. Interestingly, IDE was found reduced (Fig. 7A,B), while nicastrin was found increased in both hippocampus and cortex of fructose treated rats, compared to control ones (Fig. 7C,D). Notably, *S. clausii* spore administration was able to prevent these modifications (Fig. 7A-D).

Targeted mass spectrometry analysis highlighted quantitative differences in the amounts of the major neurotransmitters acetylcholine (ACh) and glutamic acid in hippocampus and frontal cortex of C, F and Sp174 groups. Of note, the concentration of ACh, one of the main neuromodulators essential for integrating learning and memory functions (Dannenberg et al. 2017), was significantly lower in hippocampus (Fig. 7E) and frontal cortex (Fig. 7F) of F rats compared to C ones. These results were in line with the observed impairment of memory function and the reduction of molecular key markers of synaptic plasticity reported in the previous paragraphs. *S. clausii* spore administration was effective in restoring ACh levels (Fig. 7E,F), thus preventing memory defects and synaptic transmission dysfunction. Further, higher amounts of glutamate were detected in the frontal cortex from fructose fed rats compared to control ones (Fig. 7H). *S. clausii* spore treatment was associated with a significant reduction of glutamic acid compared to fructose fed rats in both hippocampus and cortex tissues (Fig. 7G,H).

### Sp174 alleviates fructose-induced neuroinflammation

**Fig. 8:**
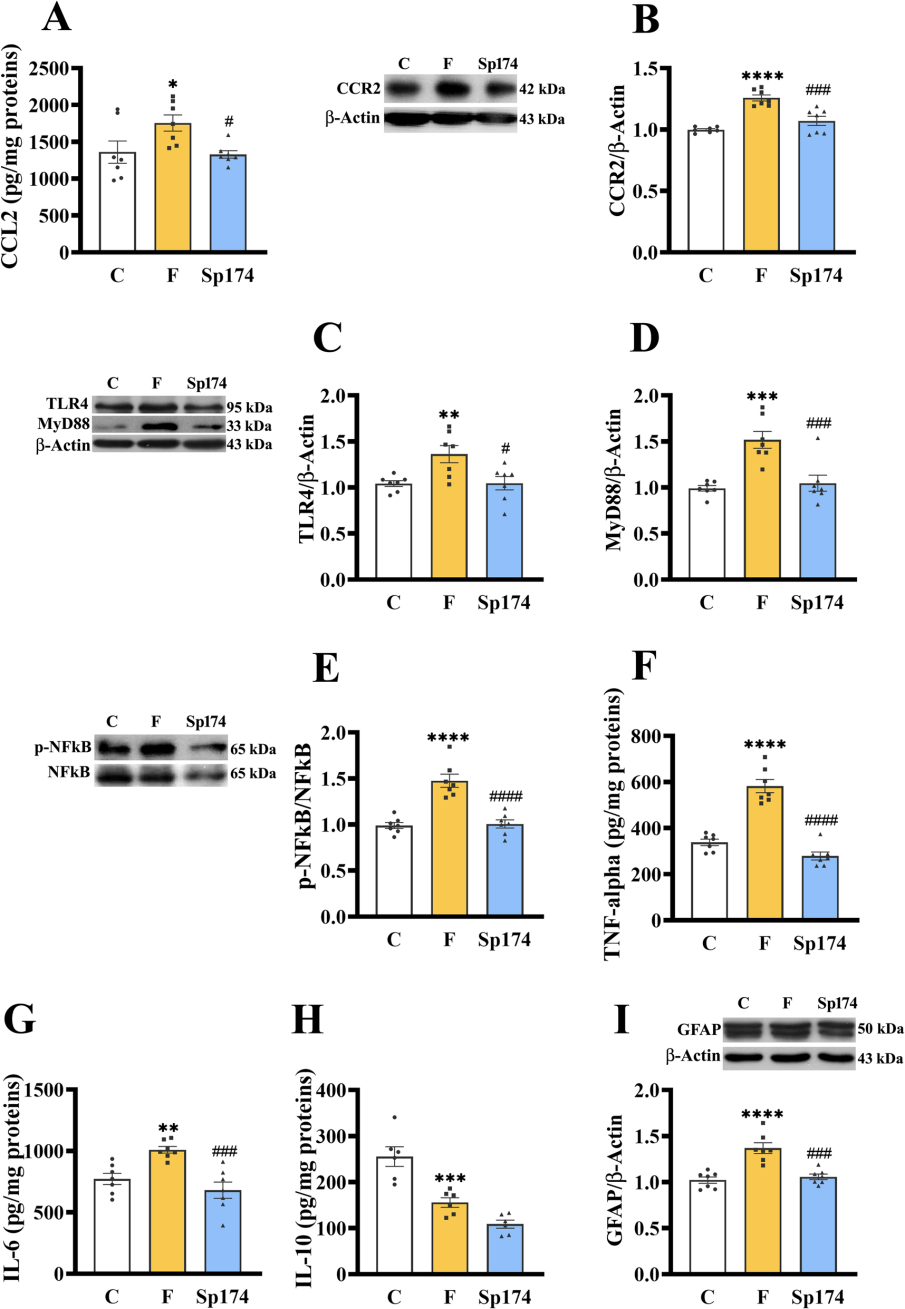
Markers of neuroinflammation in the hippocampus CCL2 (**A**), C–C chemokine receptor type 2 (CCR2) (**B**), Toll-like receptor 4 (TLR4) (**C**), and myeloid differentiation primary response 88 protein (MyD88) (**D**) amounts, phosphorylated NFkB/NFkB ratio (protein content with representative blots, normalized to controls) (**E**), tumor necrosis factor alpha (TNF-alpha) (**F**), interleukin 6 (IL-6) (**G**), and interleukin 10 (IL-10) (**H**) concentrations, and glial fibrillar acidic protein (GFAP) (**I**) amounts (with representative blots, normalized to controls) in the hippocampus from rats fed control diet (**C**), fructose-rich diet (**F**) or fructose-rich diet also including *S. clausii *spores (Sp174). Reported are the mean values ± SEM of 7 different rats. * *p* < 0.05, ** *p* < 0.01, *** *p* < 0.001, **** *p* < 0.0001 compared to C rats; # *p* < 0.05, ### *p* < 0.001, #### *p* < 0.0001 compared to F rats (One way ANOVA followed by Bonferroni’s post-test)

sBAs have been previously reported to play neuroprotective effect by inhibiting neuroinflammation (Monteiro-Cardoso et al. 2021; Darmanto et al. 2025). Therefore, we investigated whether the well-known neuroinflammatory impact of fructose-rich diet might be prevented by *S. clausii* spore administration. According to data showing that the activation of TGR5 signalling inhibits CCL2 secretion (Zhang et al. 2025a), increased amounts of both CCL2 (Fig. 8A) and its receptor CCR2 (Fig. 8B) were detected in fructose fed rats, compared to the control ones, with *S. clausii* spore treatment preventing this alteration.

**Fig. 9:**
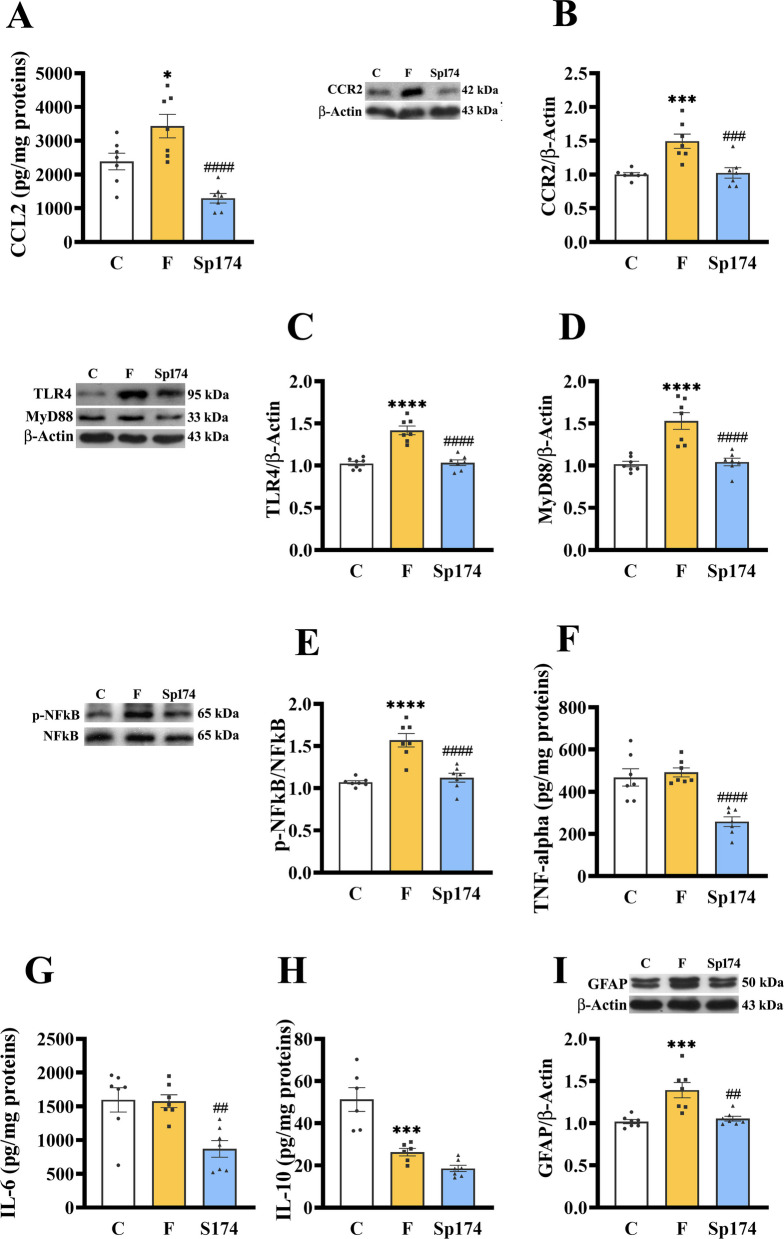
Markers of neuroinflammation in the frontal cortex. CCL2 (**A**), C–C chemokine receptor type 2 (CCR2) (**B**), Toll-like receptor 4 (TLR4) (**C**), and myeloid differentiation primary response 88 protein (MyD88) (**D**) amounts, phosphorylated NFkB/NFkB ratio (protein content with representative blots, normalized to controls) (**E**), tumor necrosis factor alpha (TNF-alpha) (**F**), interleukin 6 (IL-6) (**G**), and interleukin 10 (IL-10) (**H**) concentrations, and glial fibrillar acidic protein (GFAP) (**I**) amount (with representative blots, normalized to controls) in the frontal cortex from rats fed control diet (**C**), fructose-rich diet (**F**) or fructose-rich diet also including *S. clausii *spores (Sp174). Reported are the mean values ± SEM of 7 different rats. * *p* < 0.05, *** *p* < 0.001, **** *p* < 0.0001 compared to C rats; ## *p* < 0.01, ### *p* < 0.001, #### *p* < 0.0001 compared to F rats (One way ANOVA followed by Bonferroni’s post-test)

The degree of NFkB phosphorylation (used as marker of its activation), the level of the cytokines TNF-α and IL-6 as well as GFAP protein expression, known as marker of astrogliosis, were also evaluated. Increased amounts of Toll-like receptor 4 (TLR4) (Fig. 8C; Fig. 9C) and myeloid differentiation factor 88 (MyD88) (Fig. 8D; Fig. 9D), a downstream protein of TLR4 signaling pathway, as well as a higher degree of NFkB phosphorylation (Fig. 8E; Fig. 9E) were observed in both hippocampus and cortex of fructose fed rats, compared to control. The concentrations of TNF-α and IL-6 were higher in the hippocampus (Fig. 8F,G) but not in the cortex (Fig. 9F,G) of fructose fed animals. In line with the condition of neuroinflammation, higher GFAP levels were also observed in hippocampus (Fig. 8I) and frontal cortex (Fig. 9I) from fructose fed animals, compared to control ones. Notably, the fructose-associated activation of inflammatory pathways in the hippocampus (Fig. 8) and the frontal cortex (Fig. 9) were prevented by *S. clausii* spores administration. Interestingly, lower hippocampal and frontal cortex levels of IL-10 were found in fructose fed animals, compared to control (Fig. 8H; Fig. 9H); however, no significant differences were observed between the former rats and fructose fed animals also concomitantly supplemented with spores.

### Associations between DCA and molecular parameters in plasma and brain

**Fig. 10:**
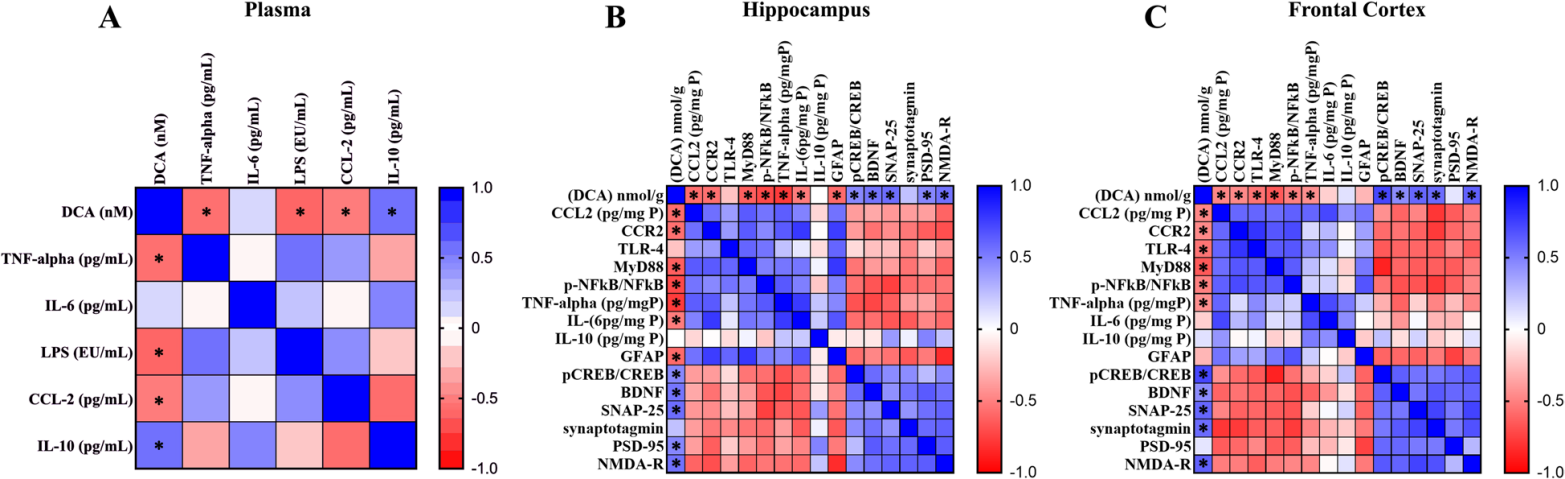
Associations between DCA and molecular parameters in plasma and brain. Heatmap summarizing the correlations between deoxycholic acid (DCA) and molecular markers in plasma (**A**), hippocampus (**B**), and frontal cortex (**C**). Spearman correlation coefficients (r) between the markers are plotted in the heatmap. Blue and red indicate positive and negative correlations, respectively. **A** DCA negative correlation with TNF-α (*p* = 0.013, r = −0.56), LPS (*p* =, 0.021 r = −0.595) and CCL2 (*p* = 0.032, r = −0.507); DCA positive correlation with IL-10 (*p* = 0.023, r = 0.554). **B** DCA negative correlation with CCL2, CCR2, MyD88, pNFkB/NFkB, TNF-alpha, IL-6 and GFAP (*p* = 0.01, r = −0.575; *p* = 0.01, r = −0.562; *p* = 0.008, r = −0.630; *p* = 0.0008, r = −0.717; *p* = 0.0005, r = −0.770; *p* = 0.037, r = −0.515; *p* = 0.015, r = −0.568, respectively); DCA positive correlation with pCREB/CREB, BDNF, SNAP-25, PSD-95 and NMDA-R (*p* = 0.045, r = 0.465; *p* = 0.027, r = 0.539; *p* = 0.013, r = 0.571; *p* = 0.041, r = 0.472; *p* = 0.023, r = 0.554, respectively). **C** DCA negative correlation with CCL2, CCR2, TLR4, MyD88, pNFkB/NFkB, TNF-alpha (*p* = 0.04, r = −0.475; *p* = 0.04, r = −0.463; *p* = 0.012, r = −0.549; *p* = 0.002, r = −0.653; *p* = 0.02, r = −0.501; *p* = 0.045, r = −0.442, respectively); DCA positive correlation with pCREB/CREB, BDNF, SNAP-25, synaptotagmin and NMDA-R (*p* = 0.0003, r = 0.72; *p* = 0.048, r = 0.448; *p* = 0.007, r = 0.596; *p* = 0.009, r = 0.579; *p* = 0.003, r = 619, respectively)

To gain further insights into the potential involvement of DCA changes in the observed inflammatory and synaptic markers alterations, correlation analyses were performed pooling data from all animals across treatments groups. Plasma concentration of DCA negatively correlated with TNF-α, LPS and CCL2 (*p* < 0.05), and positively with IL-10 (*p* < 0.05; Fig. 10A). In hippocampus, DCA was negatively correlated with CCL2, CCR2, MyD88, pNFkB/NFkB, TNF-alpha, IL-6 and GFAP (*p* < 0.05; Fig. 10B), and positively correlated with pCREB/CREB, BDNF, SNAP-25, PSD-95 and NMDA-R (*p* < 0.05; Fig. 10B). In frontal cortex DCA amount was negatively correlated with CCL2, CCR2, TLR4, MyD88, pNFkB/NFkB, TNF-alpha (*p* < 0.05; Fig. 10C), and positively correlated with pCREB/CREB, BDNF, SNAP-25, synaptotagmin and NMDA-R (*p* < 0.01; Fig. 10C).

### Intestinal microbiota composition

**Fig. 11:**
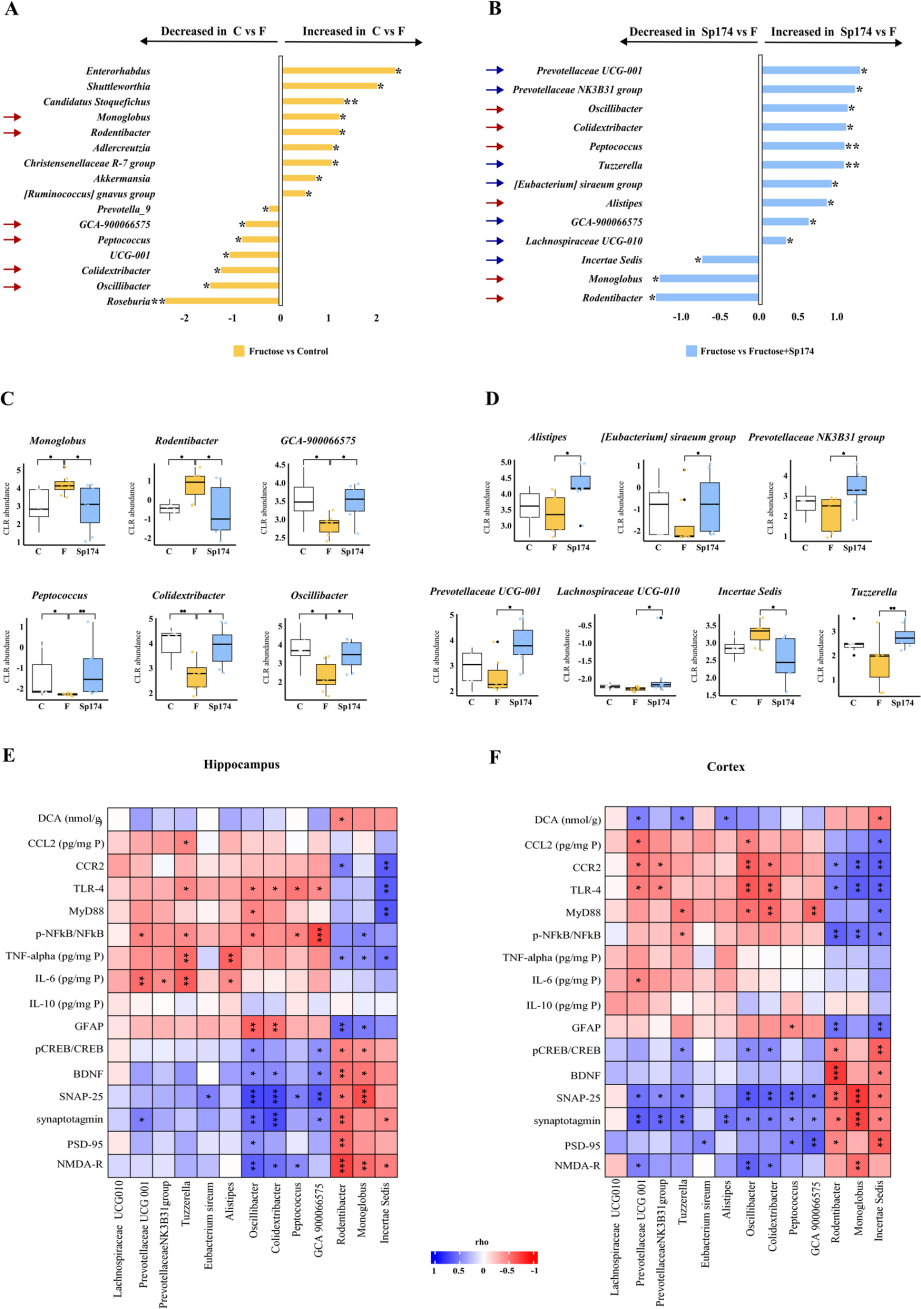
Differential abundance analysis of gut microbiota. Graphs display the genera with a statistically significant variation (*p* < 0.05) in the fructose fed group compared to the control (**A**) and in the Sp174 supplemented group compared to the F group (**B**). Taxa affected by the fructose-rich diet and then restored by the spores’ supplementation are indicated by red arrows and reported in panel **C**. Taxa affected by the spores supplementation are indicated by the blue arrows (panel B) and reported in panel **D**. Panels E and F show heatmaps of Spearman correlation analyses between the relative abundance of the selected bacterial taxa and brain molecular markers in the hippocampus (**E**) and cortex (**F**), respectively. Color intensity represents the correlation coefficient (rho), positive and negative correlations are represented in blue and red, respectively. Significant correlations are marked with asterisks (*p < 0.05; **p < 0.01; ***p < 0.001)

Since the composition of plasma BAs may be regulated by the action of intestinal bacteria, we analyzed the microbial population of the animal caecum to evaluate the effects of fructose and of the probiotic treatment in shaping the gut microbiota. The PCoA analysis based on Bray–Curtis’s distance and the analysis of two Alpha diversity indices (Shannon and Simpson) indicated that the microbial composition of the caecum of rats belonging to C, F and Sp174 groups was not strongly affected by either treatment (Supplementary Fig. 4). However, differentially abundant genera were identified using ALDEx2 with CLR transformation, Kruskal–Wallis’s test, and Dunn’s post-hoc comparisons (FDR < 0.05). Sixteen genera were significantly affected in the F vs. C group comparison, with 9 of them increased and 7 of them decreased by the fructose-rich diet (Fig. 11A). When F rats were compared with Sp174 counterparts, 13 genera were differentially represented, with 10 increased and 3 decreased by the *S. clausii* spore treatment (Fig. 11B). Six of the above-mentioned 13 genera (red arrows in Fig. 11B) were also altered in the F vs. C group comparison (red arrows in Fig. 11A); in Sp174 rats, their abundance was restored to levels similar to that of the control group (Fig. 11C). The other 7 genera altered by the spore treatment (blue arrows in Fig. 11B) were not affected by fructose, and therefore their modified abundance was considered as a diet-independent effect of the spore administration on the gut microbiota (Fig. 11D).

Despite the spores supplementation, we didn’t find an increase of the relative abundance of *Shouchella clausii* reads in the animals supplemented with SF174 spores with respect to the other groups. This result is in line with other reports (Filippidou et al. 2015; Browne et al. 2016) assessing the under-estimation of endospore-forming bacteria in environmental samples mainly due to the low efficiency of DNA extraction from bacterial spores.

To evaluate the presence of SF174, the same cecal samples used to extract total DNA for 16S sequencing, were used to analyze the presence of live SF174 spores by colony forming units (CFU) determination, taking advantage of the antibiotic resistances of SF174 (Saggese et al. 2022). Results of this experiment are reported in Table 1. As reported in the table, we detected SF174 CFU only in the group treated with the probiotic, with an average of 1.18 ± 1.1 per gram of cecal sample. Since similar CFUs were obtained also when ethanol was used to discriminate between ethanol-resistant spores and ethanol-sensitive vegetative cells present in the sample (Hong et al. 2009), we concluded that all the counted CFUs were due to SF174 spores present in the cecal samples (Table 1).

**Table 1:** Colony Forming Units (CFU) from caecal samples^a^

Group	Untreated samples	Ethanol-treated samples
C	-	-
F	-	-
Sp174	1.18 ± 1.1	1.21 ± 1.0

^a^Value are × 10^6^ CFU per gram

### Associations between microbial taxa and molecular parameters in plasma and brain

To explore the relationships between gut microbial composition and host metabolic, inflammatory and neurobiological parameters, we performed a correlation analysis among microbial abundances and inflammatory/molecular markers measured in plasma (Supplementary Fig. 5), hippocampus and frontal cortex (Fig. 11E and Fig. 11F, respectively).

The results indicate that the genera *Monoglobus* and *Rodentibacter*, both increased in F rats and restored by spores supplementation (Fig. 11C), were positively associated (blue boxes) with inflammatory markers in hippocampus and frontal cortex (including CCR2, TLR4, p-NFkB/NFkB, TNFα, GFAP; Fig. 11E,F). The abundance of the same taxa was inversely correlated with the abundance of molecules involved in synaptic plasticity (such as pCREB/CREB, BDNF, SNAP-25, synaptotagmin, NMDAR) in hippocampus and frontal cortex (Fig. 11E,F). The genera *Rodentibacter* and *Incerte sedis *were also negatively associated with the abundance of DCA in hippocampus and cortex, respectively, further supporting a potential role of the microbiota in modulating inflammation through the regulation of DCA levels.

Conversely, all the taxa increased in the Sp174 rats had an opposite trend, being positively associated with the abundance of molecules involved in brain functioning (Fig. 11E,F blue boxes) and negatively associated with most of the analyzed inflammatory markers (Fig. 11E,F red boxes). This applies to both the genera reduced by the fructose-rich diet and maintained at higher levels by the spores supplementation (including GCA-900066575, *Peptococcus, Colidextribacter, Oscillibacter,* Fig. 11C) and the genera specifically increased by the spores treatment independently from the diet (including *Alistipes, Eubacterium sireum, Prevotellaceae* UCG001 and NK3B31, *Lachnospiraceae* UCG010, Fig. 11D). Similar results have been obtained when considering the markers in the plasma (Supplementary Fig. 5).

## Discussion

The interrelation between fructose, gut microbiota and the concentration of plasma metabolites having microbial origin is largely unknown. In this view, we used an untargeted metabolomic approach to identify molecular classes differentially represented in the plasma of rats fed a fructose-rich diet compared to the control animals. According to combination of discriminant analysis and Molecular Network function, we focused on bile acids as a critical molecular class that can mediate the communication between the gut and the brain. Indeed, BAs, which are generated by hepatic enzymes and modified by intestinal bacteria, in addition to established effects on gut function (Yang et al. 2021) are also involved in inflammatory signalling; accordingly, they can be a part of the effects of microbiota on brain functions (Monteiro-Cardoso et al. 2021; Darmanto et al. 2025).

The depletion of sBAs in plasma, together with the increase of conjugated bile acids observed in rats fed fructose-rich diet, was in line with the disturbance of bile acid metabolism provoked by gut dysbiosis (Yang et al. 2021) and with the condition of fructose-induced leaky gut previously described in this experimental paradigm (Saggese et al. 2024). In particular, the increase of plasma GCA, as well as the decrease of DCA, well agreed with the increased levels of LPS, TNF-α, and CCL2 observed in fructose fed rats, while the concomitant administration of *S. clausii* spores prevented these alterations. This spore-dependent anti-inflammatory effect may be due to the concomitant observed increase of DCA, as this sBA was previously shown to suppress pro-inflammatory cytokine production from peripheral blood-derived macrophages, through the activation of the TGR5 receptor (Sinha et al. 2020), as well as to the decrease in GCA, which was already shown to be correlated with biomarkers of inflammation (Zhao et al. 2021). An additional effect of the spores was the significant increase in plasma levels of IL-10, one of the most relevant anti-inflammatory cytokines (Branchett et al. 2024), which might potentiate the protective effect played by DCA. The putative role of DCA in mediating spore-associated modulation of systemic inflammatory response is supported by the finding of a negative association between plasma levels of DCA and plasma amounts of TNF-α, LPS and CCL2, and a positive association with IL-10 levels.

As one of the main objectives of this study was to investigate whether the spores may prevent the fructose-induced memory dysfunction through the gut-brain axis, we evaluated the short-term memory by the NOR test, one of the most employed experimental approaches to examine working memory (Ghafarimoghadam et al. 2022). The discrimination index was significantly decreased in fructose fed rats, confirming an impairment of the short-term memory induced by the sugar. Interestingly, *S. clausii* spore supplementation in concomitance with fructose intake was effective in preventing memory dysfunction, since a higher value was measured in the Sp174 group compared to the F one.

The beneficial impact of the spores on cognition in fructose fed rats can arise from different pathways. Specifically, within sBAs showing a differential representation in C, F and Sp174 groups, DCA was intriguing as this metabolite might represent an understudied direct molecular link between the intestinal microbiome and the brain. The trend of hippocampus and frontal cortex DCA changes in the 3 groups of rats reflected those of the plasma. Although both systemic circulation and local synthesis in the brain contribute to the brain bile acid pool, secondary bile acids found in the brain are of intestinal origin, since they are exclusively generated by the gut bacterial enzymes (Monteiro‑Cardoso et al. 2021). This prompted us to further investigate its potential action in the brain. We therefore decided to deepen the molecular pathways driven by DCA in specific brain regions particularly relevant for the learning and memory, namely the hippocampus and the frontal cortex. In this context it should be mentioned that a central role in mediating BA effects is played by TGR5 receptor which has a higher binding affinity to secondary BAs (Ye et al. 2024). Specifically, TGR5 is known to trigger critical cascade that governs synaptic plasticity, gene expression, and memory formation (Monteiro-Cardoso et al. 2021; Darmanto et al. 2025). Indeed, TGR5 binds to DCA, activates CREB (Darmanto et al. 2025), whose phosphorylation initiates the synthesis of BDNF, a pivotal neurotrophin for cell survival, neurotransmitter modulation, and long-term synaptic plasticity (Kowiański et al. 2018). Our data show that the fructose intake induced a decrease in the levels of DCA, TGR5, pCREB, BDNF, NMDAR and key proteins involved in synaptic plasticity (SNAP-25, synaptotagmin, PSD95, Erk1/2 and Akt). All the above-reported markers were similarly affected in hippocampus and frontal cortex tissues. *S. clausii* spore supplementation prevented the fructose-driven reduction in DCA and TGR5 both in the hippocampus and the frontal cortex. The above variations are in line with the finding that an increase of sBAs stimulates TGR5 expression (Ye et al. 2024). The spore-associated increase in DCA and TGR5 prevented the fructose-induced decrease of hippocampal and frontal cortex levels of pCREB, BDNF, NMDAR, and downstream kinases of BDNF, namely Erk1/2 and Akt, mediating neuroprotective effect of neurotrophin and regulation of neuronal survival, synaptic plasticity, and neurogenesis (Schirò et al. 2022; Barde 2025). The beneficial activity of the probiotic spores was further corroborated by the increase of synaptotagmin I, a major calcium sensor for transmitter release (Courtney et al. 2019), SNAP-25, which plays a central role in synaptic vesicle exocytosis and neurotransmitter release (Zhang et al. 2025b), and PSD-95 that is pivotal for the protein scaffolding in excitatory neurons (Pinto et al. 2013). In addition, a positive association between DCA and pCREB/CREB, BDNF, the pre-synaptic protein SNAP-25 and NMDAR was observed in both hippocampus and frontal cortex, reinforcing the hypothesis of the central role played by DCA in modulating brain physiology.

The alterations in BDNF, NMDAR as well as synaptic players here reported reinforce the idea that fructose intake may represent an initiator of AD (Johnson et al. 2020, 2023; Yan et al. 2023). In line with this hypothesis, decreased levels of IDE and increased levels of nicastrin were found in fructose fed rats. Notably, *S. clausii* spore supplementation was able to totally prevent the changes of all above-mentioned markers representative of an “early AD-like” condition in F rats. Overall, we can speculate that the probiotic spore-induced TGR5 signalling pathway can contribute, via DCA, to prevent a fructose-driven early AD-like phenotype. Indeed, a previous study highlighted the neuroprotective role of TGR5 agonists in neurological disorders such as Alzheimer’s diseases (Ackerman and Gerhard 2016). The idea that bile acids might act as neuroactive molecules is further supported by studies showing that administration of BAs improved neuropathological phenotypes in animal models of AD (Bazzari et al. 2019; Monteiro-Cardoso et al. 2021).

Interestingly, the concentration of acetylcholine was significantly lower in both hippocampus and frontal cortex of F rats, compared to C ones, in agreement with the observed impairment of memory function in the former group. Notably, a diet-associated dysregulation of glutamic acid was also revealed in the frontal cortex, as an increased amount of this excitatory neurotransmitter was detected in fructose fed rats compared to control, with *S. clausii* spores being able to prevent the alteration. This change might be a compensative response to the fructose-induced reduction of the glutamate receptor NMDA and may be indicative of a compromission of the synaptic balance (Sood et al. 2021), in agreement with the alteration of key synaptic markers here evidenced in fructose fed rats. Glutamic acid increase might also depend on the condition of neuroinflammation detected in fructose fed rats, since inflammation has already been reported to impact glial glutamic acid regulation at the cellular, molecular, and metabolic level (Haroon et al. 2017).

The efficacy of *S. clausii* spores in preserving the hippocampus and frontal cortex tissues from the sugar diet-induced alterations may also depend on the probiotic-induced decrease in plasma circulating inflammatory mediators. We therefore studied the ability of these spores to prevent the onset of neuroinflammation, a main culprit for the cognitive dysfunction associated with unhealthy diets (Zhang et al. 2022). Indeed, lower amounts of GFAP, suggestive of a condition of astrogliosis, together with higher amounts of TLR4 and its downstream pathway effector Myd88, and NFkB activation were observed in the hippocampus and the frontal cortex of fructose fed rats. Interestingly, the levels of pro-inflammatory mediators TNF-α and IL-6, which are key contributors to the neuroinflammatory processes implicated in neurological disorders, such as AD (Ng et al. 2018), were increased in hippocampus tissues. These data support the hypothesis that *S. clausii* spores*,* through the increase of DCA and the expression of TGR5, drive molecular pathways that limit neuroinflammation. In our experimental model, according to the observed increase of DCA and TGR5 levels, we found that concomitant *S. clausii* spore administration consistently reduces neuroinflammation, compared to fructose feeding alone, as evidenced by reduced astrogliosis (lower GFAP), reduced activation of the NFkB in both hippocampus and frontal cortex, and decreased production of pro-inflammatory cytokines (TNF-α and IL-6) in the above-mentioned brain tissues. Notably, our results evidenced lower levels of both CCL2 and its receptor CCR2 in both hippocampus and cortex of Sp174 rats, in line with data evidencing that TGR5 stimulation mediates the inhibition of microglia activation by suppressing the neuron secretion of CCL2 (McMillin et al. 2015; Zhang et al. 2025a), thus playing a neuroprotective effect against the neuroinflammation associated with fructose consumption. The potential role of DCA in preventing diet-induced neuroinflammation is further supported by its negative correlation with both CCL2 and CCR2, as well as with several markers of inflammatory response activation, in hippocampus and frontal cortex. Intriguingly, the hippocampal and frontal cortex decrease in IL-10, which further corroborates the neuroinflammatory impact of the fructose feeding, was not prevented by *S. clausii* spore administration, differently from the upregulation evidenced in systemic plasma. This result can be indicative of the involvement of different cells and/or metabolites/signals, underlying the production of this anti-inflammatory cytokine in systemic plasma compared to the brain. In this context, it is worth mentioning that, although no data are available so far on the role of *S. clausii* in bile acids metabolism, its immunomodulatory activity has been widely demonstrated using animal models and cell lines (Ghelardi et al. 2022; Lashermes et al. 2025). *S. clausii* strains are known to exert anti-inflammatory effect through different mechanisms, such as modulation of pro- and anti-inflammatory cytokines, induction of nitric oxide production, decrease of immune cell infiltration, stimulation of mucus-secreting goblet cells, essentially depending on the causal agent of inflammatory condition (Urdaci et al. 2004; Di Caro et al. 2005; Paparo et al. 2020; Wong-Chew et al. 2022; Khokhlova et al. 2023; Saggese et al 2024; Vittoria et al. 2024; Zainab et al. 2024). Recently, a functional genomics investigation contributed to clarify its mechanism of action, identifying 23 coding sequences associated with NFkB modulation and six related to IL-10 modulation in four *S. clausii* strains (Lashermes et al. 2025).

Finally, since fructose diet is known to alter gut microbiota (Sindhunata et al. 2022), a comparative analysis was performed to assess whether the probiotic spore supplementation influenced the gut microbial composition. Consistently with previous reports (Sindhunata et al. 2022), we observed that the fructose-rich diet altered the composition of the gut microbiota affecting the relative abundance of 16 genera. The abundance of six of them (red arrows in Fig. 11A,B) was restored by spore supplementation to levels similar to those observed in control animals (Fig. 11C).

Among the genera reduced in F rats and rescued by *S. clausii* spores supplementation, there are *Oscillibacter*, *Peptococcus* and *Colidextribacter*. The genus *Oscillibacter* has been already reported to produce valeric acid that has an inhibitory effect on histone deacetylase isoforms implicated in metabolic and neurodegenerative diseases (Yuille et al. 2018). The genus *Peptococcus* is known to participate in BAs sulfation, a major metabolic pathway for detoxification and elimination of bile acids (Larabi et al. 2023), and its reduction has recently been associated with depression (Palepu et al. 2024). The increase of both genera in the Sp174 group was in line with the neuroprotective activity of *S. clausii* spores. Similarly, the genus *Colidextribacter* that has been positively correlated with the BAs metabolism (Li et al. 2024), was decreased by the fructose diet and restored by the spores supplementation, thus confirming the protective effect of *S. clausii* spores.

Other genera, such as *Monoglobus* and *Rodentibacter* resulted increased in the F rats and rescued to level similar to the control in the spore-supplemented animals. These findings corroborate our data on the beneficial effects of the microbial spores as these taxa are respectively involved in inflammation (Vaziri et al. 2013) and in cognitive dysfunction (Han et al. 2020).

Notably, our analysis also revealed that the abundance of seven genera that were not affected by the fructose diet compared to controls, was specifically enriched by the spore treatment (Fig. 11B, blue arrows, and Fig. 11D). Among these genera there are taxa involved in the conversion of BAs into sBAs. Bacteria belonging to the genus *Lachnospiraceae* (Lachnospiraceae UCG010) are examples of 7α-dehydroxylating Gram-positive bacteria that are responsible for the production of DCA (Kakiyama et al. 2013; Urdaneta and Casadesús, 2017). In addition, *Eubacterium siraeum* has been found positively correlated with fecal levels of several secondary BAs (Li et al. 2024), and bile salt hydrolase (BSH), 7α-dehydroxylase, and hydroxysteroid dehydrogenase activities have been described in *Eubacterium* genus (Song et al. 2019; Mukherjee et al. 2020; Cai et al. 2022). Hence, these microorganisms may have contributed to the DCA increase observed in the Sp-174 group. Other taxa are known for their neuroprotective activity. This is the case of bacteria belonging to the *Eubacterium sireum* group that, in line with the proposed neuroprotective role (Murru et al. 2021), have been previously found poorly represented in AD patients (Chen et al. 2022). Finally, the genus *Alistipes*, increased in the Sp174 group, is a relatively recent sub-branch genus of the *Bacteroidetes* phylum known to have anti-inflammatory properties (Lin et al. 2025) and potentially offering protection against colitis and autism spectrum disorder (Parker et al. 2021). The same genus has been found as the most prominent carrier of bile salt hydrolase genes (Shang et al. 2025) in a recent analysis of gut microbiomes from caprinae animals, supporting the hypothesis that its increased abundance might have contributed to the observed changes of sBA in the Sp174 group.

The involvement of these microbial taxa in the spore mediated beneficial effects is corroborated by a correlation analysis evidencing the relationships between gut microbiota composition and host inflammatory or neurobiological parameters. Our analysis shows that the taxa positively associated with inflammatory markers (including IL-6, TNF-α, CCL2, LPS) and negatively with key synaptic and neurotrophic markers (including BDNF, pCREB, PSD-95, SNAP25) were reduced in Sp174-treated animals. On the other hand, taxa negatively associated with inflammatory markers, were increased in Sp174-supplemented rats, suggesting a potential role of these genera in dampening systemic inflammation. Interestingly, the reported changes of cecal microbiota genera are different compared to those previously observed in fecal samples (Saggese et al. 2024). This is not surprising as taxonomic and functional differences between stool samples and cecal content have been previously reported (Tanca et al. 2017; Guo et al. 2019). These differences reflect the high complexity of gut microbiota, also in terms of diet induced variations of its composition, highlighting the importance of comprehensive investigations of microbiota of different gut regions, to get further insight into its influence on brain.

Together, these findings suggest that the probiotic spores supplementation induced changes in cecal microbial composition towards a healthier microbiota, reducing the abundance of taxa associated with inflammation and reduction of neuronal plasticity, while increasing the abundance of taxa linked to a reduced inflammatory tone and enhanced synaptic integrity.

**Fig. 12:**
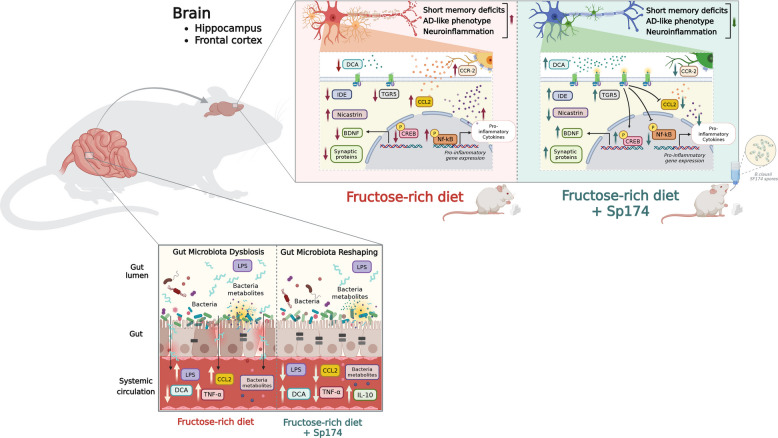
Fructose impact on brain health of rats and protective effect of the spores. Up arrows indicate increased parameters and down arrows indicate reduced parameters in F rats and/or Sp174 rats

From our results, we can outline a dual role of the *S. clausii* spores, one linked to their ability to prevent the deleterious effect of fructose feeding on the gut microbiota and the other one acting on modulation of gut microbes independently from the diet. These effects could partly be mediated by probiotic spore action on the levels of DCA and TGR5 downstream pathways, which are involved in the fructose impact on hippocampus and frontal cortex tissues (Fig. 12), thus providing a new insight into understanding the link between dietary fructose and the gut brain axis and contributing to prioritize future work in this research field.

## Supplementary Information

Additional file 1: Supplementary Table 1. Composition of experimental diets. Supplementary Table 2. NCBI Sequence Read Archive (SRA) accession numbers of data generated in this study (BioProject number PRJNA1291627). Supplementary Table 3. Dilutions of primary and secondary antibodies used for Western blotting. Supplementary Table 4: Bile acids reported in the analytical workflow. Compounds were detected and quantified in full scan negative ions mode [M-H]-; a normalized collision energy of 30% for structural confirmation combined with an experimental mass error below 2 ppm were used for calibration curves, quality controls and samples. Supplementary Fig. 1. Analyte responses in the plasma of the two groups fed fructose-rich diet (F, light blue points) and control diet (C, orange points) were analyzed through a principal component analysis (PCA) (Supplementary Fig. 1A and B). Samples 2D distribution explained an overall 46.3% of the total variation in negative ions (panel A) and 34.9% of the total variation in positive ions (panel B). Discriminant analysis through volcano plots (Supplementary Fig. 1C and D) depicting the compound area counts in the two dietary regimens through a 1 vs. 1 comparison to highlight differences in plasma metabolites by separating negative (panel C) and positive ion mode (panel D). False discovery rate correction was applied through Benjamini-Hochberg’s post-hoc analysis, and a significance level of 0.05 was used. Metabolites significantly associated with each diet were marked in dark red or dark green; over-represented analytes with Log2 mean ratio fold change higher than 1 and a Log10 for p-value higher than 1.3 were dark red-labelled, while down-represented analytes with Log2 mean ratio fold change lower than -1 and a Log10 for p-value higher than 1.3 were dark green-labelled; GCA: glycocholic acid. Molecular Network layout related to bile acids group in negative ion mode (Supplementary Fig. 1E); the procedure involved the connection of tandem mass spectra according to the fragmentation similarities. Light green points include all the compounds for which the processing workflow identified fragmentation spectra with mzCloud or internal library; dark green points report all the compounds matching with theoretical isotopic pattern distribution, mass error and fragmentation spectra and with a mass list search or a ChemSpider search; blue points report the compounds for which the processing workflow determined only the molecular formula. Orange lines include all the bio-transformations considered in the Molecular Network. Tauroursodeoxycholic acid (TUDCA), taurochenodeoxycholic acid (TCDCA) and taurodeoxycholic acid (TDCA), glycocholic acid (GCA), cholic acid (CA), deoxycholic acid (DCA). Compounds that were not unequivocally identified in the subsequent targeted analysis were only outlined by using the molecular weight values. Supplementary Fig. 2. Plasma concentration of Bile acids (BAs). Concentration of cholic acid (A), β-muricholic acid (β-MCA; B), taurodeoxycholic acid (TDCA; C), taurochenodeoxycholic acid (TCDCA; D), tauroursodeoxycholic acid (TUDCA; E) in plasma samples from rats fed control diet (C), fructose-rich diet (F) or fructose-rich diet and *Shouchella clausii* spores (Sp174). Reported are the mean values ± SEM of 6 different rats. Supplementary Fig. 3. Bile acids in hippocampus and frontal cortex. Taurocholic acid (TCA; A and B); tauromuricholic acid (tauro-MCA; C and D), and cholic acid (E and F) in samples of hippocampus (A, C and E) and frontal cortex (B, D and F) from rats fed control diet (C), fructose-rich diet (F) or fructose-rich diet and *Shouchella clausii* spores (Sp174). Reported are the mean values ± SEM of 6 different rats. Supplementary Fig. 4. Analysis of alpha and beta diversity. The microbial taxa diversity based on Shannon (A) and Simpson (B) indices. Beta-diversity was analysed using Bray–Curtis distances (C). Plots were generated based on the weighted UniFrac distance matrix. Different experimental groups are indicated by various colors, as reported. Supplementary Fig. 5. Spearman correlation heatmap between gut microbial genera and plasma inflammatory markers. Heatmap showing pairwise Spearman correlation coefficients (ρ) between the relative abundances of selected gut microbial genera and plasma concentrations of DCA, IL-6, TNF-α, CCL2, IL-10 and LPS. Blue indicates positive correlations, red indicates negative correlations, and white represents values close to zero. Asterisks indicate statistical significance (*p< 0.05; **p < 0.01; ***p < 0.001).

Additional file 2.

## Data Availability

The data used and/or analyzed in this study are available from the corresponding author upon reasonable request.

## Abbreviations

BA: Bile acid
sBAs: Secondary bile acids
HFCS: High-fructose corn syrup
Sp174: Shouchella clausii spores
CFU: Colony forming units
PCoA: Principal Coordinates Analysis
TNF-α: Tumor necrosis factor-alpha
IL-6: Interleukin-6
CCL2: C-C motif chemokine ligand 2
IL-10: Interleukin-10
LPS: Lipopolysaccharide
PSD-95: Post-synaptic density protein 95
SNAP25: Synaptosome-associated protein
BDNF: Brain derived neurotrophic factor
CREB: CAMP response element binding protein
TLR4: Toll-like receptor-4
MyD88: Myeloid differentiation factor 88
NFkB: Nuclear factor kappa-light-chain-enhancer of activated B cells
GFAP: Glial fibrillary acidic protein
TGR5: Takeda G protein-coupled receptor 5/Gpbar1
Akt: Protein kinase B
Erk1/2: Extracellular signal-regulated kinase 1/2
NMDAR: *N*-methyl-D-aspartic acid receptor
CCR2: C-C chemokine receptor type 2
IDE: Insulin-degrading enzyme
GAR-HRP: Goat anti-rabbit horseradish peroxidase-conjugated IgG
GAM-HRP: Goat anti-mouse horseradish peroxidase-conjugated IgG
NOR: Novel object recognition
DCA: Deoxycholic acid
7-KDCA: 7-Ketodeoxycholic acid
ω-MCA: ω-Muricholic acid
CA: Cholic acid
β-MCA: β-Muricholic acid
TDCA: Taurodeoxycholic acid
TCDCA: Taurochenodeoxycholic acid
TUDCA: Tauroursodeoxycholic acid
TCA: Taurocholic acid
TMCA: Tauromuricholic acid
AD: Alzheimer’s disease
GCA: Glycocholic acid
GHCA: Glycohyocholic acid
TMCA: Tauromuricholic acid
ACh: Acetylcholine
