# Supplementary material for: Gut-brain axis: beneficial impact of Shouchella clausii spores on fructose induced dysfunction is associated with modulation of the deoxycholic acid – TGR5 pathway
Source: Mol Med. 2026 May 9;32:101. doi: 10.1186/s10020-026-01479-4 (PMC13326143; doi:10.1186/s10020-026-01479-4)
Supplement: Supplementary file 1 — Additional file 1: Supplementary Table 1. Composition of experimental diets. Supplementary Table 2. NCBI Sequence Read Archive (SRA) accession numbers of data generated in this study (BioProject number PRJNA1291627). Supplementary Table 3. Dilutions of primary and secondary antibodies used for Western blotting. Supplementary Table 4: Bile acids reported in the analytical workflow. Compounds were detected and quantified in full scan negative ions mode [M-H]-; a normalized collision energy of 30% for structural confirmation combined with an experimental mass error below 2 ppm were used for calibration curves, quality controls and samples. Supplementary Fig. 1. Analyte responses in the plasma of the two groups fed fructose-rich diet (F, light blue points) and control diet (C, orange points) were analyzed through a principal component analysis (PCA) (Supplementary Fig. 1A and B). Samples 2D distribution explained an overall 46.3% of the total variation in negative ions (panel A) and 34.9% of the total variation in positive ions (panel B). Discriminant analysis through volcano plots (Supplementary Fig. 1C and D) depicting the compound area counts in the two dietary regimens through a 1 vs. 1 comparison to highlight differences in plasma metabolites by separating negative (panel C) and positive ion mode (panel D). False discovery rate correction was applied through Benjamini-Hochberg’s post-hoc analysis, and a significance level of 0.05 was used. Metabolites significantly associated with each diet were marked in dark red or dark green; over-represented analytes with Log2 mean ratio fold change higher than 1 and a Log10 for p-value higher than 1.3 were dark red-labelled, while down-represented analytes with Log2 mean ratio fold change lower than -1 and a Log10 for p-value higher than 1.3 were dark green-labelled; GCA: glycocholic acid. Molecular Network layout related to bile acids group in negative ion mode (Supplementary Fig. 1E); the procedure involved the connection of [file 10020_2026_1479_MOESM1_ESM.docx]

**Gut-Brain Axis: beneficial impact of *Shouchella clausii* spores on fructose induced dysfunction is associated with modulation of the deoxycholic acid – TGR5 pathway**

Maria Stefania Spagnuolo ^1#^, Natasha Petecca ^2#^, Francesca De Palma ^2^, Antonio Dario Troise ^1^, Angela Di Porzio ^2^, Valentina Barrella ^2^, Anella Saggese ^2^, Sabrina De Pascale ^1^, Marina De Stefano ^2^, Andrea Scaloni ^1^, Loredana Baccigalupi ^3,4,5^, Ezio Ricca ^2^, Susanna Iossa ^2,4,5^, Arianna Mazzoli ^2*^, Luisa Cigliano ^2,5*^

^1^ Institute for the Animal Production System in the Mediterranean Environment, National Research Council, P.le Enrico Fermi 1, 80055 Portici, Italy.

^2^ Department of Biology, University of Naples Federico II, Complesso Universitario Monte S. Angelo, Edificio 7, Via Cintia, 80126 Naples, Italy.

^3^ Department of Molecular Medicine and Medical Biotechnology, University of Naples Federico II, Via Pansini 5, 80100, Naples, Italy.

^4^ NBFC, National Biodiversity Future Center, 90133 Palermo, Italy.

^5^ Task Force on Microbiome Studies, University of Naples Federico II, 80126 Naples, Italy.

^#^ These authors equally contributed to the work.

*** Correspondence**:

Prof. Luisa Cigliano, Department of Biology, University of Naples Federico II, Complesso Universitario Monte S. Angelo, Edificio 7, Via Cintia, I-80126 Napoli, Italy.

E-mail: luisa.cigliano@unina.it, phone number: +39-081-2535244; ORCID <https://orcid.org/0000-0002-5491-9659>.

Dr. Arianna Mazzoli, Department of Biology, University of Naples Federico II, Complesso Universitario Monte S. Angelo, Edificio 7, Via Cintia, I-80126 Napoli, Italy.

[arianna.mazzoli@unina.it](mailto:arianna.mazzoli@unina.it); phone number: +39-081-081679165; ORCID [https://orcid.org/0000-0003-4096-443X](https://www.scopus.com/redirect.uri?url=https://orcid.org/0000-0003-4096-443X&authorId=55749073900&origin=AuthorProfile&orcId=0000-0003-4096-443X&category=orcidLink).

**Supplementary Information**

**Supplementary Table 1.** Composition of experimental diets.

| **Component (g/100g)** **Control diet** **Fructose diet** |
| --- |

Standard Chow^a^ 50.5 50.5

Sunflower Oil 1.5 1.5

Casein 9.2 9.2

Alphacel 9.8 9.8

Starch 20.4 ---

Fructose --- 20.4

Water 6.4 6.4

AIN-76 mineral mix 1.6 1.6

AIN-76 vitamin mix 0.4 0.4

Choline 0.1 0.1

Methionine 0.1 0.1

Gross Energy Density (kJ/g) 17.2 17.2

Metabolisable Energy Density (kJ/g) ^b^ 11.1 11.1

Proteins (% Metabolisable energy) 29.0 29.0

Lipids (% Metabolisable energy) 10.6 10.6

Carbohydrates (% Metabolisable energy) 60.4 60.4

Of which:

Fructose --- 30.0

Starch 52.8 22.8

Sugars 7.6 7.6

| ^a^ Mucedola 4RF21; Italy ^b^Estimated by computation using values (kJ/g) for energy content as follows: proteins 16.736, lipids 37.656 e carbohydrates 16.736 |
| --- |

**Supplementary Table 2.** NCBI Sequence Read Archive (SRA) accession numbers of data generated in this study (BioProject number [PRJNA1291627](https://www.ncbi.nlm.nih.gov/bioproject/PRJNA1291627)).

| **Sample name** | **Accession number** |
| --- | --- |
| C1 | SAMN49972235 |
| C2 | SAMN49972237 |
| C3 | SAMN49972239 |
| C4 | SAMN49972241 |
| C5 | SAMN49972243 |
| C6 | SAMN49972245 |
| C7 | SAMN49972247 |
| C8 | SAMN49972249 |
| F1 | SAMN49972251 |
| F2 | SAMN49972253 |
| F3 | SAMN49972255 |
| F4 | SAMN49972257 |
| F5 | SAMN49972259 |
| F6 | SAMN49972261 |
| F7 | SAMN49972263 |
| F8 | SAMN49972265 |
| Sf174_1 | SAMN49972267 |
| Sf174_2 | SAMN49972269 |
| Sf174_3 | SAMN49972271 |
| Sf174_4 | SAMN49972273 |
| Sf174_5 | SAMN49972275 |
| Sf174_6 | SAMN49972277 |
| Sf174_7 | SAMN49972279 |
| Sf174_8 | SAMN49972281 |

**Supplementary Table 3.** Dilutions of primary and secondary antibodies used for Western blotting.

|  | Primary Antibody | Secondary Antibody |
| --- | --- | --- |
| TLR4 | Sigma-Aldrich, 1:500 **^a^** (cat PRS3141) | GAR-HRP IgG, 1:150,000 **^a^** |
| MyD88 | Cell Signalling Technology; 1:1,000 **^a^** (cat 4283) | GAR-HRP IgG, 1:70,000 **^a^** |
| p-NFkB | Cell Signalling Technology; 1:500 **^a^** (cat 3033) | GAR-HRP IgG; 1:20,000 **^a^** |
| NFkB | Cell Signalling Technology; 1:1000 **^a^** (cat 8242) | GAR-HRP IgG; 1:30,000 **^a^** |
| CCR2 | Cell Signalling Technology; 1:1,000 **^a^** (cat 12199) | GAR-HRP IgG, 1:30,000 **^a^** |
| GFAP | Cell Signalling Technology; 1:1,000 **^b^** (cat 12389) | GAR-HRP IgG, 1:350,000 **^b^** |
| TGR5 | Elabscience; 1:500 ^a^ (cat E-AB-93270) | GAR-HRP IgG, 1:40,000 **^a^** |
| IDE | Abcam, Cambridge; 1:1000 **^a^** (ab32216) | GAr-HRP IgG; 1:90,000 **^b^** |
| Nicastrin | Santa Cruz Biotechnology; 1:500 **^a^** (cat sc-376513) | GAM-HRP IgG, 1:10,000 **^b^** |
| p-CREB | Cell Signalling Technology; 1:500 **^a^** (cat 9198) | GAR-HRP IgG, 1:20,000 **^a^** |
| CREB | Cell Signalling Technology; 1:1000 **^a^** (cat 9197) | GAR-HRP IgG, 1:40,000 **^a^** |
| BDNF | Abcam, Cambridge (EPR1292); 1:2000 **^a^** (cat ab108319) | GAR-HRP IgG, 1:150,000 **^a^** |
| p-Akt | Cell Signalling Technology; 1:1,000 **^a^** (cat 9271) | GAR-HRP IgG, 1:50,000 **^a^** |
| Akt | Cell Signalling Technology; 1:1000 **^a^** (cat 9272) | GAR-HRP IgG, 1:170,000 **^a^** |
| p-Erk1/2 | Cell Signalling Technology; 1:1,000 **^a^** (cat 9101) | GAR-HRP IgG, 1:120,000 **^a^** |
| Erk1/2 | Cell Signalling Technology; 1:1,000 **^a^** (cat 9102) | GAR-HRP IgG; 1:250,000 **^a^** |
| SNAP-25 | Cell Signalling Technology; 1:1000 ^a^ (cat 5308) | GAR-HRP IgG; 1:125,000 **^b^** |
| Synaptotagmin | Cell Signalling Technology; 1:1000 **^a^** (cat 14558) | GAR-HRP IgG; :350,000 **^b^** |
| PSD-95 | Cell Signalling Technology; 1:1000 **^a^** (cat 2057) | GAR-HRP IgG; 1:40,000 **^b^** |
| NMDAε2 | Santa Cruz Biotechnology; 1:500 **^a^** (cat sc-365597) | GAM-HRP IgG; 1:25,000 **^a^** |
| β -Actin | Sigma-Aldrich; 1:1000 **^a^** (cat A2228) | GAM-HRP IgG; 1:300,000 **^b^** |

GAR-HRP: Goat anti-rabbit horseradish peroxidase-conjugated IgG (Immunoreagents, Raleigh, NC, USA, cat GtxRb-003-DHRPX).

GAM-HRP: Goat anti-mouse horseradish peroxidase-conjugated IgG (Immunoreagents, Raleigh, NC, USA, cat GtxMu-003-DHRPX).

T-TBS: 130 mM NaCl, 20 mM Tris-HCl, 0.05% Tween, pH 7.4.

^a^ T-TBS containing 2% w/v BSA; ^b^ T-TBS containing 1% v/v non-fat milk.

**Supplementary Table 4.** Bile acids reported in the analytical workflow. Compounds were detected and quantified in full scan negative ions mode [M-H]^-^; a normalized collision energy of 30 for structural confirmation combined with an experimental mass error below 2 ppm were used for calibration curves, quality controls and samples.

| **Compounds** | **Formula** | **RT Time (min)** | ***m/z*** |
| --- | --- | --- | --- |
| cholic acid (CA) | C_24_H_40_O_5_ | 6.1 | 407.2803 |
| chenodeoxycholic acid (CDCA) | C_24_H_40_O_4_ | 7.9 | 391.2854 |
| α-muricholic acid (α-MCA) | C_24_H_40_O_5_ | 5.5 | 407.2803 |
| β-muricholic acid (β-MCA) | C_24_H_40_O_5_ | 5.7 | 407.2803 |
| dehydrolithocholic acid | C_24_H_38_O_3_ | 11.6 | 373.2748 |
| ω-muricholic acid (ω-MCA) | C_24_H_40_O_5_ | 5.9 | 407.2803 |
| deoxycholic acid (DCA) | C_24_H_40_O_4_ | 8 | 391.2854 |
| lithocholic acid (LCA) | C_24_H_40_O_3_ | 11 | 375.2905 |
| ursodeoxycholic acid (UDCA) | C_24_H_40_O_4_ | 6.4 | 391.2854 |
| dehydrolithocholic acid (DHCA) | C_24_H_38_O_3_ | 11.6 | 373.2748 |
| 7-ketodeoxycholic acid (7-KDCA) | C_24_H_38_O_5_ | 5.5 | 405.2646 |
| 12-ketochenodeoxycholic acid (12-KCDCA) | C_24_H_38_O_5_ | 5.7 | 405.2646 |
| glycocholic acid (GCA) | C_26_H_43_NO_6_ | 5.4 | 464.3018 |
| glycochenodeoxycholic acid (GCDCA) | C_26_H_43_NO_5_ | 6.3 | 448.3068 |
| glycodeoxycholic acid (GDCA) | C_26_H_43_NO_5_ | 6.5 | 448.3068 |
| glycolithocholic acid (GLCA) | C_26_H_43_NO_4_ | 8.6 | 432.3119 |
| glycoursodeoxycholic acid (GUDCA) | C_26_H_43_NO_5_ | 5.5 | 448.3068 |
| glycohyocholic acid (GHCA) | C_26_H_43_NO_6_ | 6 | 464.3018 |
| taurocholic acid (TCA) | C_26_H_45_NO_7_S | 6.6 | 514.2844 |
| taurochenodeoxycholic acid (TCDCA) | C_26_H_45_NO_6_S | 8.2 | 498.2895 |
| taurodeoxycholic acid (TDCA) | C_26_H_45_NO_6_S | 8.7 | 498.2895 |
| tauroursodeoxycholic acid (TUDCA) | C_26_H_45_NO_6_S | 6.7 | 498.2895 |
| tauromuricholic acid (TMCA) | C_26_H_45_NO_7_S | 5.7 | 514.2844 |
| taurolithocholic acid (TLCA) | C_26_H_45_NO_5_S | 12.3 | 482.2946 |
| murideoxycholic acid | C_24_H_40_O_4_ | 6.2 | 391.2854 |
| hyodeoxycholic acid | C_24_H_40_O_4_ | 6.4 | 391.2854 |
| ursocholic acid (UCA) | C_24_H_40_O_5_ | 5.2 | 407.2803 |
| cholic acid-d4 | C_24_H_36_D_4_O_5_ | 6.1 | 411.3054 |
| taurocholic acid-d4 | C_26_H_41_D_4_NO_7_S | 6.6 | 518.3095 |
| glycocholic acid -d4 | C_26_H_39_D_4_NO_6_ | 5.4 | 468.3269 |
| deoxycholic acid-d4 | C_24_H_36_D_4_O_4_ | 8 | 395.3105 |
| taurodeoxycholic acid-d4 | C_26_H_41_D_4_NO_6_S | 8.7 | 502.3146 |
| glycodeoxycholic acid-d4 | C_26_H_39_D_4_NO_5_ | 6.5 | 452.3320 |
| chenodeoxycholic acid-d4 | C_24_H_36_D_4_O_4_ | 7.9 | 395.3105 |
| taurochenodeoxycholic acid-d4 | C_26_H_41_D_4_NO_6_S | 8.2 | 502.3146 |
| glycochenodeoxycholic acid-d4 | C_26_H_39_D_4_NO_5_ | 6.3 | 452.3320 |
| tauroursodeoxycholic acid-d4 | C_26_H_41_D_4_NO_6_S | 6.7 | 502.3146 |
| glycoursodeoxycholic acid-d4 | C_26_H_39_D_4_NO_5_ | 5.5 | 452.3320 |
| lithocholic acid-d4 | C_24_H_36_D_4_O_3_ | 11 | 379.3156 |
| taurolithocholic acid-d4 | C_26_H_41_D_4_NO_5_S | 12.3 | 486.3197 |
| glycolithocholic acid-d4 | C_26_H_39_D_4_NO_4_ | 8.6 | 436.3370 |
| ursodeoxycholic acid-d4 | C_24_H_36_D_4_O_4_ | 6.4 | 395.3105 |


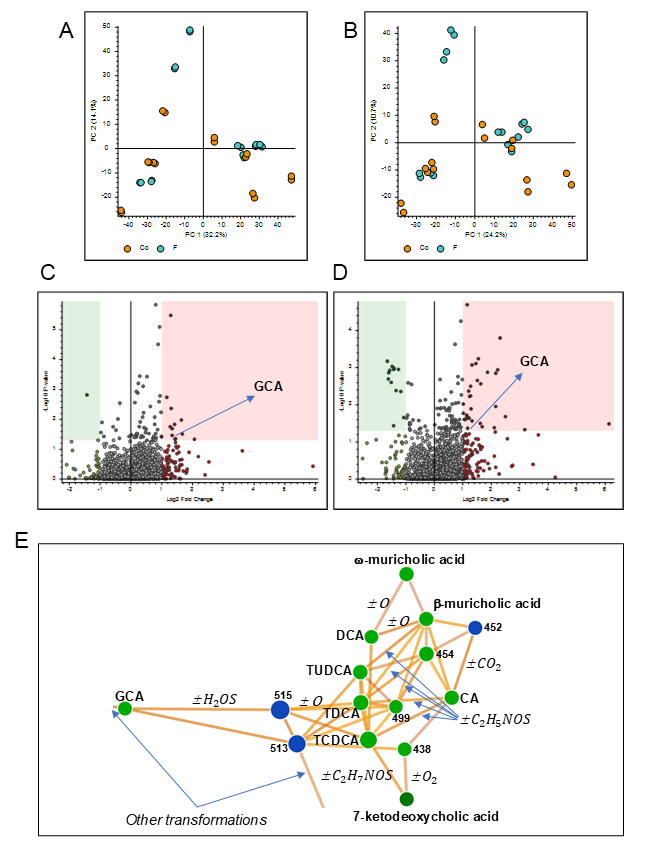


**Supplementary Fig. 1. Untargeted metabolomic analysis on plasma samples from rats fed a fructose-rich diet or a control diet.** Analyte responses in the plasma of the two groups fed fructose-rich diet (F, light blue points) and control diet (C, orange points) were analyzed through a principal component analysis (PCA) (Supplementary Fig. 1A and B). Samples 2D distribution explained an overall 46.3% of the total variation in negative ions (panel A) and 34.9% of the total variation in positive ions (panel B). Discriminant analysis through volcano plots (Supplementary Fig. 1C and D) depicting the compound area counts in the two dietary regimens through a 1 vs. 1 comparison to highlight differences in plasma metabolites by separating negative (panel C) and positive ion mode (panel D). False discovery rate correction was applied through Benjamini-Hochberg’s post-hoc analysis, and a significance level of 0.05 was used. Metabolites significantly associated with each diet were marked in dark red or dark green; over-represented analytes with Log2 mean ratio fold change higher than 1 and a Log10 for p-value higher than 1.3 were dark red-labelled, while down-represented analytes with Log2 mean ratio fold change lower than -1 and a Log10 for p-value higher than 1.3 were dark green-labelled; GCA: glycocholic acid. Molecular Network layout related to bile acids group in negative ion mode (Supplementary Fig. 1E); the procedure involved the connection of mass spectra according to the fragmentation similarities [1]. Light green points include all the compounds for which the processing workflow identified fragmentation spectra with mzCloud or internal library; dark green points report all the compounds matching with theoretical isotopic pattern distribution, mass error and fragmentation spectra and with a mass list search or a ChemSpider search; blue points report the compounds for which the processing workflow determined only the molecular formula. Orange lines include all the bio-transformations considered in the Molecular Network. Tauroursodeoxycholic acid (TUDCA), taurochenodeoxycholic acid (TCDCA) and taurodeoxycholic acid (TDCA), glycocholic acid (GCA), cholic acid (CA), deoxycholic acid (DCA). Compounds that were not unequivocally identified in the subsequent targeted analysis were only outlined by using the molecular weight values.


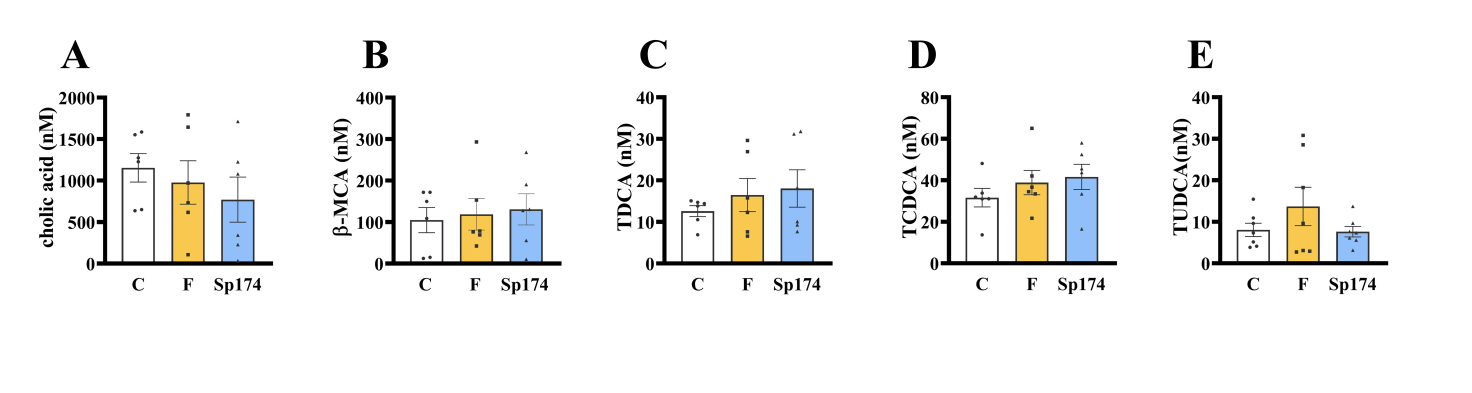


**Supplementary Fig. 2. Plasma concentration of Bile acids (BAs)**

Concentration of cholic acid (A), β-muricholic acid (β-MCA; B), taurodeoxycholic acid (TDCA; C), taurochenodeoxycholic acid (TCDCA; D), tauroursodeoxycholic acid (TUDCA; E) in plasma samples from rats fed control diet (C), fructose-rich diet (F) or fructose-rich diet and *Shouchella clausii* spores (Sp174). Reported are the mean values ± SEM of 6 different rats.


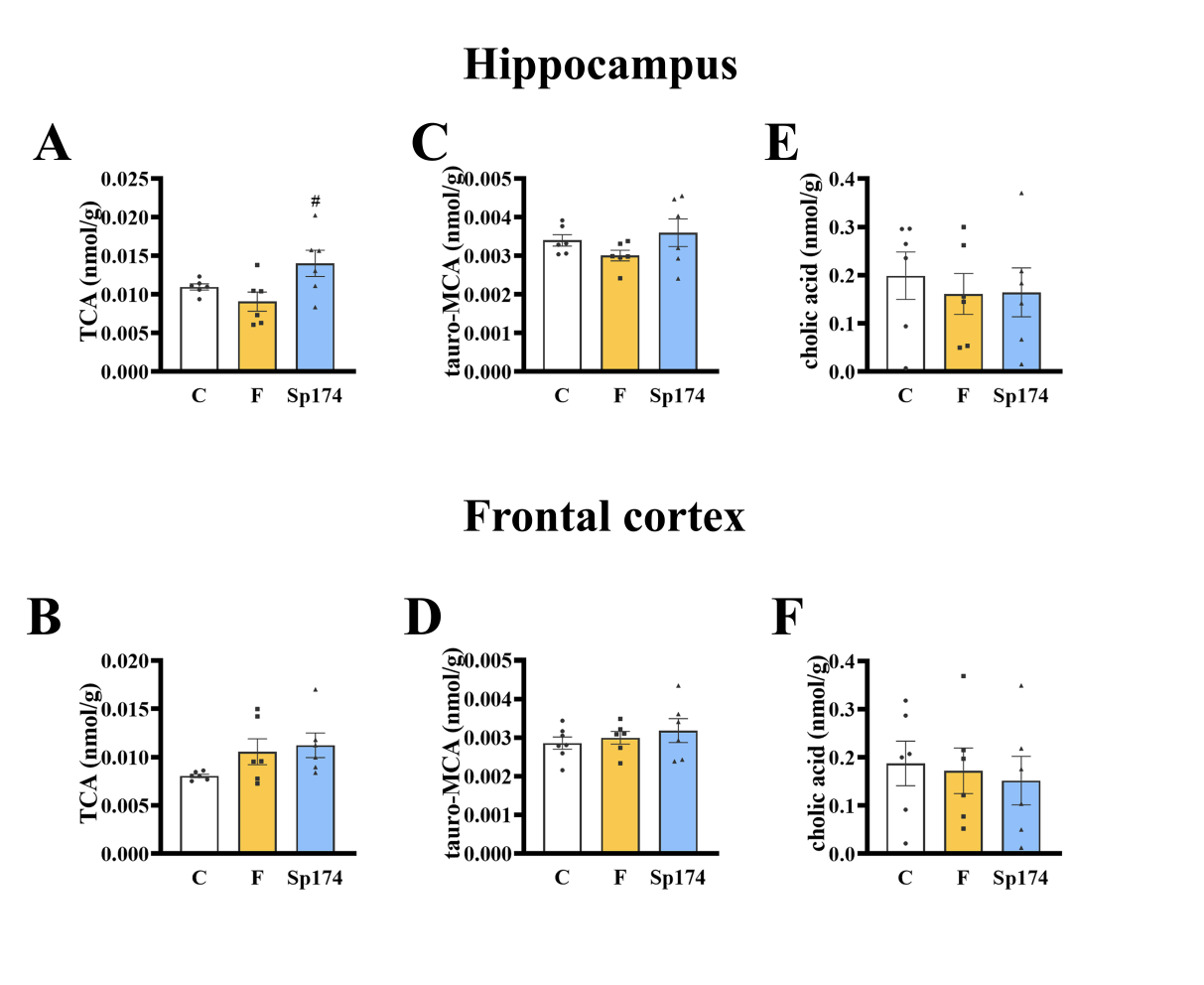


**Supplementary Fig. 3. Bile acids in hippocampus and frontal cortex**

Taurocholic acid (TCA; A and B); tauromuricholic acid (tauro-MCA; C and D), and cholic acid (E and F) in samples of hippocampus (A, C and E) and frontal cortex (B, D and F) from rats fed control diet (C), fructose-rich diet (F) or fructose-rich diet and *Shouchella clausii* spores (Sp174). Reported are the mean values ± SEM of 6 different rats.

**
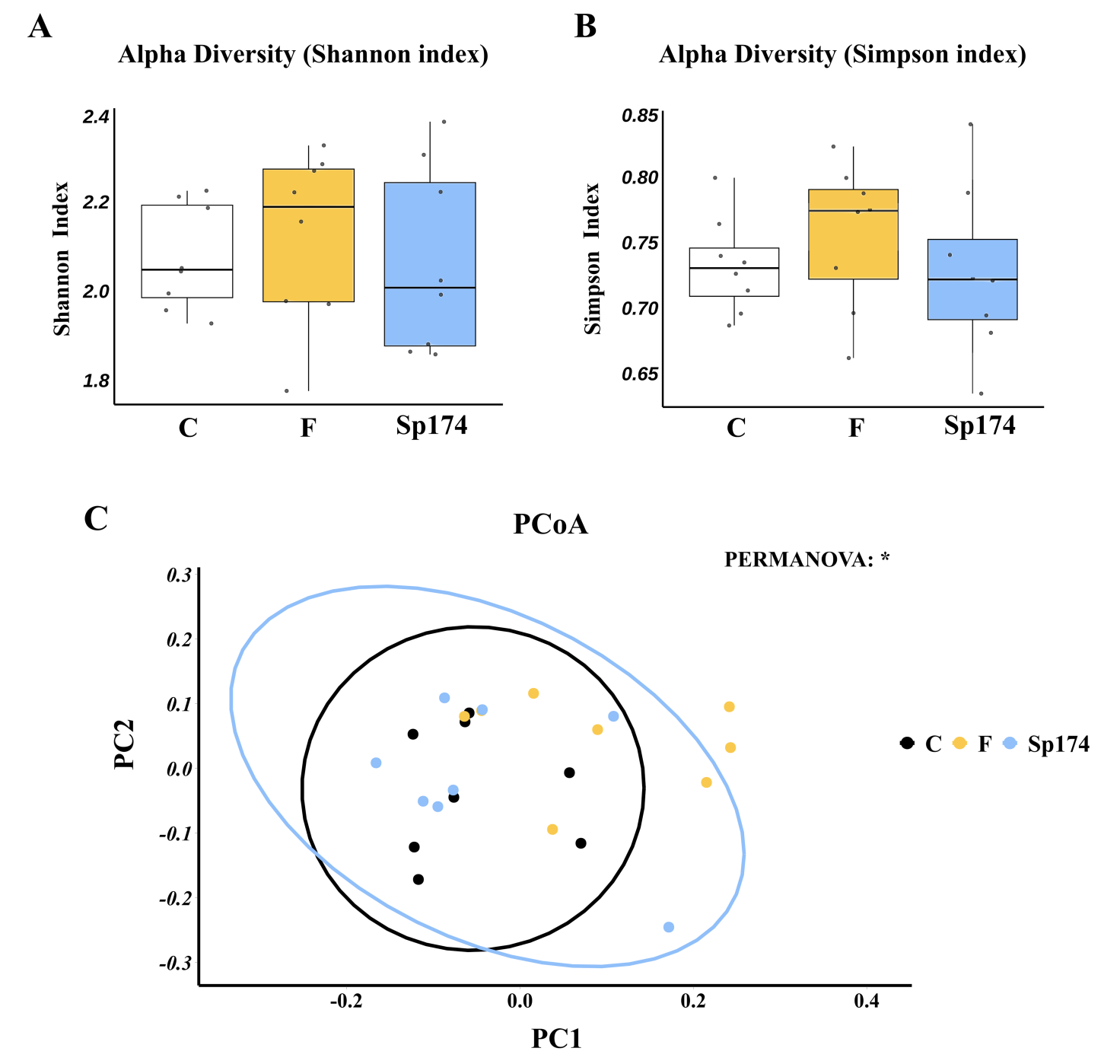
**

**Supplementary Fig. 4. Analysis of alpha and beta diversity**. The microbial taxa diversity based on Shannon (A) and Simpson (B) indices. Beta-diversity was analysed using Bray–Curtis distances (C). Plots were generated based on the weighted UniFrac distance matrix. Different experimental groups are indicated by various colors, as reported.


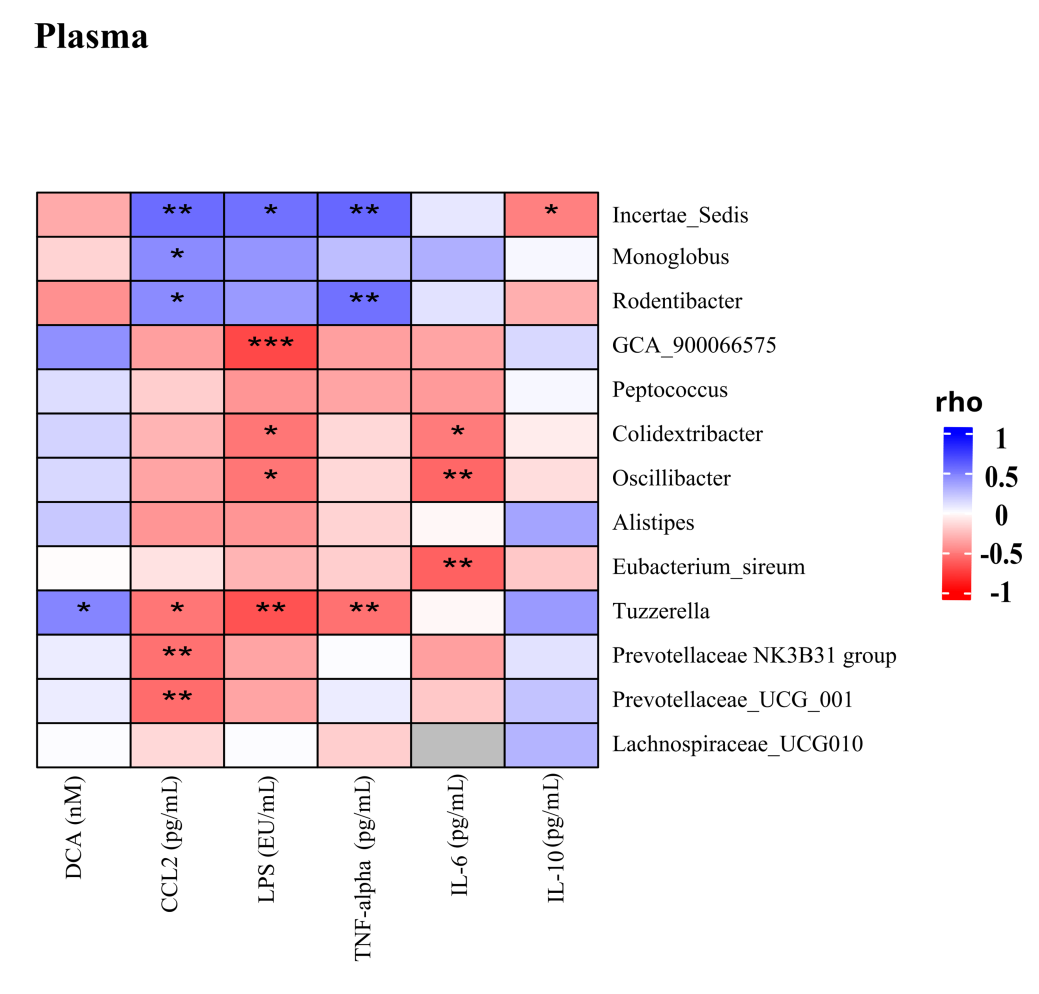


**Supplementary Fig.5. Spearman correlation heatmap between gut microbial genera and plasma inflammatory markers.** Heatmap showing pairwise Spearman correlation coefficients (ρ) between the relative abundances of selected gut microbial genera and plasma concentrations of DCA, IL-6, TNF-α, CCL2, IL-10 and LPS. Blue indicates positive correlations, red indicates negative correlations, and white represents values close to zero. Asterisks indicate statistical significance (*p < 0.05; **p < 0.01; ***p < 0.001).

**References**

1. Schmid R, Petras D, Nothias LF, Wang M, Aron AT, Jagels A, Tsugawa H, Rainer J, Garcia-Aloy M, Dührkop K, Korf A, Pluskal T, Kameník Z, Jarmusch AK, Caraballo-Rodríguez AM, Weldon KC, Nothias-Esposito M, Aksenov AA, Bauermeister A, Albarracin Orio A, Grundmann CO, Vargas F, Koester I, Gauglitz JM, Gentry EC, Hövelmann Y, Kalinina SA, Pendergraft MA, Panitchpakdi M, Tehan R, Le Gouellec A, Aleti G, Mannochio Russo H, Arndt B, Hübner F, Hayen H, Zhi H, Raffatellu M, Prather KA, Aluwihare LI, Böcker S, McPhail KL, Humpf HU, Karst U, Dorrestein PC. Ion identity molecular networking for mass spectrometry-based metabolomics in the GNPS environment. Nat Commun. 2021;12(1):3832; doi: 10.1038/s41467-021-23953-9.
