# Supplementary material for: Gut-brain axis: beneficial impact of Shouchella clausii spores on fructose induced dysfunction is associated with modulation of the deoxycholic acid – TGR5 pathway
Source: Mol Med. 2026 May 9;32:101. doi: 10.1186/s10020-026-01479-4 (PMC13326143; doi:10.1186/s10020-026-01479-4)
Supplement: Supplementary file 2 — Additional file 2. [file 10020_2026_1479_MOESM2_ESM.pptx]

## Slide 1
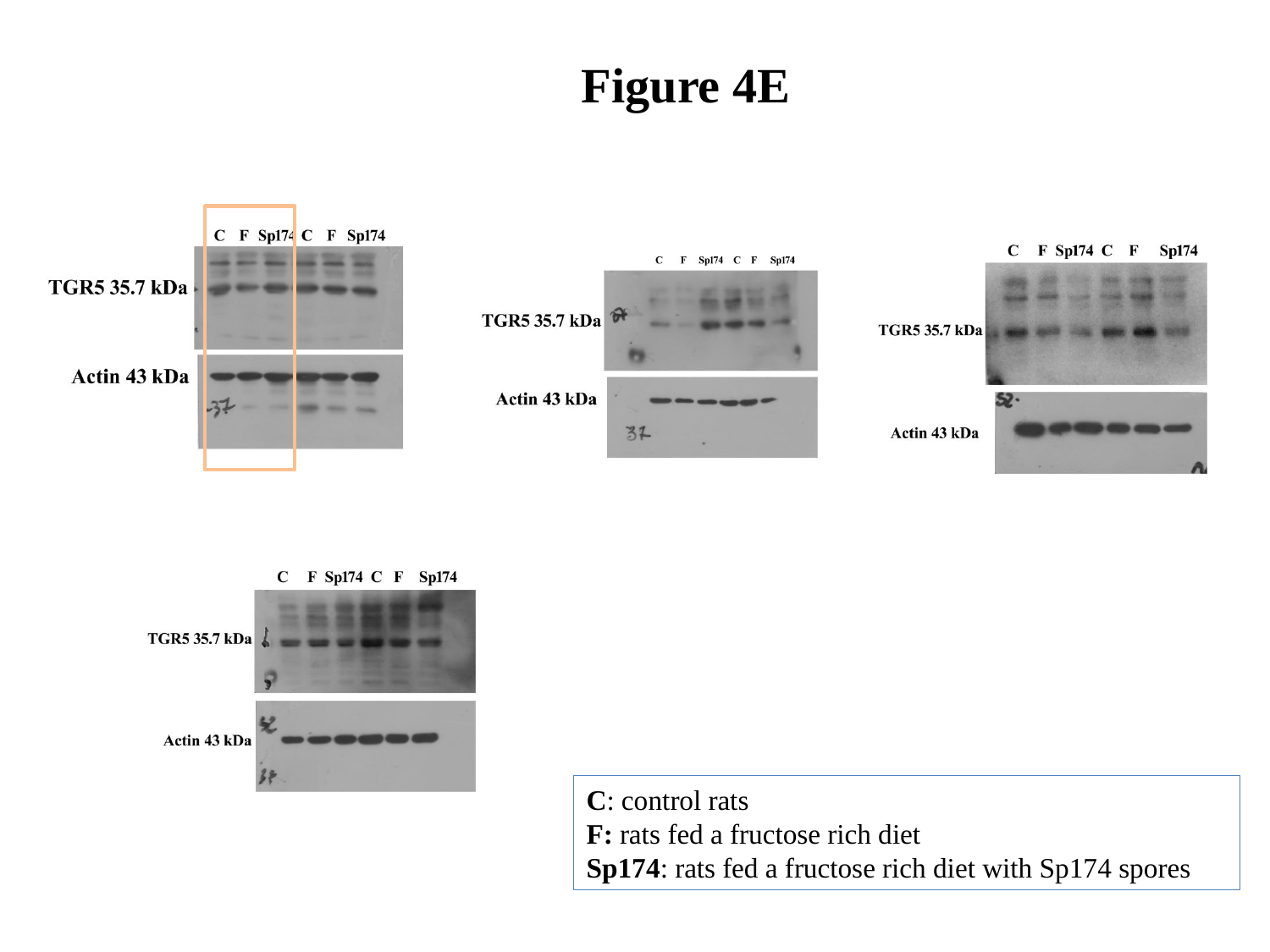

# Figure 4E
C: control rats
F: rats fed a fructose rich diet
Sp174: rats fed a fructose rich diet with Sp174 spores

## Slide 2
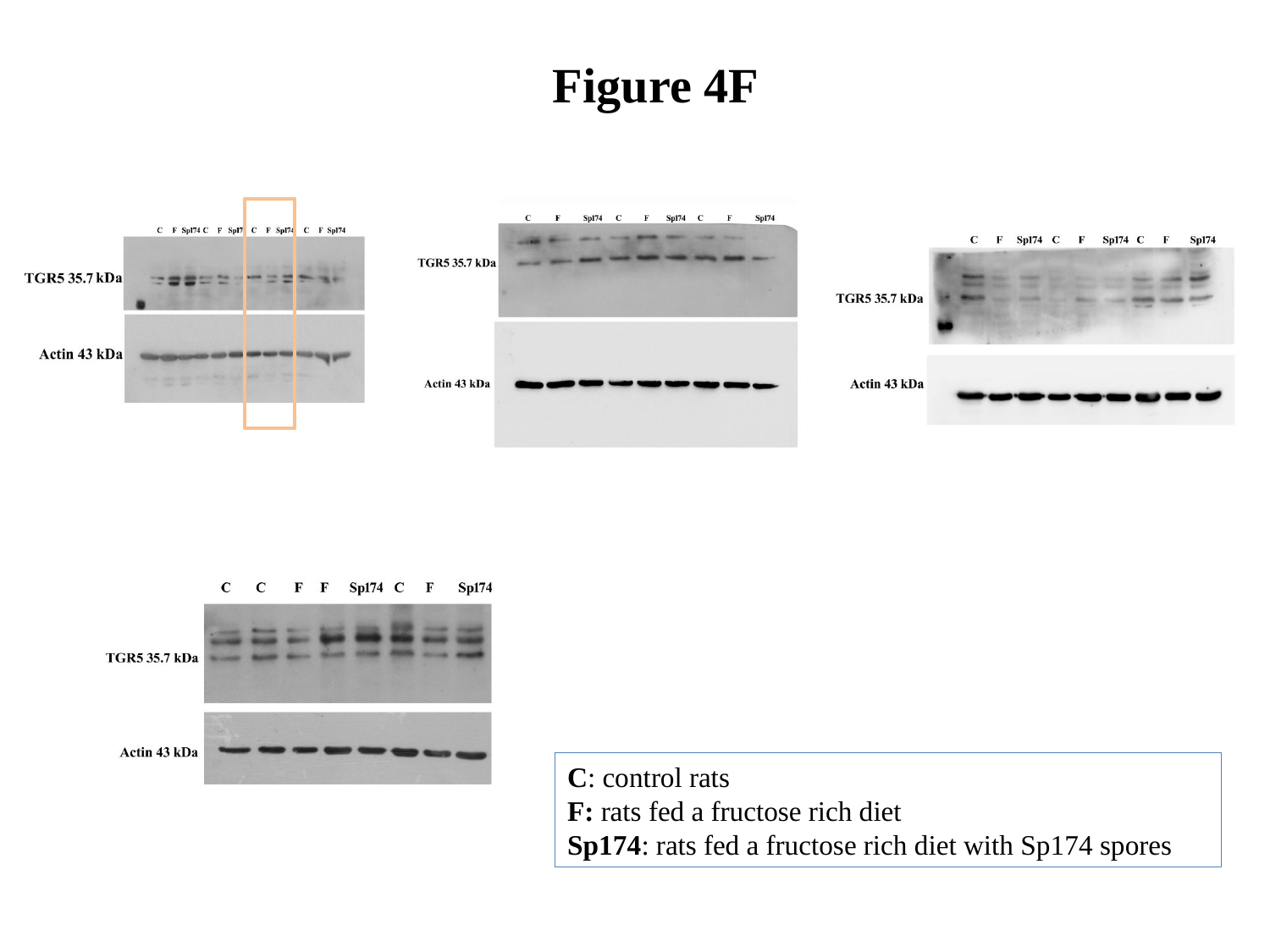

Figure 4F
C: control rats
F: rats fed a fructose rich diet
Sp174: rats fed a fructose rich diet with Sp174 spores

## Slide 3
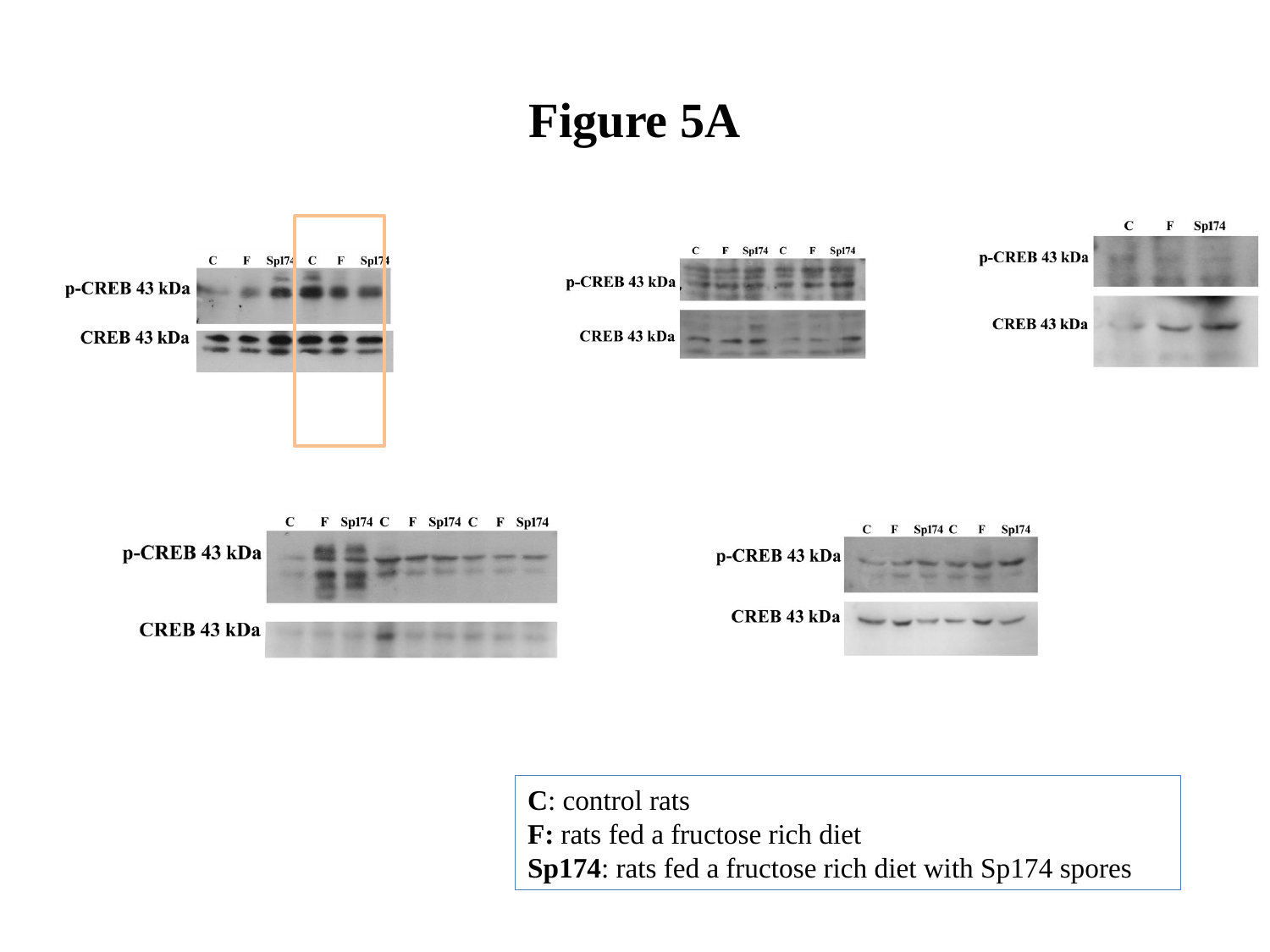

# Figure 5A
C: control rats
F: rats fed a fructose rich diet
Sp174: rats fed a fructose rich diet with Sp174 spores

## Slide 4
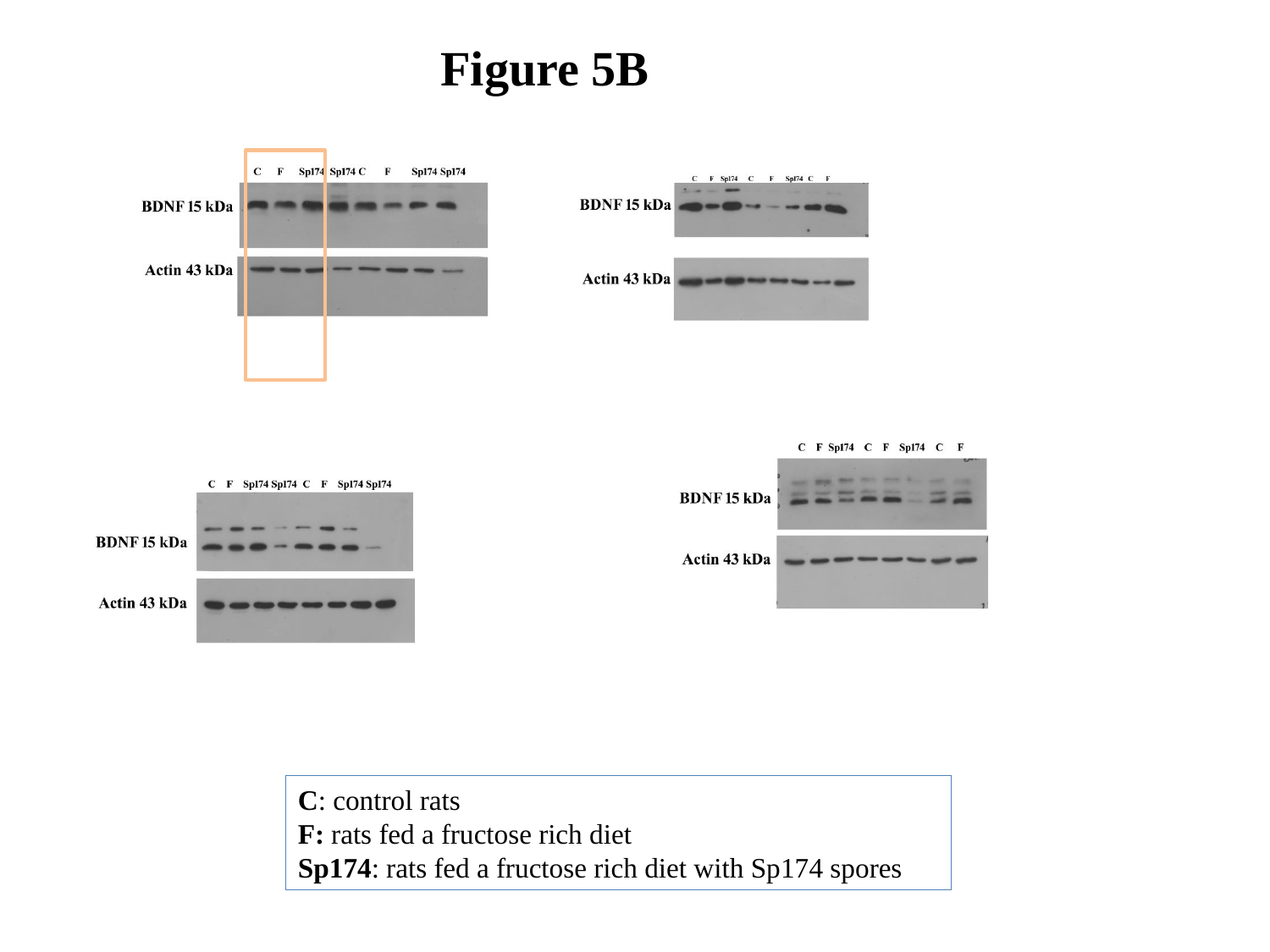

Figure 5B
C: control rats
F: rats fed a fructose rich diet
Sp174: rats fed a fructose rich diet with Sp174 spores

## Slide 5
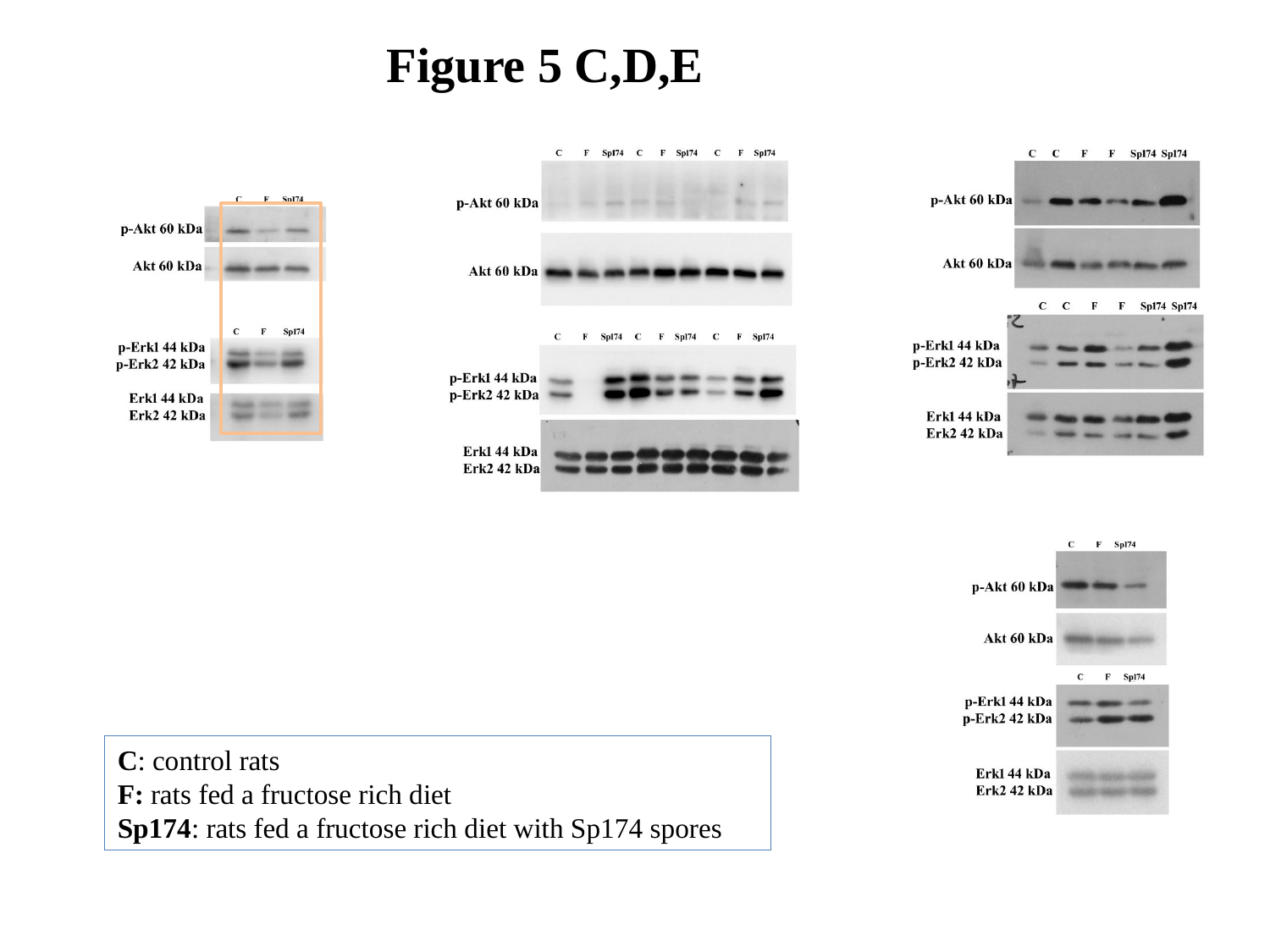

Figure 5 C,D,E
C: control rats
F: rats fed a fructose rich diet
Sp174: rats fed a fructose rich diet with Sp174 spores

## Slide 6
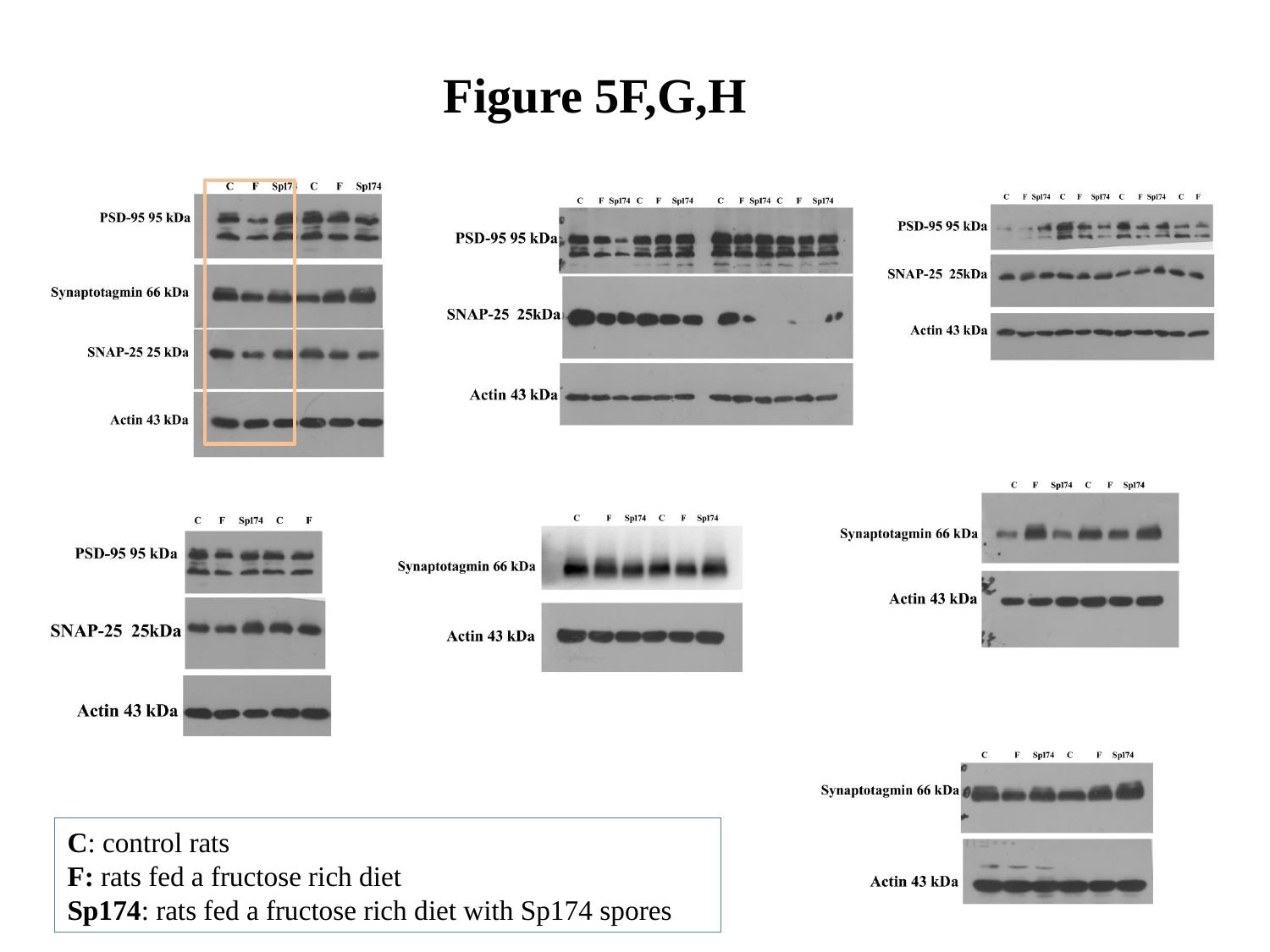

Figure 5F,G,H
C: control rats
F: rats fed a fructose rich diet
Sp174: rats fed a fructose rich diet with Sp174 spores

## Slide 7
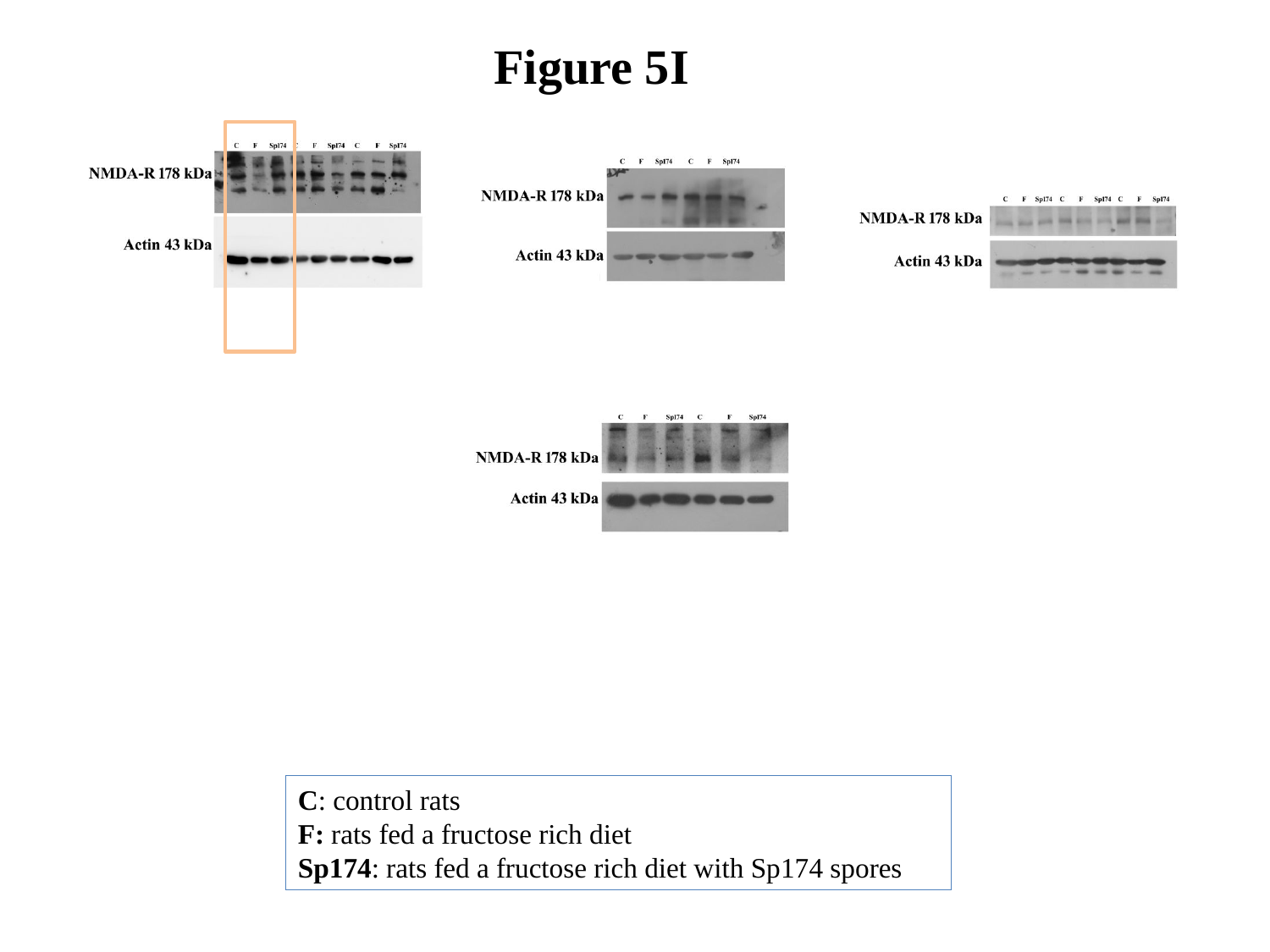

Figure 5I
C: control rats
F: rats fed a fructose rich diet
Sp174: rats fed a fructose rich diet with Sp174 spores

## Slide 8
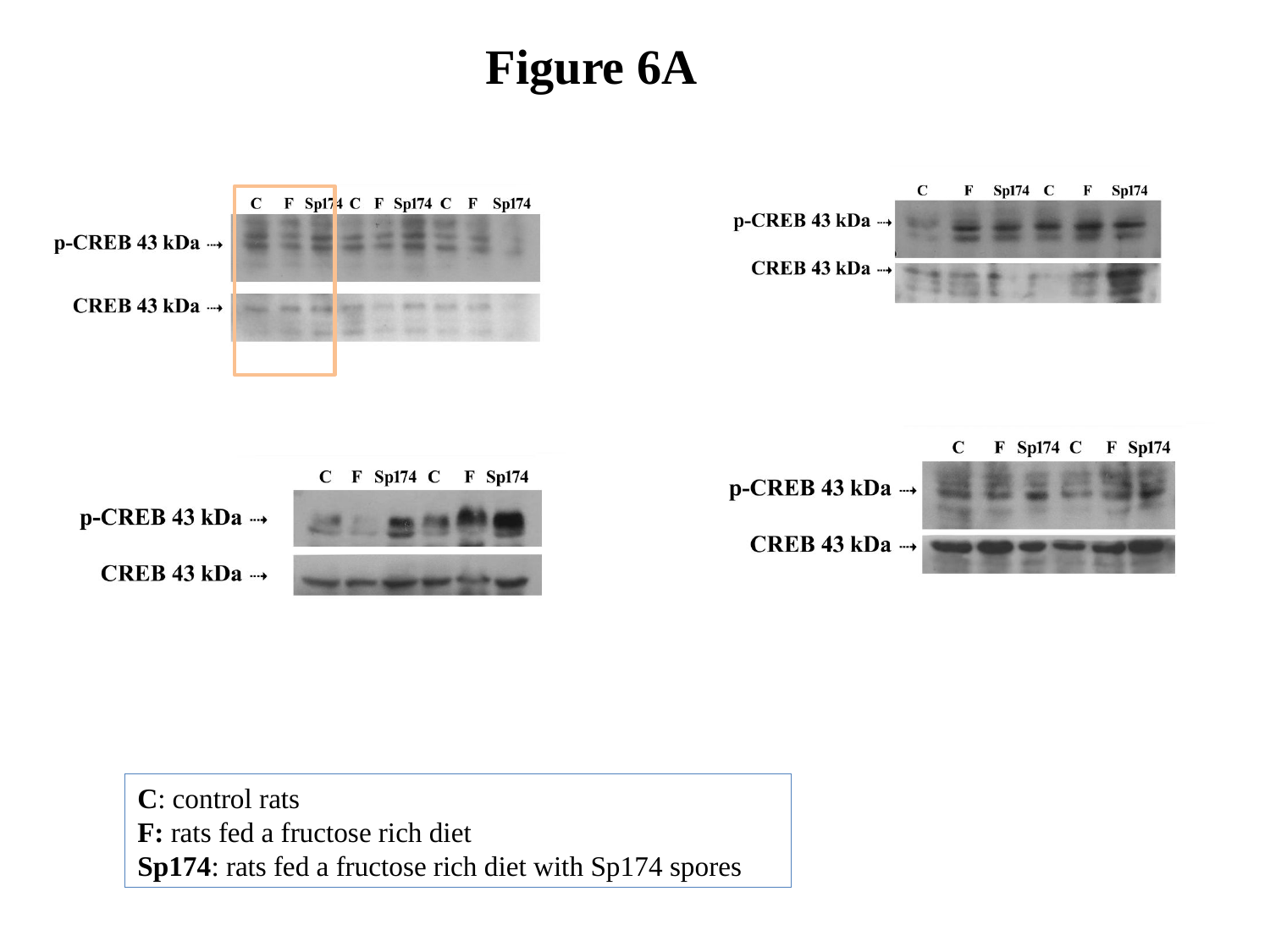

Figure 6A
C: control rats
F: rats fed a fructose rich diet
Sp174: rats fed a fructose rich diet with Sp174 spores

## Slide 9
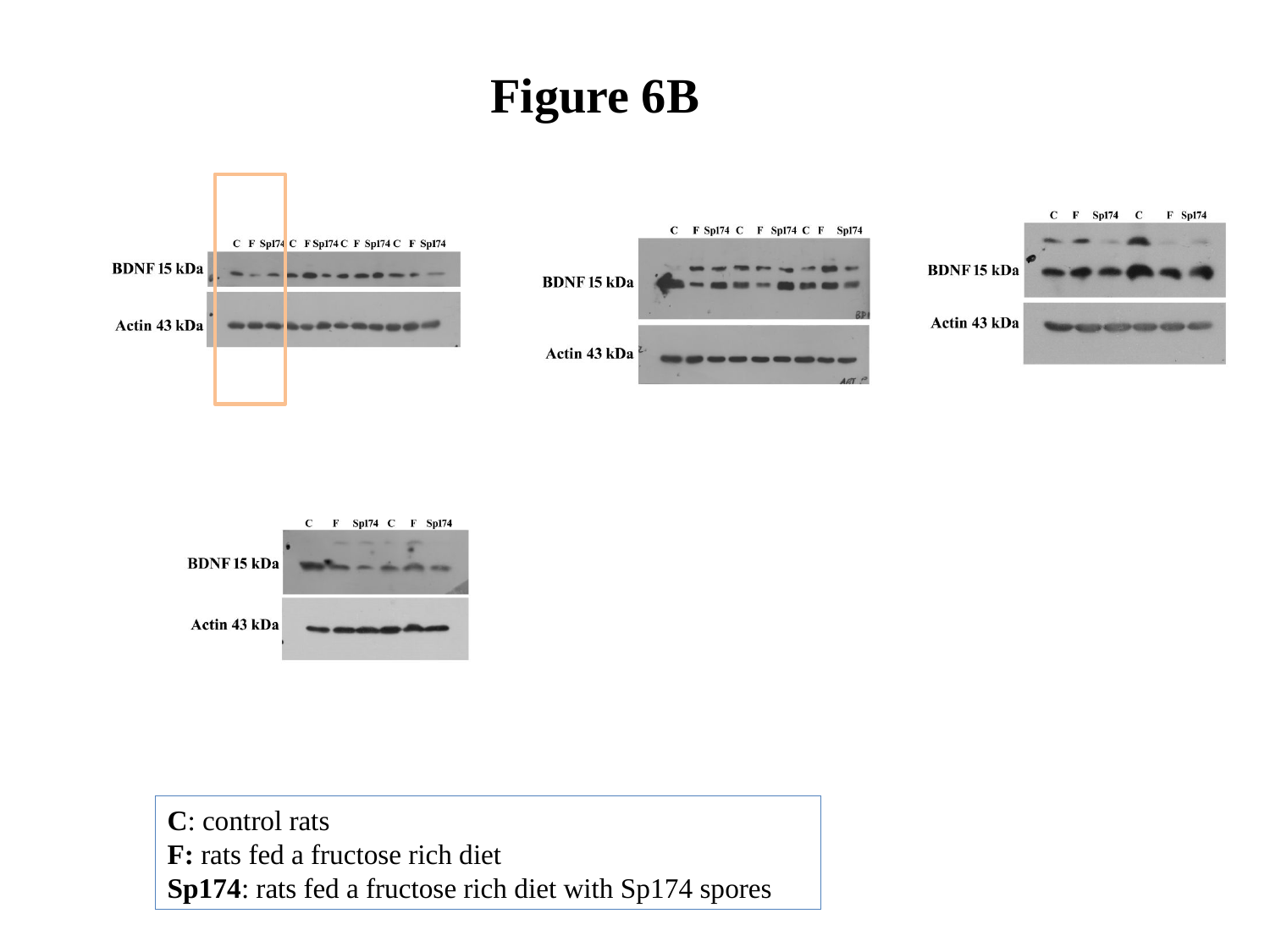

Figure 6B
C: control rats
F: rats fed a fructose rich diet
Sp174: rats fed a fructose rich diet with Sp174 spores

## Slide 10
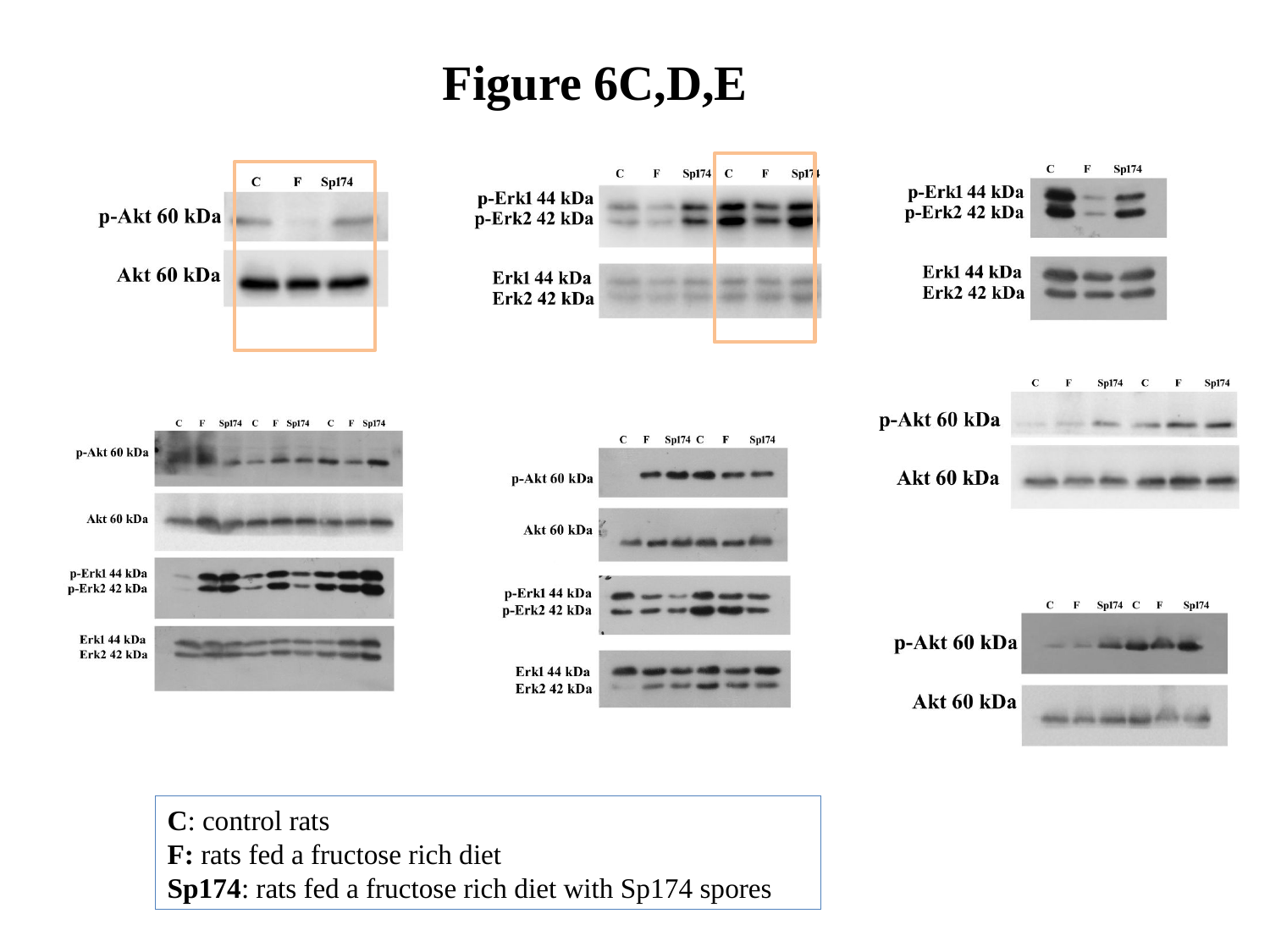

Figure 6C,D,E
C: control rats
F: rats fed a fructose rich diet
Sp174: rats fed a fructose rich diet with Sp174 spores

## Slide 11
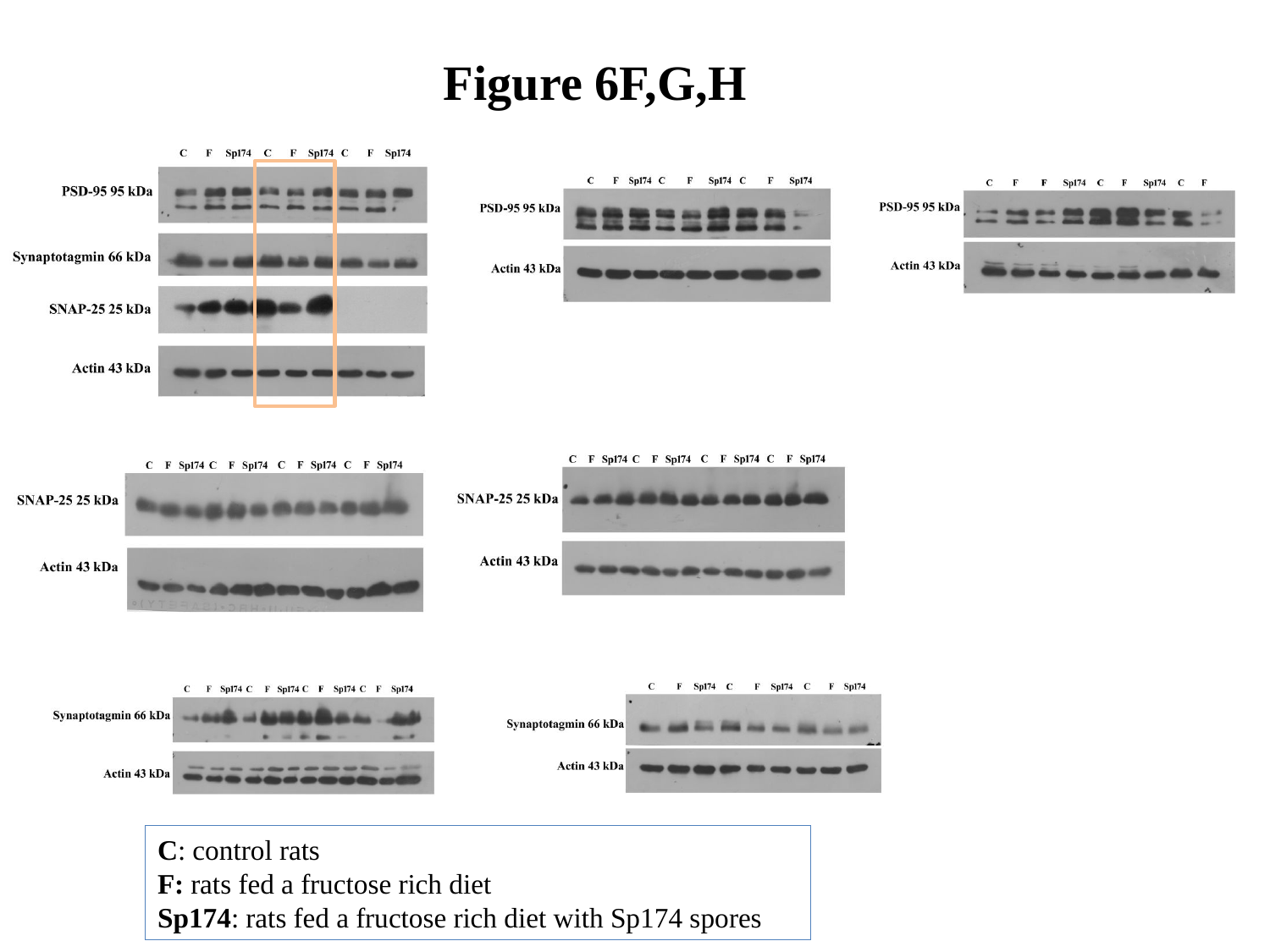

Figure 6F,G,H
C: control rats
F: rats fed a fructose rich diet
Sp174: rats fed a fructose rich diet with Sp174 spores

## Slide 12
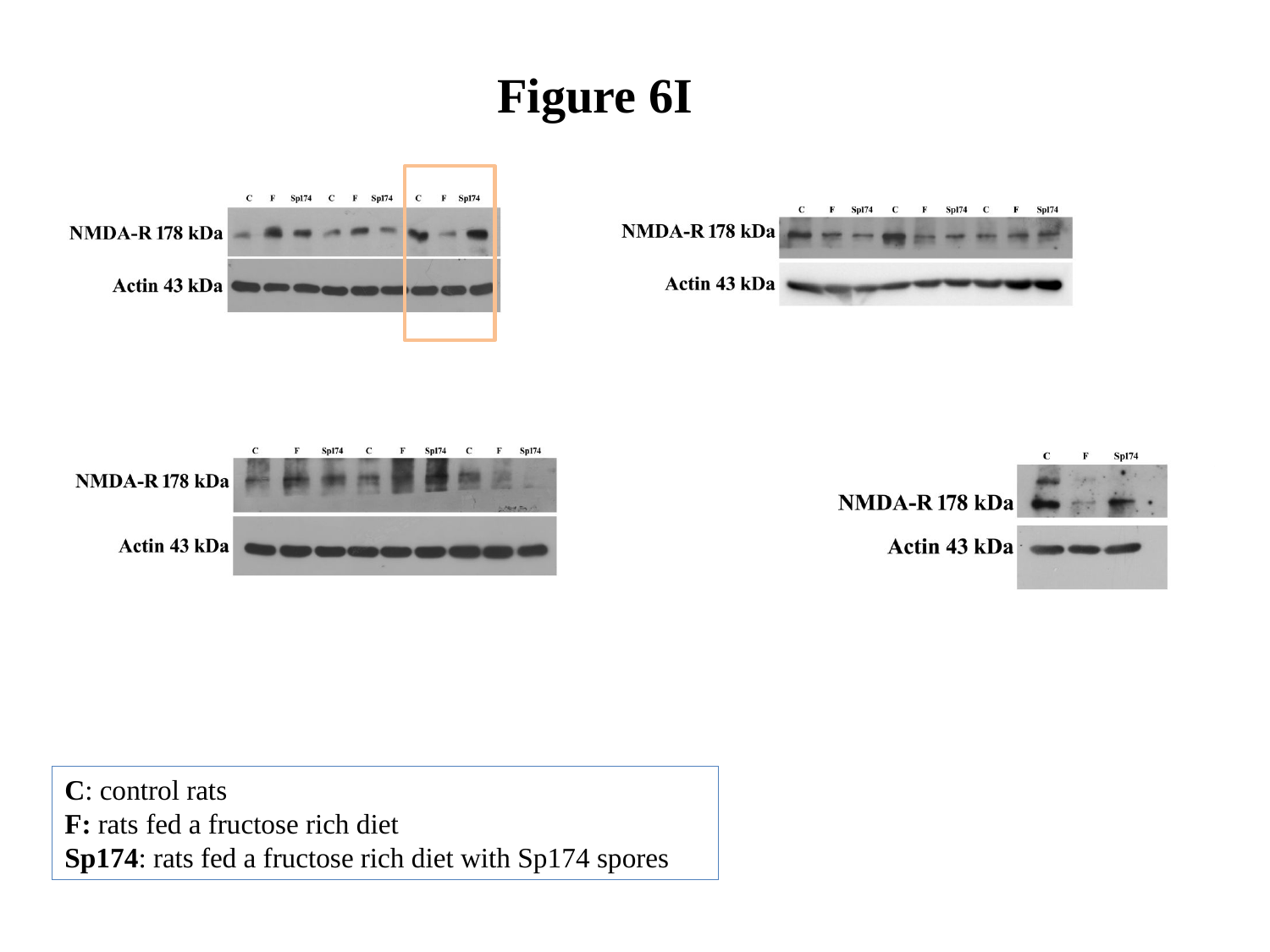

Figure 6I
C: control rats
F: rats fed a fructose rich diet
Sp174: rats fed a fructose rich diet with Sp174 spores

## Slide 13
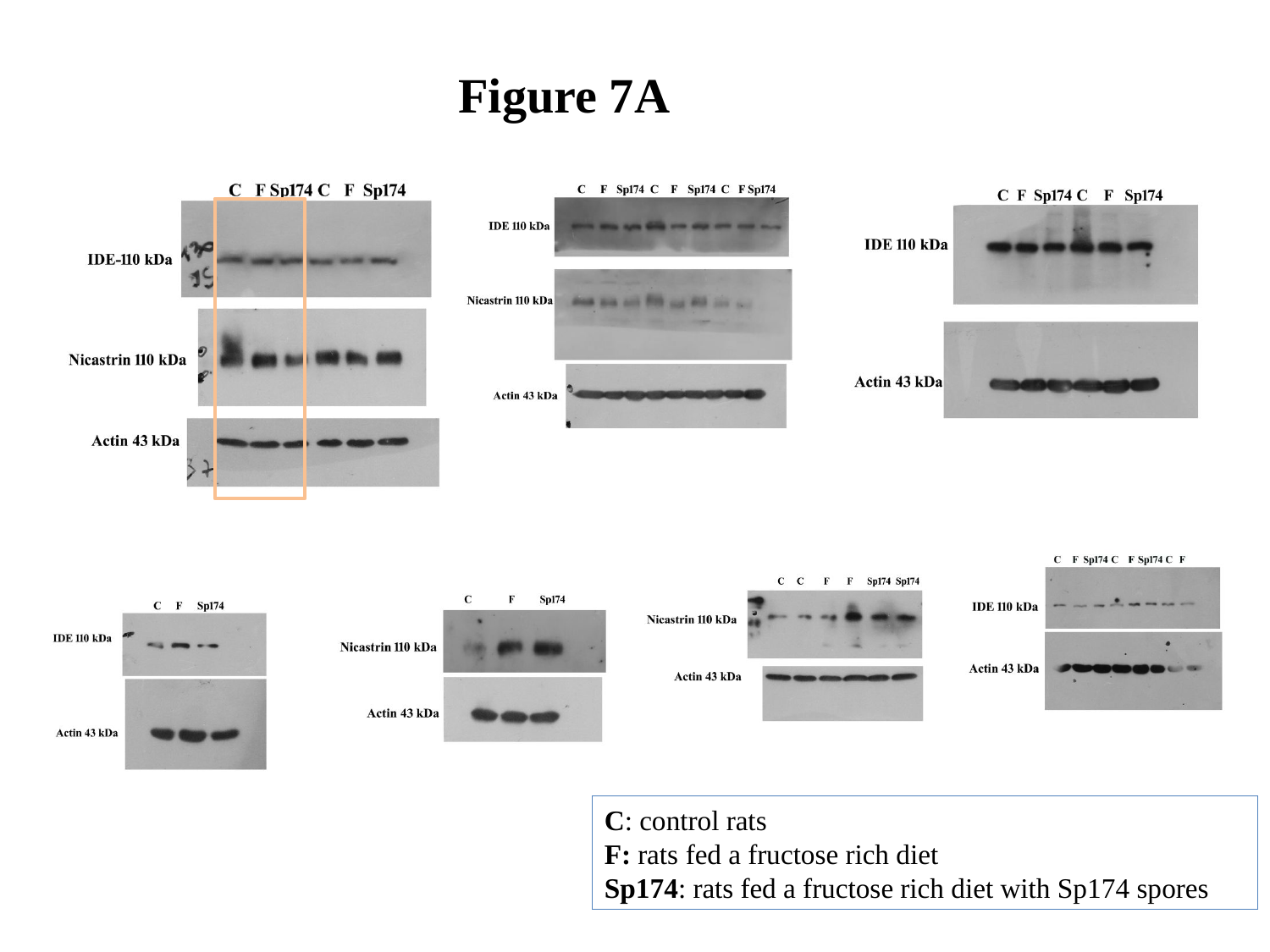

# Figure 7A
C: control rats
F: rats fed a fructose rich diet
Sp174: rats fed a fructose rich diet with Sp174 spores

## Slide 14
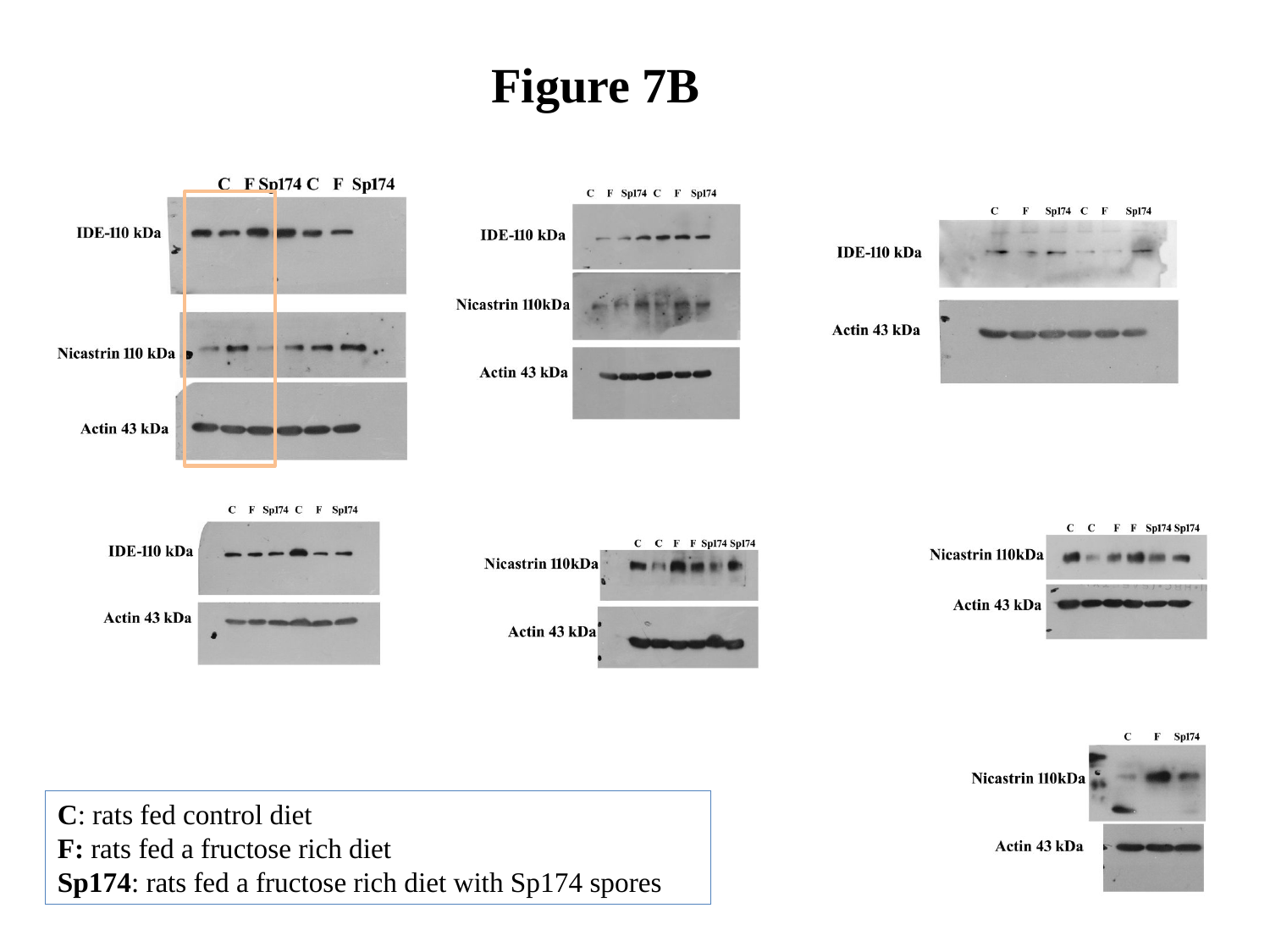

# Figure 7B
C: rats fed control diet
F: rats fed a fructose rich diet
Sp174: rats fed a fructose rich diet with Sp174 spores

## Slide 15
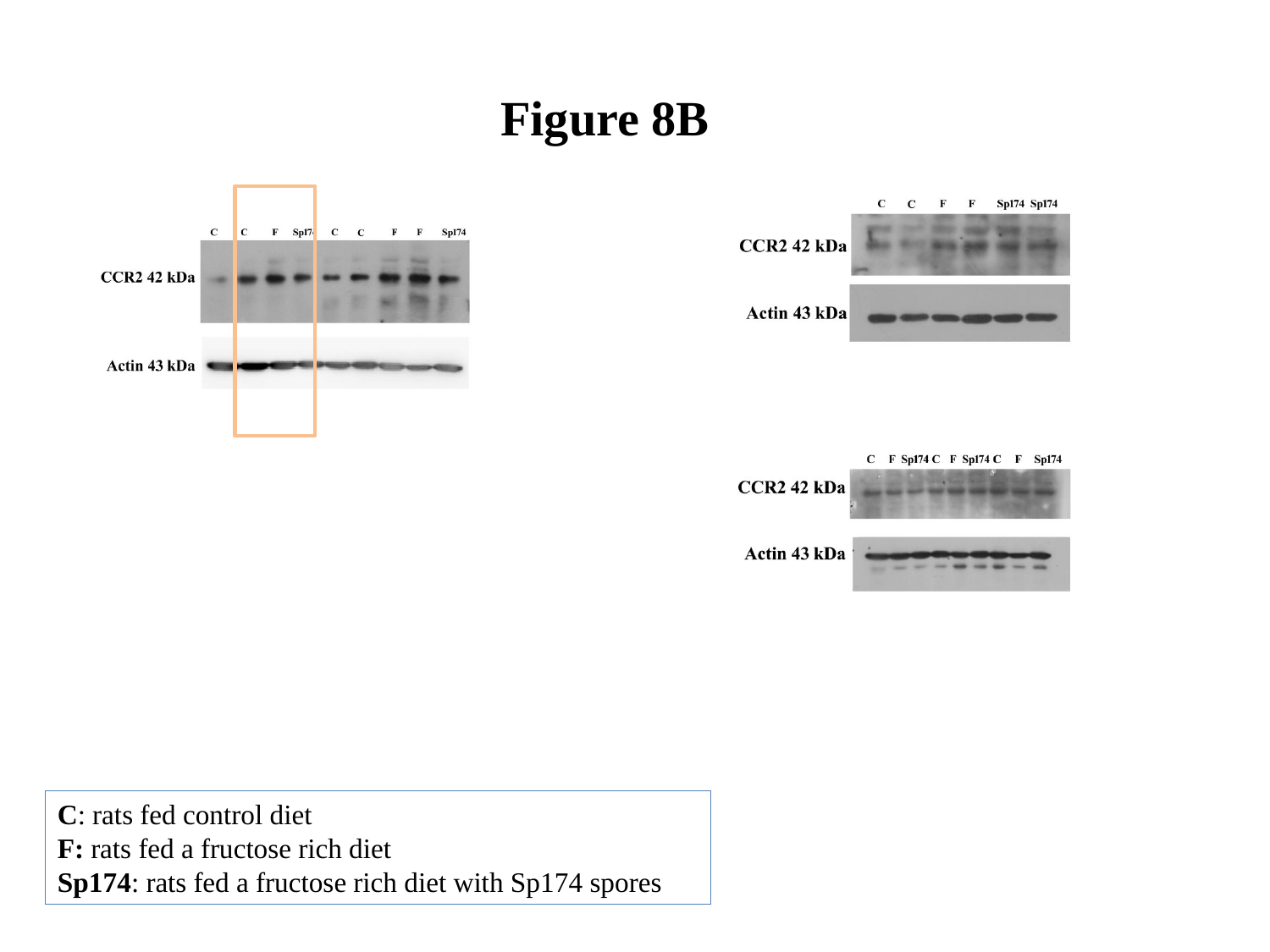

# Figure 8B
C: rats fed control diet
F: rats fed a fructose rich diet
Sp174: rats fed a fructose rich diet with Sp174 spores

## Slide 16
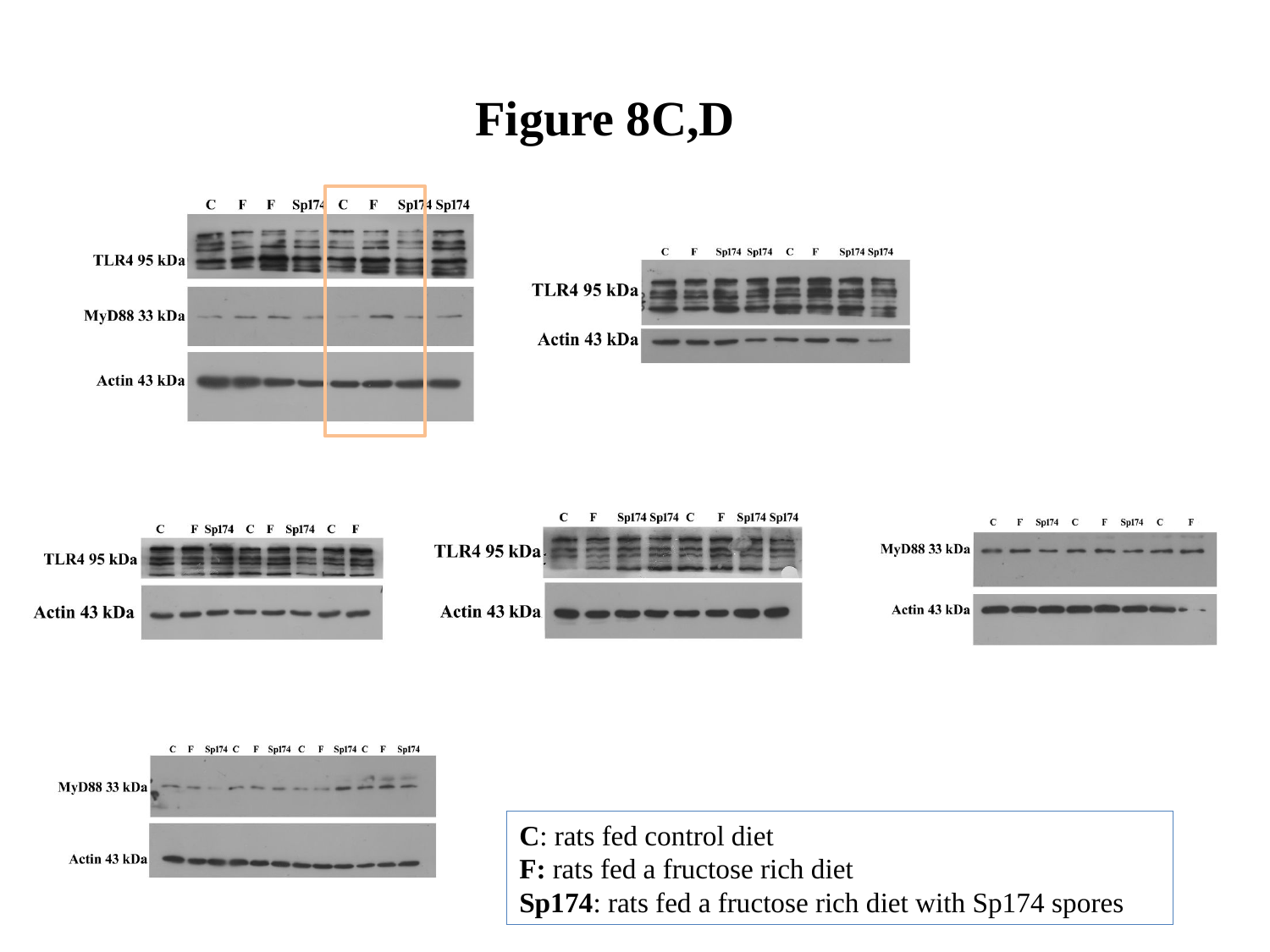

# Figure 8C,D
C: rats fed control diet
F: rats fed a fructose rich diet
Sp174: rats fed a fructose rich diet with Sp174 spores

## Slide 17
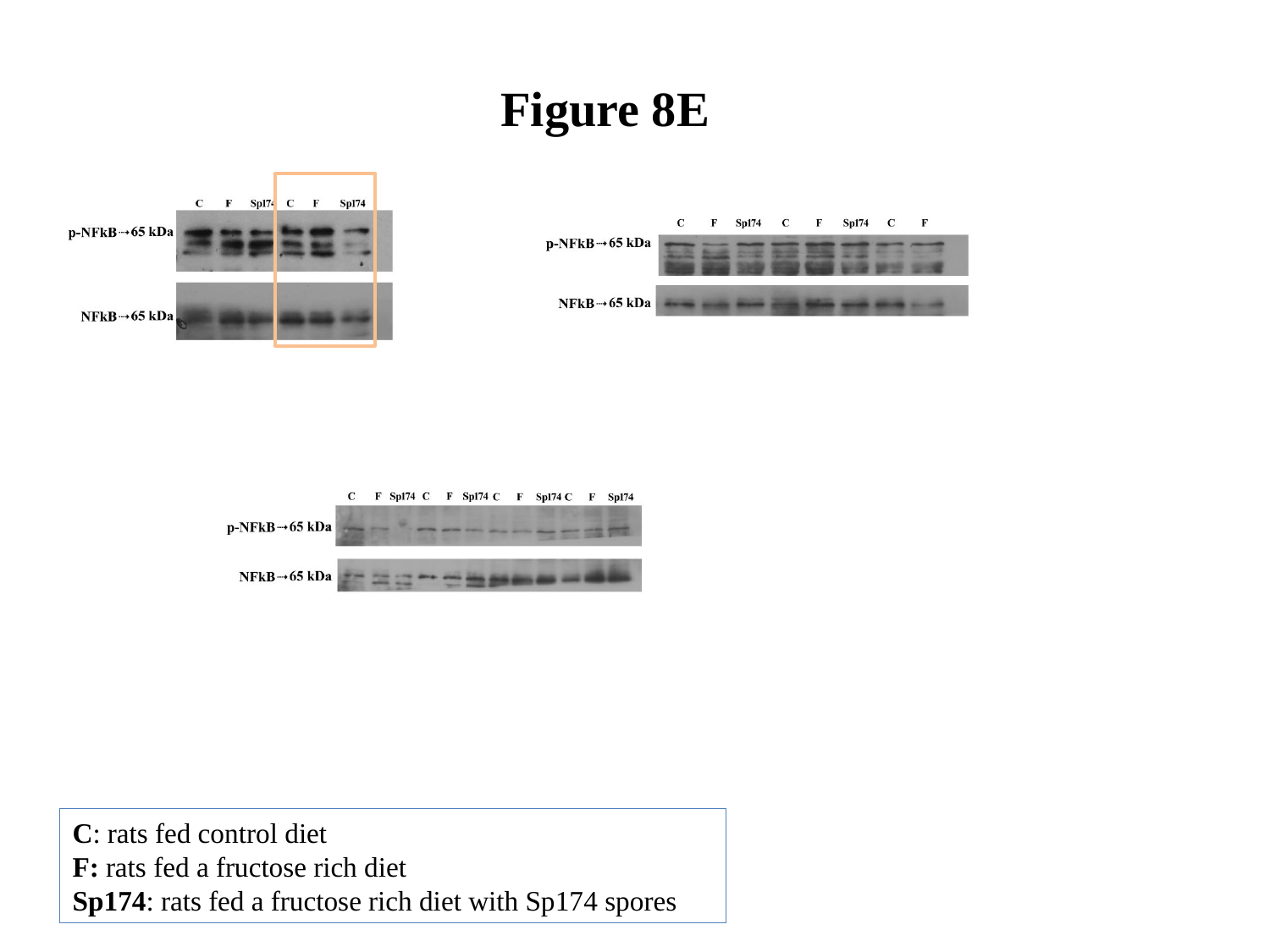

Figure 8E
C: rats fed control diet
F: rats fed a fructose rich diet
Sp174: rats fed a fructose rich diet with Sp174 spores

## Slide 18
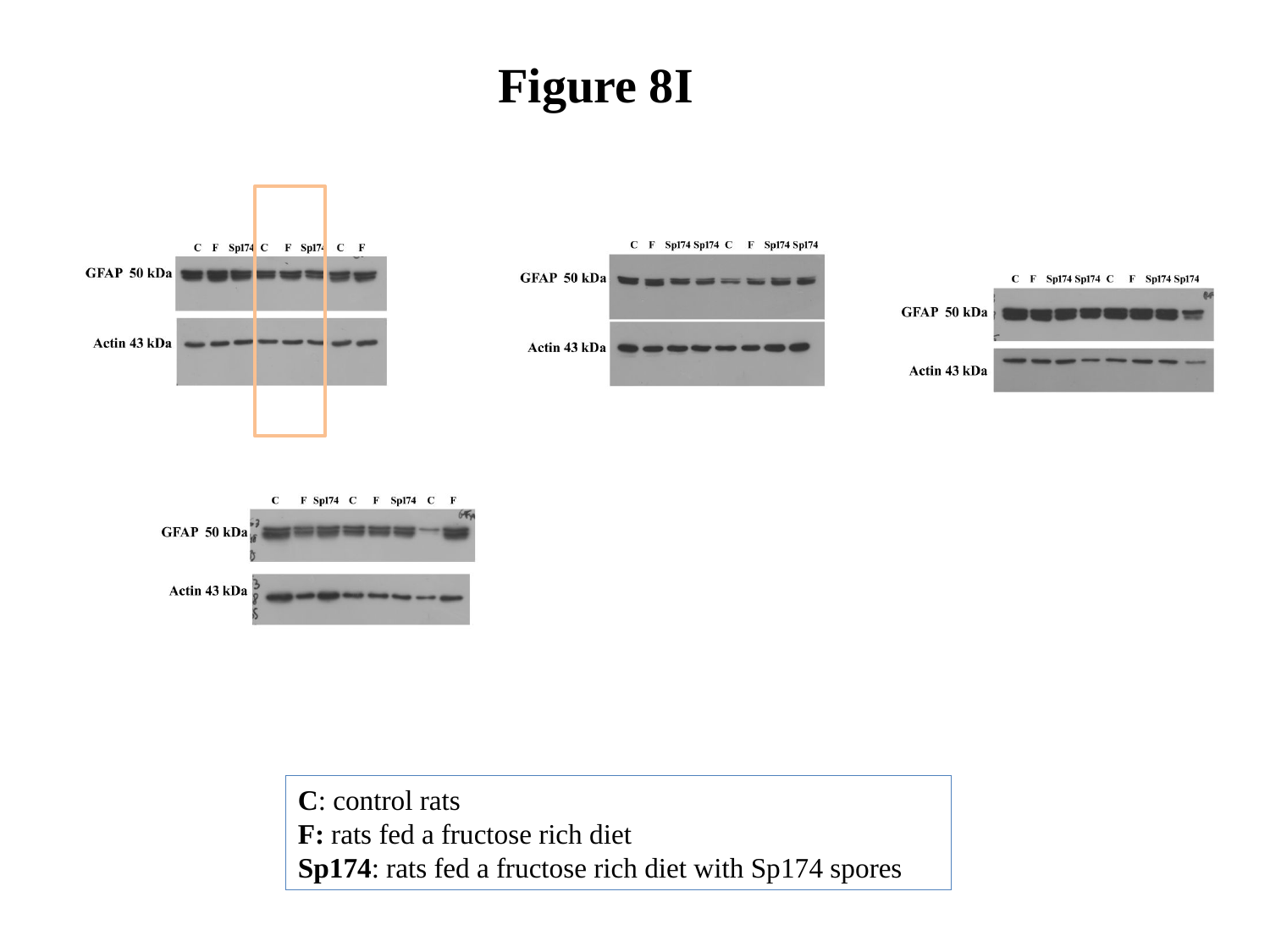

Figure 8I
C: control rats
F: rats fed a fructose rich diet
Sp174: rats fed a fructose rich diet with Sp174 spores

## Slide 19
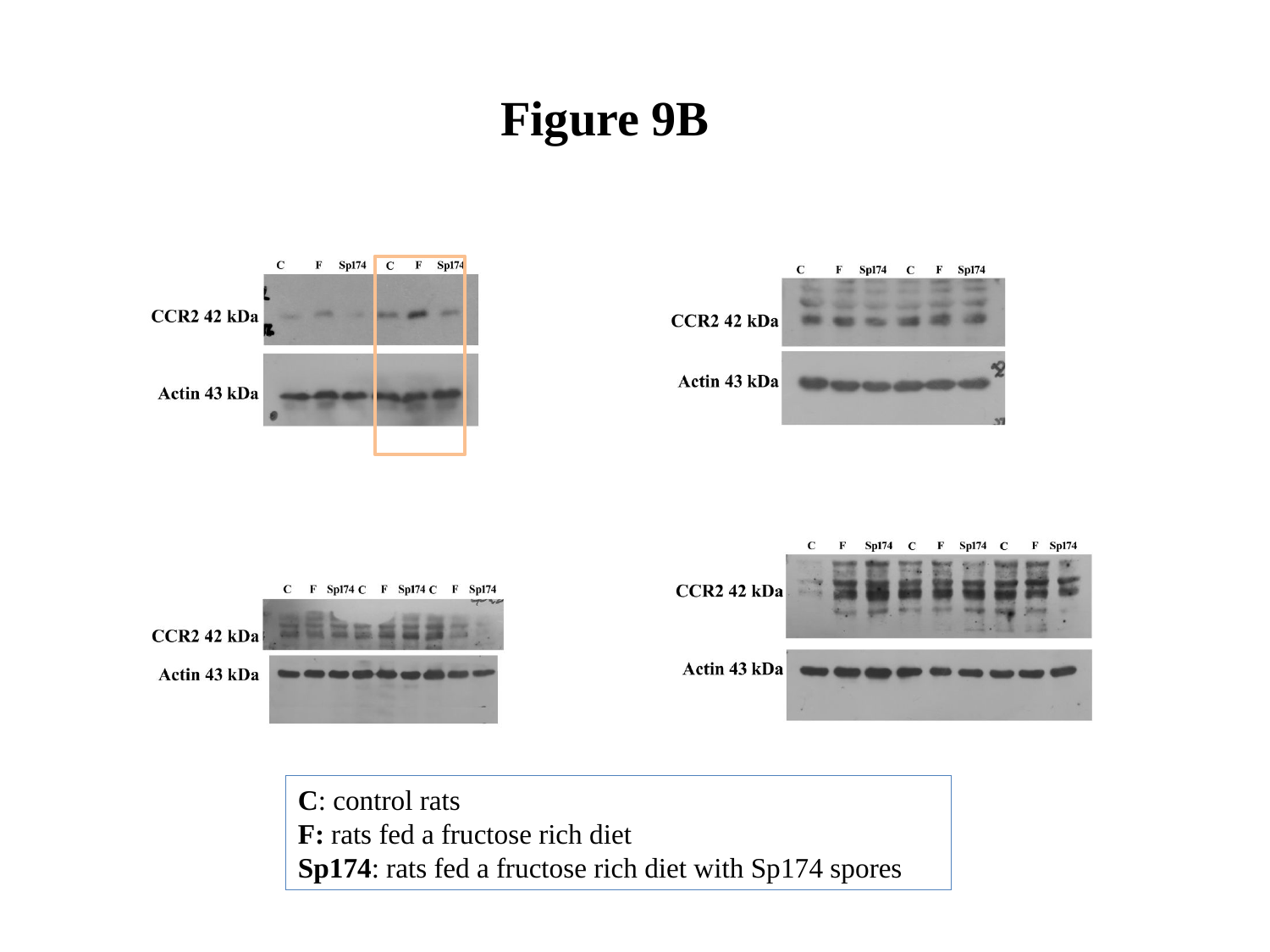

Figure 9B
C: control rats
F: rats fed a fructose rich diet
Sp174: rats fed a fructose rich diet with Sp174 spores

## Slide 20
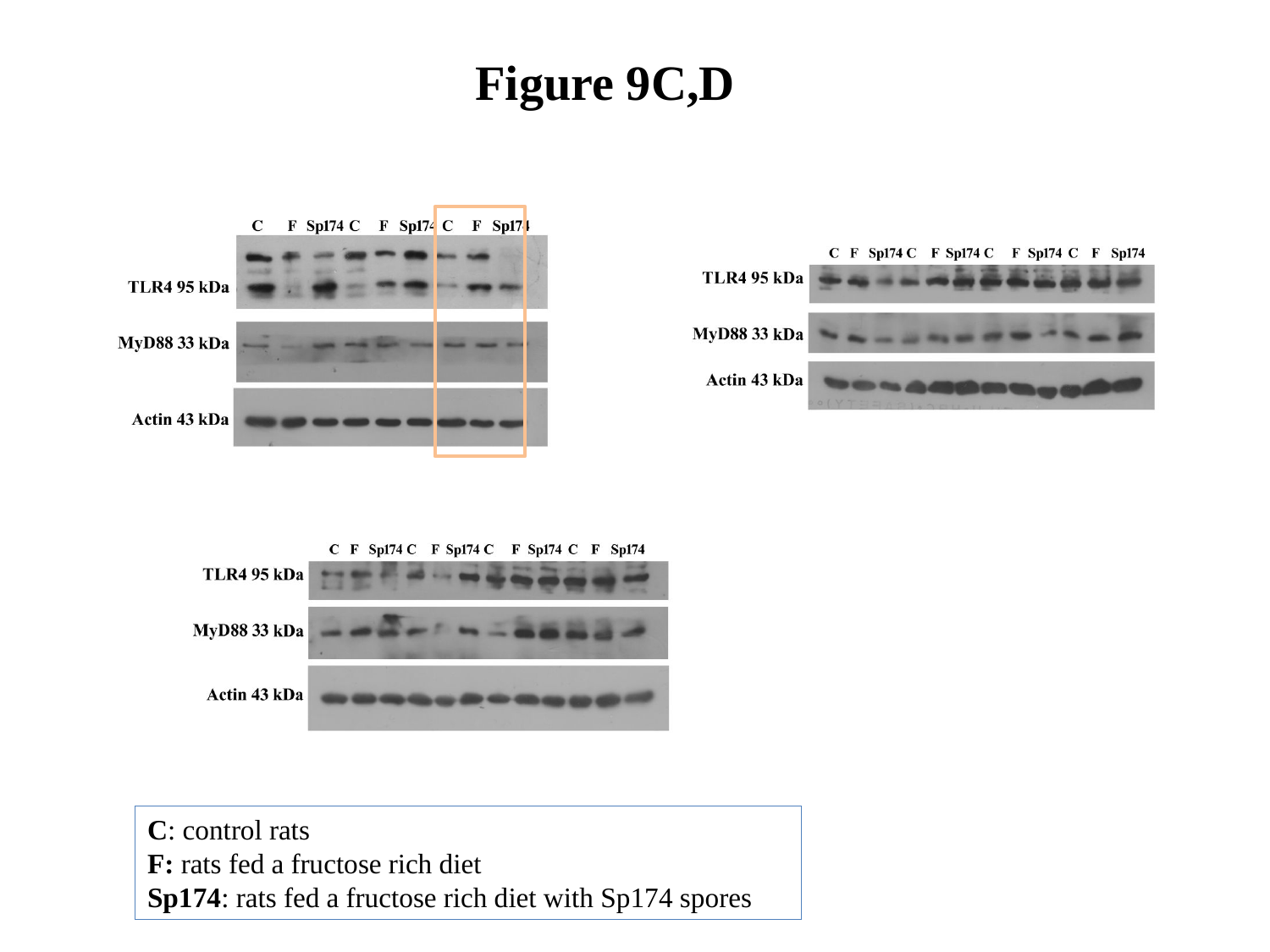

Figure 9C,D
C: control rats
F: rats fed a fructose rich diet
Sp174: rats fed a fructose rich diet with Sp174 spores

## Slide 21
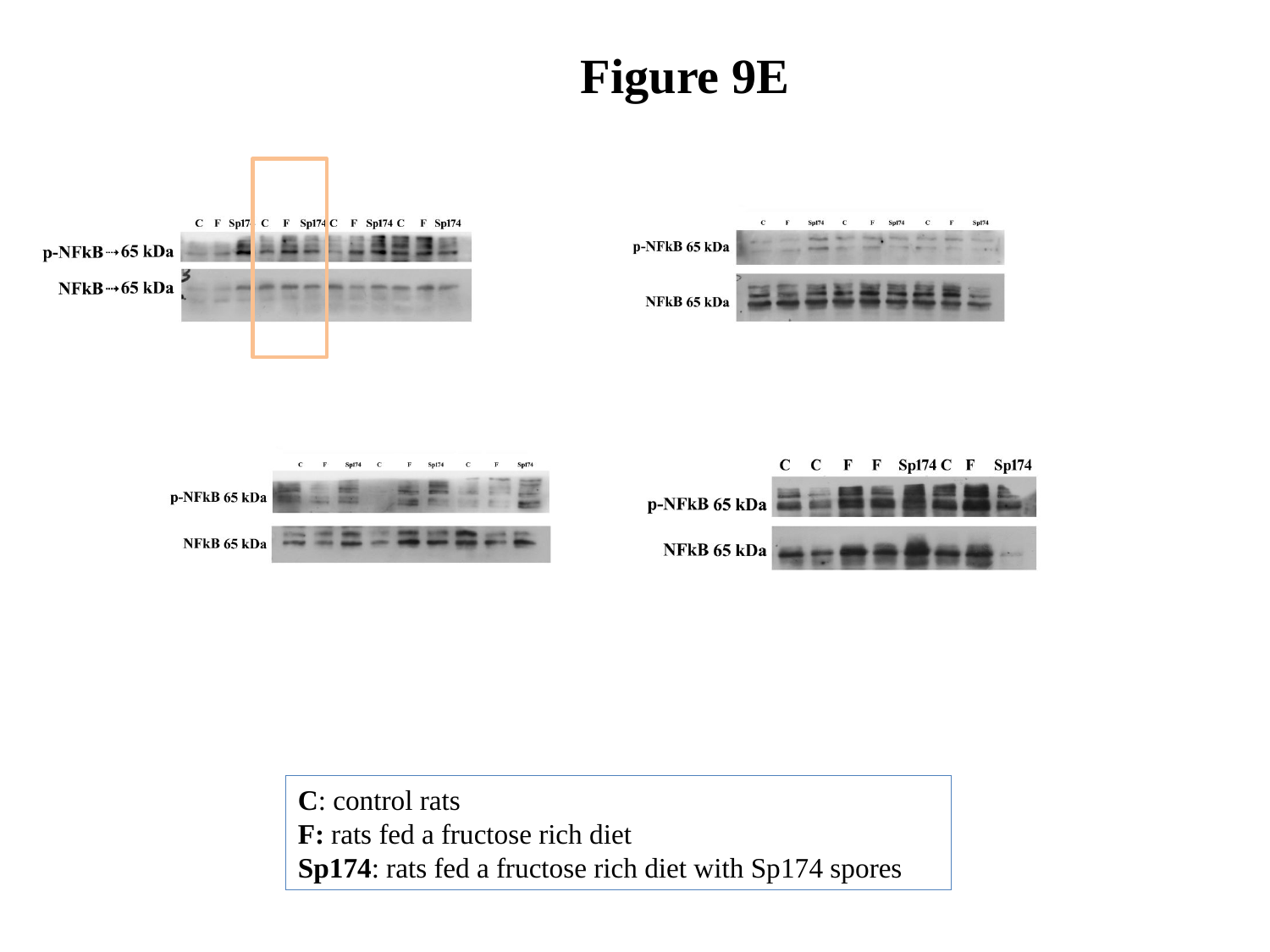

Figure 9E
C: control rats
F: rats fed a fructose rich diet
Sp174: rats fed a fructose rich diet with Sp174 spores

## Slide 22
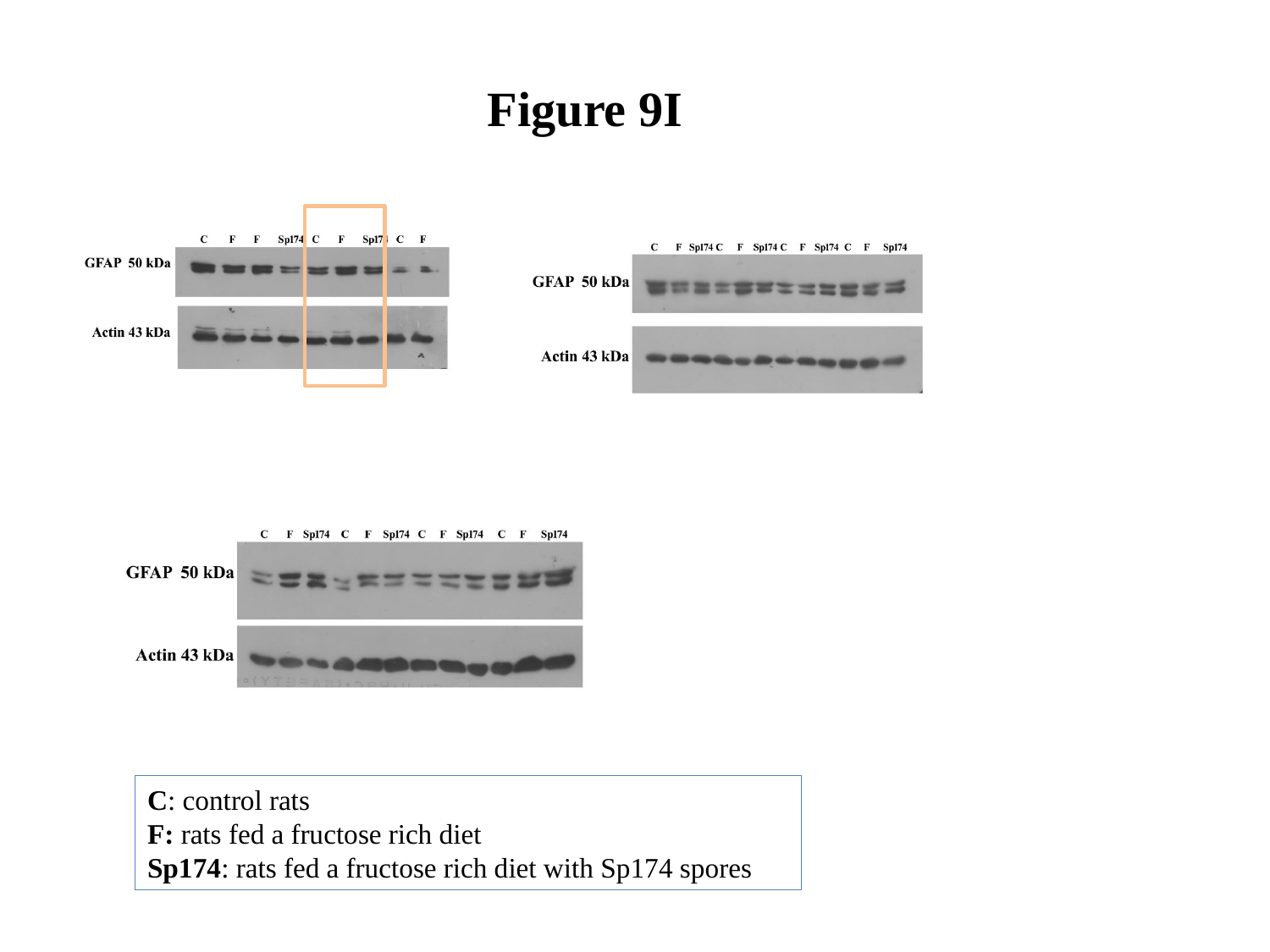

Figure 9I
C: control rats
F: rats fed a fructose rich diet
Sp174: rats fed a fructose rich diet with Sp174 spores
